# “It Is Not Possible to Balance It Easily”: A Phenomenological Study Exploring the Experience of Work–Family Conflict in Contemporary Chinese Society

**DOI:** 10.3390/bs16010063

**Published:** 2025-12-30

**Authors:** Shujie Chen, Mei-I Cheng, Shira Elqayam, Mark Scase

**Affiliations:** 1 Department of Business Administration, School of Business, Sun Yat-sen University, Guangzhou 510275, China; 2 School of Applied Social Sciences, Faculty of Health and Life Sciences, De Montfort University, Leicester LE1 9BH, UK; selqayam@dmu.ac.uk (S.E.); mscase@dmu.ac.uk (M.S.)

**Keywords:** cultural differences, phenomenological method, work–family conflict

## Abstract

This qualitative study aimed to explore the work–family conflict phenomenon in China, to extend our understanding of such a phenomenon experienced under a different cultural background outside of the West, and to help suggest the Chinese culturally specific variables (e.g., filial piety) related to the work–family conflict in China for future research. A purposive sample of 16 Chinese employees was interviewed. Using Creswell’s phenomenological method, six themes and 17 sub-themes emerged through 297 significant statements. The participants described the work–family conflict as only a life experience or no more than a minor problem in life that has influenced their coping strategy (e.g., avoidance coping). It appeared that Chinese culture places both positive and negative effects that simultaneously ease and exacerbate work–family conflict (e.g., a greater level of family support came with more family obligation). After comparing the results with the previous Western findings, differences in the experience of work–family conflict were identified. Relevant factors related to the experience of work–family conflict were suggested, providing directions for future work–family conflict studies.

## 1. Introduction

The intensive work environment in China, characterised by long working hours, heavy workloads, and strong expectations for professional dedication, has led to growing concern regarding work–family conflict (WFC), defined as an inter-role conflict in which work and family demands are mutually incompatible (e.g., Wang et al., 2020; M. Zhang et al., 2019). Although research on WFC in China has expanded in recent years (e.g., Lu & Cooper, 2015; Allen et al., 2020), the field remains shaped largely by Western-derived theories and models that may not sufficiently capture culturally embedded meanings of work, family, and role obligations. For example, Lu and Cooper (2015) note that 95 per cent of WFC research has been conducted based on Western contexts, indicating the continuing need to examine how WFC is experienced and interpreted in cultural environments where work and family expectations are organised differently.

In response to this gap, scholars have emphasised the importance of considering contextual and culturally rooted factors when investigating WFC (e.g., S. Chen et al., 2023; Agars & French, 2011; Kossek et al., 2011). A qualitative approach is particularly valuable in this regard, as it allows individuals to articulate how they make sense of WFC within their own sociocultural context, including how cultural norms influence perceptions of whether WFC counts as an issue or merely a daily experience. Qualitative inquiry thus contributes not only to documenting WFC, but to exploring how meaning, norms, and values shape such an experience (Agars & French, 2011), providing insights that may be omitted in quantitative frameworks.

**Figure 1:**
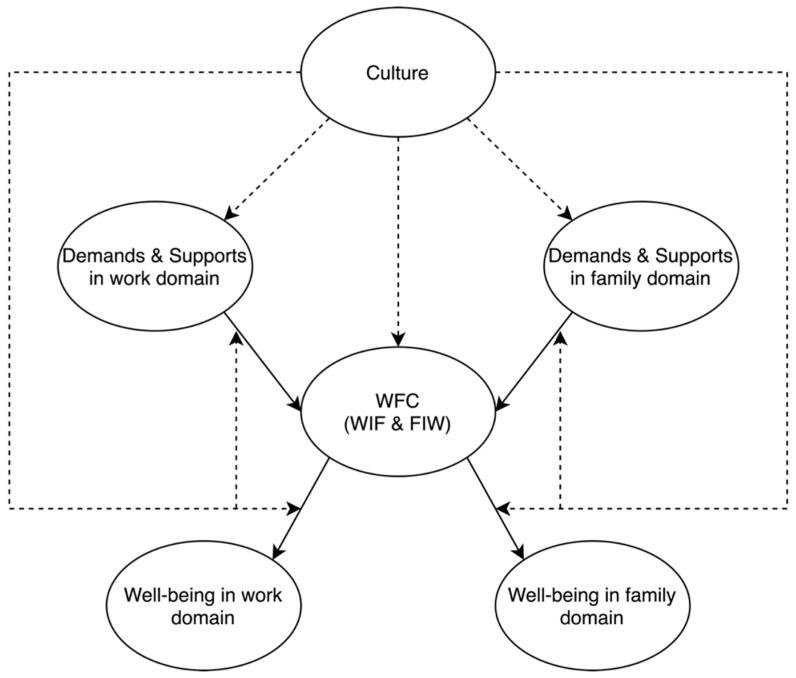
A redraw based on Aycan’s (2008) Culture and Work-Family Conflict Model.

Hence, this qualitative study guided by Aycan’s (2008) cultural perspective on work–family experiences (see Figure 1), which conceptualizes WFC as shaped by culturally embedded values, role expectations, and social norms, aims to explore how Chinese employees interpret and manage work–family conflict in their daily lives. Within this framework, culture influences both the perceived importance of work and family roles and the strategies individuals use to navigate competing demands. Drawing on Aycan’s (2008) model, this study qualitatively explores how Chinese cultural values, such as filial piety, family collectivism, and obligations, shape individuals’ understandings of WFC, shedding light on the potential culturally relevant mechanisms underlying WFC in China.

Theoretically, exploring how WFC is interpreted and negotiated in China can enrich existing work–family models by highlighting culturally situated meanings and relational expectations that have been largely underrepresented. Practically, deeper insight into how Chinese employees experience WFC can support the development of workplace and policy interventions that are responsive to local cultural norms, collective family practices, and informal support structures.

### Literature Review

The COVID-19 pandemic brought significant changes to daily work and family life, with remote working, school closures, and increased domestic responsibilities making it more difficult to keep work and family roles separate (Vitoria et al., 2022). The merging of work and home environments blurs the work–family boundary and intensifies the likelihood of WFC and its negative impacts, such as psychological distress, burnout, and reduced physical health on employees (e.g., Borgmann et al., 2019; Giancaspro et al., 2023).

Traditionally, society assigned males to be the family’s main breadwinners and only focused on the work role, whereas females were the housekeepers and only focused on the family role (Korabik et al., 2008). As society develops, the increased number of dual-career couples, single parents, and female employees creates a conflict between traditional gender roles and workplace structure (Korabik et al., 2008). The extra role demands for both genders, such as the wife having to go to work to share the family financial burden and handle domestic chores, and the husband having to be involved in housekeeping instead of only being the family’s main breadwinner, created tension between work and family roles and led to WFC (Lu & Cooper, 2015). Recent research has shown that these shifting expectations can result in difficulties in coordinating responsibilities within the household, often making the balance between work and family more challenging for both partners (e.g., Favez et al., 2023; Gemmano et al., 2023; Balenzano et al., 2025).

These shifting role expectations, however, do not necessarily replace traditional gender norms. Women often continue to take on the majority of childcare and housework even when working full-time, and this unequal division of unpaid labour remains strongly tied to traditional gender role attitudes (Yucel & Chung, 2023). As a result, women may experience greater strain as they attempt to manage both paid and domestic responsibilities (Roskam & Mikolajczak, 2020). Meanwhile, men are increasingly expected to be more involved in family life without a reduction in their breadwinning role, leading to additional pressure in balancing work and family demands (Stertz & Wiese, 2024). The above examples suggested that WFC is shaped not only by the amount of work and care involved, but also by enduring cultural beliefs about work and family roles.

These cultural beliefs influence how individuals make sense of their responsibilities in work and family life. In the Chinese context, fulfilling one’s work and family roles is often understood as contributing to the wellbeing and stability of the family unit (S. Chen et al., 2023). Aycan (2008) suggests that cultural values and norms play a central role in shaping expectations regarding appropriate behaviour in work and family domains, which in turn influences how individuals interpret and respond to WFC. 

These culturally shaped expectations influence not only how individuals evaluate work–family demands, but also the outcomes of and ways of coping with WFC. For example, Galovan et al. (2010) conducted a comparison study and found that Work-to-Family Conflict (WIF) was negatively related to job satisfaction in the American sample but unrelated in the Singapore sample, whereas S. Chen et al. (2023) found that WFC was positively associated with life satisfaction among Chinese employees, since fulfilling both work and family responsibilities was viewed as an expression of commitment to the family. Additionally, Sirgy et al. (2020) concluded that individuals may engage in coping strategies such as prioritising tasks or seeking support to maintain a workable balance between domains; however, the selection and perceived effectiveness of these coping strategies are shaped by cultural beliefs about the meaning of the stressful events and situations (Ji et al., 2022).

Furthermore, recent qualitative studies have shown that these cultural meanings play a central role in how individuals describe and respond to WFC. McMullan et al. (2018) and Namdari et al. (2019) demonstrated that people often articulate WFC through narratives of responsibility, sacrifice, and moral duty, indicating that the emotional significance of family roles shapes how conflict is understood and justified.

The above studies provide strong evidence that WFC is subjective and shaped by societal and cultural contexts, influencing how individuals interpret and respond to competing work and family demands (e.g., Kossek et al., 2011; S. Chen et al., 2023; Galovan et al., 2010; Allen et al., 2020). However, existing reviews indicate that the development of WFC research has been strongly grounded in Western contexts. For instance, a recent literature review of the family side of WFC found that the majority of studies focused on spousal and parenting roles within nuclear-family households, with comparatively fewer studies considering extended caregiving responsibilities (Reimann et al., 2022). Similarly, cross-cultural review work has shown that WFC frameworks and measures have predominantly been shaped by Western assumptions about family structure and caregiving roles (Shockley et al., 2017). Although Western research does address eldercare and the “sandwich generation,” these topics tend to exist as smaller, specialised sub-literatures rather than central domains of WFC research (e.g., Lam et al., 2022; Neubert et al., 2021). In contrast, intergenerational caregiving and kinship-based support are widely embedded in Chinese family life (Lu & Cooper, 2015), suggesting that WFC may arise from a broader set of expectations. Research grounded primarily in Western nuclear-family models may therefore not fully capture the contexts through which WFC is experienced in China.

To address this gap, this study aims to explore WFC and its related factors in China. It is hoped to provide research directions for future Chinese studies and help improve future studies’ contextual relevance and the practicality of the findings. To explore the WFC in China, the research questions that guide this study are as follows: 1. What have Chinese individuals experienced in terms of WFC? 2. What perceived contexts or situations do individuals in China see as typically influencing or affecting experiences of WFC?

## 2. Materials and Methods

### 2.1. Study Design

This qualitative semi-structured online interview study was guided by Creswell’s (2013) phenomenological approach. Qualitative interviews can explore a phenomenon of interest and allow a thorough understanding of the phenomenon (Opdenakker, 2006). Semi-structured online interviews via WeChat (Tencent, Shenzhen, China) (a Chinese-developed app similar to WhatsApp) were adopted in this study. The exact app version(s) were not recorded, and participants used their own installed versions; therefore, platform versions were not controlled. The main reason for choosing online interviews was due to the COVID-19 outbreak; all interviews were conducted during the beginning of the COVID-19 outbreak from August 2020 to October 2020, and no face-to-face research was allowed (British Psychological Society, 2020). We adopted descriptive phenomenology following Creswell (2013), beginning with participants’ lived experience and bracketing prior explanatory theory during analysis. Aycan’s culture–WFC model was used only to contextualise the phenomenon; no theoretical constructs informed code generation or theme development.

Moreover, because this study focused on exploring the lived experience of WFC, Creswell’s (2013) phenomenological method (Creswell, 2013) was used in this study. Creswell’s (2013) phenomenological method is closer to descriptive phenomenology, which is defined as phenomenological research that describes what a particular lived experience of a phenomenon or concept means to a group of people. Such a method explores the participant’s shared experiences of a phenomenon (Creswell, 2013). In addition, this phenomenological method explores the phenomenon of interest from two general questions: “What have you experienced in terms of the phenomenon? What contexts or situations have typically influenced or affected your experiences of the phenomenon?” (p. 61). Because Creswell’s (2013) phenomenological method broadly explores and describes the common contexts (factors) that affect participants’ experience of a phenomenon, it fits well with the research questions of this study.

### 2.2. Participants

Recruitment yielded a purposive convenience sample with one snowball referral (P5 referred by P8). This may bias the sample toward digitally connected urban employees comfortable discussing family roles; transferability is therefore limited to similar contexts. A total of 16 participants (9 males and 7 females) voluntarily participated in this study without receiving any form of compensation. All the participants in this study have met the following inclusion criteria (a) over 18 years old, (b) Chinese nationality and living in Mainland China (grew up in China), (c) Full-time employed, (d) Have a family (spouse/partner and/or children living at home) or living with parents, (e) Have experienced the conflict between work and family. Additionally, all participants were independent of the researchers and had no prior relationship with them. The average age of the participants was 34 years old, with the youngest participant being 24 years old and the oldest participant being 53 years old. Ten participants are married, one is divorced, two participants are in a relationship, and three are single. Moreover, nine participants have at least one child in this study. Due to the anonymity of the present study, the 16 participants were coded P1 (the first participant) to P16 (the 16th participant), respectively (see Table 1).

### 2.3. Procedure

After receiving the ethics approval, the researcher posted the recruitment letter on the mainstream social media platforms in China (i.e., WeChat, Weibo [Tencent, Shenzhen, China], Zhihu [Zhihu, Beijing, China], and QQ [Tencent, Shenzhen, China]). Participants accessed these platforms using their own installed app versions; therefore, platform versions were not controlled. People who met the inclusion criteria and were interested in participating contacted the researcher directly via WeChat, Weibo, Zhihu, QQ, or email. Eligibility was verified before scheduling interviews. All interviews were conducted during a two-month period from August 2020 to October 2020. During this two-month period, the border of China was restricted, and the “FIVE ONES” control policy (e.g., each airline was only allowed to have one flight per week to leave/enter China) was implemented (Civil Aviation Administration of China, 2020), which limited the spread of COVID-19 in China and thus no lockdown restriction was applied. Nevertheless, face coverings, daily COVID-19 testing, and Green Health QR Code were implemented during this two-month period, which might have affected the lifestyle and working life of the interviewees to some degree (Lin et al., 2021).

An information sheet was provided once the participants showed interest in participating in this study. An electronic consent form was signed and returned to the researcher before the interview. The researcher asked about the participant’s willingness to participate in this study again at the beginning of the interview. Background information of the interviewee was asked at the beginning of each interview. All the interviews lasted between 30 and 60 minutes. The length of the interview depended on the living status of the interviewee. For example, an interviewee with no child might have never experienced and responded less to child-related WFC; therefore, the interview was shorter. Questions related to this study’s research questions were asked during the interviews. One thing worth noting is that, despite all interviews being conducted during the COVID-19 pandemic, the interview questions were not framed in terms of a specific time period (e.g., pre-pandemic or during the pandemic). At the end of the interviews, the researcher asked the participants to recommend someone who met the inclusion criteria. Only one participant (i.e., P8) referred another interviewee (i.e., P15) to participate in this study. A debriefing form was followed once the interviews were finished. All the interviews were audio-recorded using Piezo (Rogue Amoeba Software, Boston, MA, USA, version 1.6.5). The interview schedule is as follows (see Table 2).

### 2.4. Ethics

This study has received ethics approval from De Montfort University (Ref: 3609). To protect the confidentiality and anonymity of the participants, all information that could identify them was removed from the transcripts. An identification code was given to each participant (e.g., participant one was referred to as P1) and used in this study for quotation identification. All electronic consent forms were stored on the University’s encrypted OneDrive system, accessible only to the first author (SC) in accordance with the approved ethics application and De Montfort University data-protection policy. Additionally, participants were informed that they had the right to withdraw from the study before, during, and up to 14 days after the interview.

### 2.5. Language and Translation

Language is the fundamental tool in qualitative research because it allows the interviewer and interviewee to interact and also affect the interpretation of the results (Hennink, 2008). The most common difficulty during cross-cultural studies is that the researcher cannot speak the native language (Liamputtong, 2010). In this qualitative study, all interviews were conducted in Chinese. The first author (SC) can speak both Cantonese and Mandarin fluently; thus, the participants can choose to speak Cantonese or Mandarin, whichever makes them feel comfortable. In general, a total of 11 participants spoke Cantonese and 5 participants spoke Mandarin during interviews.

This study adopted Shavarini’s (2006) translation strategy, which only translates the significant statements that are used as quotations in writing. Data were analysed in the original language (both Cantonese and Mandarin). Analysing the data in the original language helps to minimise the interpreting bias and the change in the meaning of findings during translation, because some concepts/words cannot be properly translated between languages (e.g., Hennink, 2008; Liamputtong, 2010). After analysing the data, the significant statements that would be used in writing were translated into English by the first author (SC). The translated statements were then proofread and back-translated by the first author (MC), who speaks both Chinese and English fluently and specialises in occupational psychology. Not using a professional translator or interpreter is first because working with a professional translator or interpreter might be less efficient if the researcher him/herself is a bilingual (bicultural) researcher who is more familiar with the topic of interest (Ryen, 2002); second, Kaufert and Putsch (1997) argued that a translator or interpreter is “a gatekeeper who has the power to elicit, clarify, translate, omit, or distort messages” (p. 72), which might interference the findings.

Although most key concepts had established English equivalents, such as “孝” (filial piety), “家庭责任” (family responsibility), and “工作与家庭冲突” (work–family conflict), the translation process was carefully reviewed to ensure that cultural meanings were preserved. When a Chinese expression carried implicit meanings, for instance, filial piety implying moral duty and social obligation, clarifying notes were added in brackets to maintain the intended sense. The bilingual and multicultural author team (Chinese, Taiwanese, and European researchers) cross-checked all translated excerpts to ensure conceptual equivalence and reduce the risk of confirmation bias through shared reflexive discussion.

### 2.6. Data Analysis

In this study, all transcripts, significant statements, and meaning units were managed and coded using Microsoft Word, which supported the systematic, iterative engagement with the data central to Creswell’s (2013) phenomenological method. Following Creswell’s (2013) phenomenology analysis strategy, we first ensured that the phenomenological method was appropriate for examining the research problems. Second, we identified the phenomenon of interest. Third, data were collected from people who have experienced the phenomenon of interest. Fourth, the participants were asked the two general questions: “What have you experienced in terms of the phenomenon? What contexts or situations have typically influenced or affected your experiences of the phenomenon?” (Creswell, 2013, p. 61), and other open-ended questions that relate to these two questions. Fifth, we analysed the data, highlighted significant statements, formulated meanings, and clustered these meanings into themes. Sixth, we described the themes. Last, we discussed the essence (common meaning) of the phenomenon as reflected in the themes.

In this study, the transcripts of all interviews were created and initially analysed by the first author (SC). Information relevant to the interview questions and how the interviewees experienced the WFC was condensed into key statements. The next step was “horizontalisation”, which removed the overlapping statements from the list of significant statements (Creswell, 2013). After horizontalisation, the first author (SC) interpreted the meanings of significant statements and formulated meanings that were clustered into themes. The coded statements and emerging themes were then reviewed and discussed collaboratively with the second first author (MC), and the final thematic structure was agreed upon by all authors.

Meaning saturation was monitored throughout the analytic process to ensure sufficient depth and completeness (Hennink et al., 2017). Saturation was judged to be reached when further interviews no longer generated new significant statements, new variations in formulated meanings, or changes to the developing thematic structure. By the twelfth interview, core meaning units (such as prioritizing family over work, emotional spillover from work into the home, and pressure associated with filial obligation) were reappearing without introducing new experiential variation. The remaining four interviews confirmed these established patterns and did not extend the thematic range. Data collection was therefore concluded once redundancy in meanings and themes was observed.

The present study followed Creswell’s (2013) descriptive phenomenological sequence: horizontalisation of significant statements, formulation of meanings, clustering of meanings into themes, development of textural and structural descriptions, and synthesis of the invariant essence. The subsequent theoretical discussion is therefore grounded in, rather than imposed upon, the empirical descriptions of participants’ lived experiences.

To strengthen analytic rigor, a code–recode procedure was conducted after a two-week interval to check the stability of code application. Any discrepancies were discussed until a consensus was achieved. Peer debriefing sessions were held within the author group to critically examine coding decisions, theme boundaries, and interpretations. To enhance transparency, each theme and subtheme was defined by clear analytic boundaries, which are outlined in the “Definition” column of the codebook (Table S1). A theme tree (Figure S1) illustrates the hierarchical structure of themes and subthemes, a detailed thematic chart (Table S2) provides exemplar quotations from each participant for every theme and subtheme, and an audit-trail snapshot (Table S3) summarises key methodological decisions. All of these are provided in the Supplementary Material.

### 2.7. Trustworthiness

The trustworthiness of this study was supported through adherence to Creswell’s (2013) phenomenological procedures, systematic reflexivity, transparent documentation of the analytic process, and collaborative review among authors. Member checking was not used. Returning transcripts for participant verification may prompt retrospective reinterpretation rather than the original lived descriptions, which is inconsistent with phenomenological reduction (e.g., Giorgi, 2006; Smith et al., 2021). In addition, the trustworthiness of this study was ensured using the criteria proposed by Lincoln and Guba (1985), including credibility, dependability, confirmability, and transferability. During data collection and analysis, both first authors kept reflexive notes to record thoughts, decisions, and reflections on their roles in the study. These notes helped the authors remain aware of their own assumptions and reflect critically on how their perspectives might shape their interpretation. To enhance credibility, brief summaries of the main themes were shared with two participants, who confirmed that the findings reflected their experiences. Accounts that did not align with the main patterns were also examined carefully and integrated into the analysis where relevant. Dependability and confirmability were supported through detailed documentation of analytic decisions and regular discussions within the author group to review how themes had been developed and refined. The use of direct participant quotations in the Results section illustrates how interpretations were grounded in the data. Transferability was supported by providing thick contextual descriptions of participants’ demographic characteristics, work and family contexts, and cultural settings, allowing readers to assess the applicability of the findings to other populations or situations.

### 2.8. Rigour, Reflexivity, and Researcher Positionality

To ensure rigour, we engaged in systematic reflexive practices throughout data collection and analysis. Among the two first authors, Chen conducted all interviews and is a bilingual (Cantonese, Mandarin) and bicultural researcher, originally from China and educated within the UK academic system. This dual insider–outsider position facilitated rapport and a nuanced understanding of participants’ experiences, while interpretations were regularly discussed with the co-authors to monitor potential bias and maintain consistency with the data. Both first authors kept reflexive journals to record analytic decisions and prior assumptions drawn from the literature and Aycan’s (2008) cultural framework, which served only as a sensitising background. This process helped identify points where participants’ accounts diverged from our expectations, most notably in Theme 1 (“That is life”), where work–family conflict was described as normalised rather than stressful. The findings apply to sixteen full-time, social-media-using Chinese employees recruited through purposive convenience sampling, with data collected between August and October 2020 during the early COVID-19 outbreak and the national “Five Ones” control policy. Reflexivity was maintained through continuous self-evaluation of how our positions and assumptions might have shaped data interpretation, and we remained attentive to keeping the emerging meanings grounded in participants’ lived experiences.

## 3. Results

From 16 transcripts, a total of 297 significant statements were found. The examples of significant statements and their formulated meanings are listed below (see Table 3).

The formulated meanings were then clustered into 6 Themes and 17 sub-themes (see Table 4).

***Theme 1: Normalised Conflict.*** Focusing on the attitudes toward WFC, most participants mentioned that the conflict between work demands and family needs is just a “part of life” and it is a “life experience” that everyone has to go through. For example, participant 4 (P4) said, “all of these [different types of WFC] are life experiences, […] it is just a very common phenomenon, I think the experience of conflict [between work and family] has actually become normalised.” On the contrary, some participants think that WFC is considered a problem that affects their lives; however, compared to other difficulties in life, it was just a minor problem that is not worth mentioning. P16 stated, “It is a problem, just not a big deal. I mean, a simple example, your work and family are actually like a scale, it is not possible to balance it easily.”

It appeared that because of such a standpoint toward WFC (i.e., just a life experience or just a minor problem in life), most of the participants tended to *turn a blind eye* when the WFC happened. P8 remarked, “Life goes on. So, you cry about it, then just keep moving forward.” And P1 also reported as follows:

*Just don’t think about it and ignore it; it is common in every household. Maybe everyone will deal with it differently, but for me, I would just act as if nothing happened. Give it a couple of days, and everything will be okay again.*

***Theme 2: Cultural Role Norms.*** In this theme, interviewees described the Chinese culture and beliefs associated with WFC. All the participants showed a very strong sense of family and thought that *work is for the family*. A strong sense of family was evident during P6’s interview; he stated,

*I think if you have to stop your work because you have to provide care for your family members, I actually don’t think it is a conflict; it is just a thing that you must do for your family, it is a responsibility.*

Similar responses were found during the interview with P11; he said, “Actually, things like my job are not that important if you compare them to my family. It is not even worth mentioning.” And during P3’s interview, she stated, “You can find another job, but you only have one family, so their health or other family issues are definitely more important.”

*Work is for the family* was endorsed by most of the participants. Work was described as a way to make money so that they can provide a better life for their family. P12 said, “It is a good-paying job. So even though [being a nurse] is very stressful […] still, I am willing to sacrifice because the income can give my child, or the family a better future.”

Additionally, Chinese families seem to have a more supportive and *united relationship*, which might ease the WFC experience from different aspects. For example, elderly parents would take care of their adult children’s or grandchildren’s daily lives and/or financially support their adult children. For example, P7, who is living with his son and husband, said,

*When I need to buy a house [parents will give us money]; […] or if I have a business trip, or my husband has a business trip and I have to work, then they (parents) will come to help us [to take care of the child].*

P4, who is 48 years old and living with his wife and two children, stated, “They (elderly parents) will cook for us and take care of the children for us.” When the researcher asked, “What is your thought about your parents still helping you out instead of you taking care of them?” later during the interview, he stated, “Nothing, it is good. I mean, that is the case in our Chinese families. Parents are always the providers; I think it is just a common phenomenon.”

Except for the help from elderly parents, Chinese males seem to enjoy support from female spouses, but such support seems to be one-sided. “She understands I have to work” and “She understands I am tired” were mentioned by some married male participants during the interviews. However, P12, who is a married female participant, said, “Because my husband does nothing [at home], I mean my husband, he can’t share [the housework] with me, so I feel like I have more things to do.” In other words, the male seems to receive more support and understanding from his wife, which might ease the WFC; however, on the other side, the female appears to have more WFC because of family responsibility.

It seems that such one-sided support was due to the influence of the *traditional gender role*; the inequality in gender roles was endorsed by most of the participants, especially among the married participants or the participants who were still living with their parents. Participants described this gender role as part of the “society”, “culture”, and “tradition”. P8, a woman with two children, said, “the society, I mean even right now, […], people still think that women should stay at home and be the caregiver; this [traditional] thought is deeply ingrained in belief.” And P14, a husband with two children, said, “the domestic chore should be done by the wife, that’s just part of our culture.”

It appeared that women would have more family responsibilities, such as providing care for the children and taking on more housework. P2, who lives with his parents and girlfriend, stated, “[housework] basically is done by my mum and my girlfriend.” P5, who is married and still living with his elderly parents, also said, “In general, my mum does most of it (housework), then my wife will finish the rest, mainly take care of the child’s daily lives.”

Subsequently, men and women seem to get used to this gender role, which leads to women having more family responsibilities and being more willing to take on more family responsibilities, thereby letting their husbands or adult children focus on the work domain. For example, P1 stated, “Sometimes I got off work very late, around 1 AM or 2 AM, she (my wife) finished all the housework, she didn’t leave any things for me to do.” And P7, who is married and has a son named N, said, “I am quite busy [at work], then my husband hopes that I could spend more time at home to help N with his homework, but I might…, so I have to take some time off [from work].” And when the researcher asked her how she felt about having more family responsibilities later during the interview, she stated,

*I feel like it is just part of my life, I already get used to it […] it is always me who takes care of his (the son’s) daily life […] I, I don’t seem to feel anything anymore, I just feel like it is just my responsibility.*

As a sub-theme of the cultural role norms, *filial piety* was endorsed by all participants in this study. All the participants believed that taking care of elderly parents is a way to fulfil their obligations as children. As P12 stated, “you, as the child, must take care of them (parents), not just financially support them, but also emotionally care about them.” Although most of the participants’ parents in this study are still relatively young and in good health, the thought of having to take care of their parents when they are older has negatively affected most of the participants to some degree. P11 stated,

*[Having to take care of parents] is pressure, because right now, it’s not like I am doing very well at work, still not achieving my [occupational] goal; I mean, I am not rich enough, but my parents are getting older and older, so I just feel so much pressure.*

P6 explained the reason why the thought of filial piety impacted him and what he was worried about; he said,

*As they (elderly parents) get older, they may start to have some health issues; then the first [thing I am worried about] is the medical treatment [needs money], second is I have to provide care, which means I have to take some time off from work, and then my income will be affected.*

***Theme 3: Family-Driven Interference.*** In this cluster, participants described the family-related factors/experiences associated with FIW. It appeared that due to the strong sense of family, most participants claimed that the only reason they would stop their work was the *health of family members*. Some participants said the only thing that stopped their work was their children’s health; for example, P1, who has a 2-year-old daughter, said, “My child was suddenly sick, then I [left my work and] went back home.” P7, who has a 12-year-old son, stated, “If he (my son) feels sick, I will definitely stop everything at work and take him to the hospital.” Some said the health of their elderly parents had stopped their work. For example, P6 stated, “My parents were both ill a while ago; I stopped my job to take care of them.” And P14 stated, “if parents are sick, then I will stop everything I am doing at work […] that’s it, that’s nothing to talk about, I can lose my job, but I can’t lose my parents.”

It appeared that due to the influence of societal/cultural norms, such as “I must take care of elderly parents by myself”, *being the only child* would exacerbate the negative impact of filial piety on FIW. Most participants often mentioned “because I am the only child” during the question regarding taking care of elderly parents. For example, P4 stated, “Because I am the only child, will I have the ability to take care of my parents by myself when they are old? [Every time I thought about that] I started to panic, and I started to worry.” And P12 said, “If you are an only child, you probably need to take care of 8 elderly parents. Your parents, your grandparents, your spouse’s parents, and your spouse’s grandparents.” It seems that the lack of sibling support might be the reason why being the only child would exacerbate FIW. This is evident in P16’s interview:

*It’s very realistic; you have to deal with it yourself, or you have a sibling to handle it all together. It won’t be the same. I mean, at least, you won’t be so stressed [if you have a sibling]. If you have many siblings, things may be much easier; at least you can discuss [with your sibling] when something happens.*

As a sub-theme of the Family-Driven Interference, *having children* was endorsed by all the parent participants. All the parent participants in this study claimed that as their children become older, they have more stress in both work and family domains due to *parental expectations*. P1, who has a 2-year-old daughter, said, “Because you have to know that the kindergarten charges at least 1000 RMB per month, that’s an extra pressure, so I have to walk the extra mile.” Also, P7, who signed up for 3–4 after-school classes for her son to improve his school grade, reported as follows:

*First, I have to take him to and from the after-school classes, so it costs my time; then it makes me a little bit anxious because I have to make sure he absorbs the knowledge, right? So, I have to make sure he does the homework, and I have to give him other assignments to improve his weaknesses. I have to examine his work, like discuss it with him and communicate with his teacher after class. All of these are like invisible pressure.*

And P12, who has two six-years-old daughters, stated,

*I work like 10 h, and then I finally get back home […] I barely have time to enjoy my dinner, my body and my mind are not ready yet, and then I have to help with my children’s homework; I just feel like I am a bit out of my depth.*

The parental expectation was also linked with “good job” and “contribute to society” by most of the parent participants. For example, P7, who has a 12-year-old son, said, “I hope he can find a good but not stressful job, and of course, it is a good-paying job too. So that he can take care of himself and his own family.” And P14, who has two children, said, “I hope they can get into a good university; I hope they can have good grades in school, be useful, and contribute to society.”

***Theme 4: Work-Driven Interference.*** In this theme, people described the relevant work-related factors affecting WIF. Because the job was viewed as a tool to improve family lives by most of the participants, a high level of work demands seemed to be more acceptable. However, it appeared that everything comes with a price, and work-related factors still harm participants’ family life. “Definitely” was often used by the participants when the researcher asked, “Does your job affect your family life?” For example, P13 said, “It (work) definitely impacted my family life to some degree; if you have a job, plus, you have family, then you definitely will be facing some problems.”

A sub-theme of Work-Driven Interference, *money is the cure* was endorsed by most of the participants. It appeared that income from work had a strong influence on the level of WFC. For example, P1, the main breadwinner of his family, stated, “Will it (WFC) get worse? It really depends on the money. To be honest, the biggest problem in my family right now is the money.” And P5 stated, “In my family, if I can really solve most of the financial problems, then I think 70,80 per cent [of WFC] will be fixed.”

As a sub-theme of Work-Driven Interference, the *occupational difference* was endorsed by most of the participants. It appeared that people in different occupations would experience different WFC and influence the level of WIF. For example, P7, who is a university English lecturer, said,

*My job is relatively simple, [I] don’t have to go to university every day; you only be there when you have lectures, and you can leave after finishing the lecture; you don’t even need to be in contact with other colleagues […] when you don’t have to be in contact with others, you have fewer conflicts [at work].*

It appeared that the working hours had affected the experience and the level of WIF. Some participants claimed that *long working hours* at work increased the incompatibility between work demands and family needs. For example, P3, who works from 9 AM to 9 PM, six working days per week, said,

*My grandfather is sick and has to stay in the hospital right now. My mom is having a rough day; she really wants someone to take turns to take care of [my] grandfather with her, but [I] don’t have the time to do so.*

P10, who owns a restaurant and has to work from 8 AM to 9 PM six days per week and also have to spend a half-day on Sunday at work, stated, “so it is tough for me to fulfil my family responsibilities; it is just impossible to do both, there is only so much time […], so it is impossible for me to handle the things at my family.”

In addition, the influence of *flexible working hours* on WIF was endorsed by some of the participants. Some participants claimed that flexible working hours have decreased the incompatibility between work demands and family needs. P13 said, “Because the work time of my job is quite flexible, it gives me more free time with my family.” Contrarily, some participants claimed that the inflexible working hours at work have increased their WIF. For example, P12, who is a nurse and follows a shift work schedule, stated,

*I mean, sometimes, my children’s school would arrange things like parent-children activities, parent-teacher conferences, or sports meets, and I, as the parent, am required to attend. But because of my work shift, it is hard for me to change my shift and go to these events that I am supposed to attend.*

Moreover, it appeared that a *supportive work environment* could help to ease the negative experience at work, hence decreasing WIF. For example, P13, who entered the workplace one year ago, said,

*The heads of my department really care about you, helped me a lot, […] just like a master’s supervisor or PhD’s supervisor, they are like the supervisors, guide you and teach you step by step […] because of these bits of help, it made my work easier.*

On the contrary, an unsupportive environment would make the participant feel more work distress, ultimately leading to or increasing the level of WIF. For example, P4, who was recently promoted at work, reported as follows:

*My team leader said he would take care of me; I mean he promised to guide me, but he had his own work too. So, most of the time, he just gave tasks to me; I had to figure them out by myself; I was always scared that I would mess it up because lots of the tasks were new to me, […] so I felt anxious all the time.*

***Theme 5: Affective Spillover.*** This theme focuses on the psychological/emotional aspects that are related to WFC. A sense of *work distress* was evident in all the participants. It appeared that work distress had led to the experience of WIF. Some participants described this work distress as the “emotion at work”. For example, P6 said, “I keep telling myself don’t bring back the emotion at work to my family. I mean, like the bad emotion at work, but I am just a human, so I couldn’t just let it stop.” On the other hand, work distress is also described as a “physical exhaustion”. Sentences like “I am so tired at work.” “My work makes me so tired,” And “I don’t want to do anything when I go back home from work.” were often said by the participants.

As a sub-theme of the Affective Spillover, the *display of anger* was endorsed by some of the participants. It appeared that some participants, especially the young, unmarried participants, would tend to lose their temper at home easily and vent on their elderly parents, if they were overloaded at work or had ill feelings at work. “I get angry for no reason [at home]” or “I will get mad easily [at home]” were mentioned by some participants. In addition, it appeared that the female participants who are married and have children tended to vent to their children when they were overloaded in both work and family domains. For example, in P12’s statement, she said,

*Sometimes I had a long day and was very tired. Then, when I got back home, I had to help with my children’s homework, […] sometimes I cursed, not cursed, I mean I yelled [at my children], [I would say:] ‘how could you still not understand? I have taught you so many times!’ with a tone of blame. […] I became angry easily when they didn’t know how to do their homework.*

And P7, who has a 12-year-old son named N, said, “I became angry, I was mad, mad at N because I would think that he’s all grown up; why couldn’t he manage himself? I mean, why couldn’t he manage his time by himself?”

Additionally, the feeling of being an incompetent family member and the inappropriate behaviours toward family members have caused some participants to have *guilty feelings*. It appeared that some participants would feel guilty when they could not fulfil their family responsibilities due to work demands. For example, P10, who is living with her big sister, daughter, and mother and works over 66 h per week, stated,

*My child is still young; I wish that I could have more time to be with her; everyone would want to spend more time with their kid; but I just couldn’t do that [because of the work], […] [although] I asked my big sister to help me [to take care of my child], but you have to understand, this responsibility, I couldn’t just throw it all to my sister, right? After all, she is my baby girl!*

And P15, whose parents are left-behind elderly, stated,

*I feel guilty. I didn’t fulfil the obligation of being their child. All I did was give them money [when I went back to see them], and it was not even a lot of money, so I feel a little bit guilty.*

Some participants felt guilty after venting on their family members due to the experience of WFC. For example, P11 mentioned that he feels guilty after he vents on his family members because of the stress at work; he said, “I actually feel very guilty [after I vent on my family members], but I couldn’t control it.” And a sense of guilt was also evident in P12’s statement; when the researcher asked her, “How do you feel about venting on your children because you are tired at work?” she responded,

*I thought about it afterwards, and then I realised I shouldn’t act like that, […] it’s my fault, I should be gentler, no matter how tired I am, I should adjust my emotions before I help with their (the children) homework.*

***Theme 6: Cyclical Strain.*** This theme focuses on the outcomes of WFC. Most of the participants in this study claimed that WFC had negative impacts on both their work domain and family lives. WFC is like a *vicious cycle* affecting participants’ work and family domains. This was evident in P4’s interview; she reported as follows:

*There was a period of time when I had lots of tasks [at work]. I slept very late, I became moody, […] it is like a vicious cycle, I was in a bad mood because of the stress at work, then because of my mood, I didn’t want to eat or do other things [at home], then because of that, I was in bad health, then I was sick.*

A similar explanation was provided by P12; she stated,

I [usually] get back home [from work] around 8 PM, […] I only have half an hour for my dinner, and I feel like my time is very tight every day. [After my dinner], then I have to spend some time with my kids and help with their homework. Usually, children should go to bed before 10 PM, but my kids won’t go to bed until 11 PM, […], so I couldn’t go to bed until at least midnight, […] if you don’t have enough sleep, you don’t have enough energy [at work].

Some participants claimed that they had to *deny opportunities* at work to fulfil their family needs. For example, P2, who is a small business owner and lives with his girlfriend and parents, said,

*My girlfriend doesn’t want to live at my home with my parents when I am not there. So, let’s say I have a business trip; then I must go back home on the same day […] [can’t go on a business trip that takes more than one day] is not just a little bit depressing; it’s more like frustration and what a shame [I lost a potential business opportunity because I can’t go on the business trip].*

In addition, P8, who is a single mom with two children, claimed that the conflict between family needs and work domain has caused her to lose the development opportunity at work; she said,

*After you left your work [because of family needs] for a while, your company would not treat you the same [when you come back]. I mean, during the period of your leave, lots of opportunities they given to other people.*

Last, most of the participants in this study claimed that the WFC has made them *become lazy*. It appeared that the overwhelming workload or the emotion at work/family affected the emotions and behaviours of the participant in another domain. For example, “Don’t want to do housework” was often mentioned by the married male participants. P1 said, “When I came back home [after work], I was so tired, I didn’t want to do anything.” In addition, P10, who is the main breadwinner of her family, said, “I will be affected by their (family members’) emotions; […] I would feel very depressed all day [if my family members told me they are in a bad mood], and it would affect my work performance.”

## 4. Discussion

The present study explored the WFC phenomenon in China from the standpoint of Aycan’s (2008) cross-cultural perspective. The findings were consistent with Aycan’s (2008) cultural perspective; WFC is a relatively subjective phenomenon, and that culture would influence work and family-related support and demand, thereby affecting the experience of WFC (e.g., Lu & Cooper, 2015; Korabik et al., 2008; Shaffer et al., 2011). Some of the findings are similar to the previous Western findings (e.g., Choo et al., 2016; Innstrand et al., 2010; Roth & David, 2009; Cerrato & Cifre, 2018; Bakker et al., 2008; Korabik, 2015). I.e., the influence of occupational differences (i.e., long working hours and flexible working hours, supportive work environment) and gender roles on WFC, the psychological and emotional response to WFC (i.e., distress, anger, and guilt), and the decrease in work/family performance due to WFC.

The findings of this study also offer a theoretical contribution by extending Aycan’s (2008) culture–work–family conflict framework. While Aycan highlights how cultural norms shape expectations across work and family domains, our analysis shows how these expectations operate through culturally embedded meaning structures that influence how Chinese employees interpret and respond to conflict. Mechanisms such as filial piety, parental academic pressure, the normalisation of self-sacrifice, and moral expectations surrounding family duty shaped participants’ definitions of conflict, their prioritisation of roles, and the acceptability of coping strategies such as endurance and avoidance. These culturally specific meaning structures illustrate that WFC is experienced not only as a negotiation of competing demands but also as a moral and relational obligation embedded in Confucian family ideology. By revealing these meaning-making processes, the study extends behavioural science by demonstrating that Western stress–strain models do not fully capture the moral, relational, and culturally grounded experience of WFC in non-Western contexts.

Building on these theoretical insights, the lived experiences of participants revealed how culturally embedded meanings shaped their interpretation of WFC. The participants in this study described WFC as just a life experience or just a minor problem in life. This standpoint toward WFC has led most of the participants to underrate the negative impact of both types of WFC. However, this attitude towards WFC did not simply mean that WFC has no negative impact. The negative impacts of WFC on the work domain (e.g., decreased work performance), family lives (e.g., becoming lazy) and an individual’s well-being (e.g., guilt) were reported by most of the participants. These insights are consistent with previous research (e.g., Borgmann et al., 2019; Giancaspro et al., 2023), which has shown that WFC is associated with heightened stress, reduced psychological wellbeing, and increased emotional fatigue.

Instead, such an attitude has limited participants’ coping strategies with WFC, resulting in most participants choosing to use an avoidance coping strategy to cope with WFC (e.g., P1 said, “[…] give it a couple of days, and everything will be fine again”). Such strategies may provide short-term relief, but research suggests that emotion-focused or avoidant coping does not effectively buffer the negative association between stressful events and wellbeing, such as work–family spillover and its negative impacts (e.g., Sirgy et al., 2020; Penley et al., 2002; Aldao et al., 2010). Subsequently, the negative effect of WFC did not weaken. Adopting an avoidance coping strategy might be due to the influence of Taoism, which emphasises that the concept of good and bad is subjective; hence, there is no requirement to take extreme measures in order to eradicate what is considered bad, since nature will restore the balance without the need for action (Cheng et al., 2010).

Based on the descriptions of the interviewees, several Chinese cultural values seemed to influence the experience of WFC in China. The family collectivism orientation in China, such as strongly valuing the family and the duty of providing care for the family members (Lu & Cooper, 2015), created a strong sense of family that influences the work value of the interviewees, resulting in interviewees believing that work is one way to improve family wealth, instead of personal achievement. Such a culturally rooted sense of family responsibility, combined with Taoist beliefs about balance and acceptance, might provide further explanation for S. Chen et al.’s (2023) finding—that WFC is positively related to life satisfaction in China—as nature is believed to restore balance, making conflict seem temporary, while the resulting benefit to the family is viewed as long-lasting.

In addition, such a strong sense of family also influenced the participants’ decision-making. Most interviewees would rather give up their jobs when experiencing work–family dilemmas. These findings are similar to those found in Gui and Koropeckyj-Cox’s (2016) study, which argued that Chinese family values are somewhat in conflict with the development of society. Despite the development of society, such as globalisation, which has created more opportunities for employees, family collectivism orientation creates a strong sense of family obligation, forcing employees to pay more attention to the family domain and hindering them from pursuing greater personal achievement.

The family collectivist orientation also seemed to be mixed with other Chinese traditional values and contributed to the close and supportive relationship between parent and child and between husband and wife.

The supportive relationship between parent and child was mutual. On the positive side, a help-seeking coping strategy was spotted being used by most of the interviewees, especially those with children. Interviewees claimed that their elderly parents would take care of their daily lives and pay for the daily expenses. In addition, those who have children often ask their elderly parents to take care of their children when they are at work. Such support from elderly parents not only decreased the participants’ time needed for family responsibilities but also reduced their cost of living and eased the worries about family life, consequently decreasing family distress and therefore, minimising the risk of experiencing FIW (e.g., Lu et al., 2006; Byron, 2005; Direnzo et al., 2011).

Similar patterns have been observed in other familistic cultural contexts, such as Southern Europe, where grandparents provide substantial informal childcare that often plays a more significant role than institutional support systems (Balenzano et al., 2025). In such contexts, intergenerational involvement is not merely a response to logistical childcare needs but is deeply embedded in collective understandings of family duty, emotional closeness, and shared responsibility (e.g., X. Zhang & Chen, 2023; Mansilla-Domínguez et al., 2024). This cultural orientation may explain why participants used help-seeking coping strategies and viewed grandparental support as natural.

On the negative side, the value of filial piety has created a sense of must to support and care for their elderly parents, which stressed the interviewees out. Such stressful feelings might contribute to strain-based conflict, thus creating FIW (S. Chen et al., 2023).

The participants often mentioned two factors regarding the stressful feeling of fulfilling *filial piety*: the time needed to provide care for their elderly parents and the worry about money. The time required to provide care for elderly parents might result in an unbalanced time allocation between family and work, thereby leading to FIW (e.g., Lu et al., 2006; Korabik et al., 2008). The worry about money can be seen as a lack of financial security. In other words, *filial piety* has caused the participants to worry about their financial ability to handle unexpected costs (e.g., subjective financial insecurity, such as worrying about medical expenses for elderly parents). Under the feeling of financial insecurity, people tend to feel like they have to focus on the work domain in order to increase their income, increasing the risk of experiencing WIF (Odle-Dusseau et al., 2018).

These findings suggest that intergenerational support operates through reciprocity norms that both relieve and generate work–family pressures. While grandparental assistance reduces day-to-day family demands and lowers FIW, the cultural expectation to repay this support through ongoing care and financial provision for elderly parents simultaneously increases both time- and strain-based interference with work. In this way, the same cultural value of familial obligation that facilitates help-seeking also intensifies feelings of responsibility and pressure, shaping how individuals prioritise family and work when demands compete.

We noticed that the relationship with elderly parents seemed to influence the work/family factors that related to WFC, resulting in a difference between the findings of this study and previous Western findings. This seemed to give rise to unique situations affecting WFC. First, elderly parents’ support seemed to moderate the relationship between the number of children and WFC among the interviewees. Previous Western findings suggested that younger children would require more time to care, thereby increasing the time needed for child-rearing, which leads to an increase in WFC (Huffman et al., 2013). In this study, it seemed that because elderly parents would provide care for their grandchild, parent participants generally reported that they feel more WFC as their children grow up due to the cost of education and the time needed for help with children’s homework, and the feeling of distress when the children’s school grade did not meet parental expectations.

Second, many Western studies on the relationship between income and WFC focused on how income affects the available strategies to cope with FIW. For example, people who have high incomes can hire a nanny to take care of their children, decreasing the time needed for family responsibilities, and thereby reducing time-related FIW (Ciabattari, 2007). In contrast, instead of associating income with coping strategies, most interviewees associated income level with the future, such as worrying about the future of the family, and being afraid that if they cannot increase their income level, the experience of WFC would be exacerbated. In other words, income seemed associated with the strain-based WFC among the participants. This seems to be because elderly parents would provide daily and financial support to the interviewees. But at the same time, the thought of filial piety has created a feeling of financial insecurity. Such insecurity increases family strain, thereby leading to strain-based WFC (e.g., Lawrence et al., 2013; McGinnity & Russell, 2013).

Third, it seemed that the belief in filial piety exacerbated the experience of WFC among the interviewees with no siblings (i.e., the only child of the family). Most participants claimed that because they are the only child of the family, they feel more pressure regarding their ability to take care of elderly parents in the future. Due to the Westernised concept of “family” focusing on the parents and adult children, there is a dearth of Western studies investigating how being an only child would affect the experience of WFC. Only one Chinese study (i.e., S. Chen & Cheng, 2024) that we are aware of discussed the experience of being an only child, explaining that it increases the risk of having family overload. For example, an only child might need to spend more time and energy on caregiving responsibilities (e.g., eldercare) due to the lack of sibling support, thereby increasing WFC.

Moreover, traditional Chinese thoughts have influenced the relationship between husband and wife; gender inequality in household chores and childcare responsibility is still distinct in current Chinese society. Although dual-earner couples are becoming increasingly common, women typically remain the primary caregivers and housekeepers, reflecting enduring patriarchal expectations and domestic responsibilities (e.g., Li & Chen, 2024; X. Chen, 2024). The traditional gender norms continue to shape the distribution of household and caregiving responsibilities within Chinese families. Based on interviewees’ descriptions, male participants generally reported a lower level of WFC, whereas female participants reported experiencing a greater WFC due to their higher level of family responsibilities. This finding of a gender difference in the experience of WFC is in line with findings from previous studies, which also identified greater WFC for females (e.g., Rehman & Roomi, 2012; El-Kassem, 2019).

This unequal distribution of family responsibilities appeared to interact with participants’ strong sense of family obligation, producing a one-sided supportive relationship in which women not only assumed more domestic work but also expressed greater willingness to do so. However, this imbalance makes it more difficult for women to meet workplace expectations of constant availability and uninterrupted focus on work (Li & Chen, 2024). As noted in recent research, this tension can translate into gendered career disadvantages, including reduced promotion opportunities, slower career progression, and heightened employment instability for women (e.g., Sun et al., 2025; J. Zhang et al., 2021). This may help explain why P8, a single mother, felt she had lost development opportunities as a result of her family responsibilities.

Furthermore, other traditional Chinese thoughts, such as honouring the family and making the family proud through personal achievement (Xu et al., 2005), seem to contribute to the strong parental expectations in China. Such an expectation was often associated with the children’s educational performance (e.g., good grades at school) and reflected increased demands on parents’ time. The parent participants reported that they had to spend extra hours (e.g., help with children’s homework) or money (e.g., after-school classes) to improve their children’s educational achievement. This finding helped to understand why interviewees would feel more WFC as their children grow up. The age of children appeared to be one of the factors contributing to the disparity in the relationship between WFC reported among the 16 interviewees in this study and the findings from previous Western research. Yet, more studies are needed to explore this little-studied issue.

## 5. Limitations and Future Study

This study has three limitations. First, the qualitative data of the present study were collected via online interview instead of a face-to-face interview, which can provide more information from the body language of the interviewee (Opdenakker, 2006). However, due to the outbreak of COVID-19 and the lockdown, a face-to-face interview was not an option, and an online interview was much safer for both the interviewer and interviewees. Second, the oldest participant (P15, 53 years old) in this study claimed that his age had changed his view on work (e.g., achievements, goals, etc.), and he now would not think about work when he is at home. And thereby, he is experiencing less WFC; in other words, age as a demographic variable might influence the level of WFC. However, due to the nature of phenomenology research (Creswell, 2013), some of the demographic variables (e.g., age) that might affect the experience of WFC were not considered in this study if they could not be clustered into a theme. Third, due to the nature of the qualitative study, the relationship between the WFC and its antecedent/consequence cannot be verified (Casares & White, 2018). However, the findings provided insight into how the Chinese culture has affected the experience of the WFC phenomenon among the sixteen participants. A quantitative study is encouraged to further verify the accuracy of the findings of the present study. Last, the absence of cross-cultural comparison means that the cultural factors, such as filial piety, parental expectations, and the normalisation of conflict (i.e., conflict is not an issue but a daily experience), cannot be assumed to hold in non-Confucian cultural contexts. Future research could therefore compare how employees from different cultural backgrounds experience and make sense of WFC, which would help clarify the extent to which these findings reflect cultural specificity versus universal processes.

## 6. Contribution to WFC Literature

The findings of this study have several contributions to the existing WFC literature. First, this study provides deep insight into how Chinese employees experience WFC and has broadened the understanding of the WFC phenomenon in the Chinese cultural setting. And to the researcher’s knowledge, the present study is one of the first to use a phenomenological approach to researching WFC in China.

Second, most previous WFC studies were conducted in Western countries; this study explored the WFC phenomenon in China and described how people with a traditional Chinese cultural background experienced WFC. Although most of the findings are in line with previous Western studies, some differences between the Western countries and China in the experience of WFC were found; in addition, the findings suggested the relevant factors (e.g., the sense of guilt, being the only child, parental expectation, filial piety) that related to WFC in China, which provide a direction for the future WFC studies in China and in other Confucian heritage countries in Asia.

Lastly, the contrasts between the present findings and those reported in Western work–family conflict research highlight the need for continued investigation of WFC across diverse cultural settings. These differences underscore the importance of exercising caution when applying Western-developed theories or interpretations in non-Western contexts, as they may not sufficiently account for culturally embedded norms, values, and family role expectations. At the same time, the findings encourage the development of work–family frameworks that better reflect cultural variations, rather than treating cultural context as an external or secondary factor.

### Implications of the Present Study

The findings of this study indicated that due to the increased prevalence of the experience of the WFC phenomenon in China, participants started to normalise the WFC phenomenon and ignore its negative consequences. Although participants generally received a certain degree of support from their elderly parents because of the influence of traditional Chinese culture, which has helped to ease the WFC. However, such support seemed to be only at a material and physical level. Wallace (2005) pointed out that emotional support can improve individuals’ well-being and might decrease the WFC’s negative psychological impact. Thus, in addition to seeking material or physical support from family members, individuals should also seek emotional support from family members and/or supervisor/co-worker through communication; on the other hand, family members and colleagues could show emotional support through listening to and empathising with the individual when they are experiencing distress events, such as WFC (e.g., Greene & Burleson, 2008; Mathieu et al., 2019).

Importantly, these insights also suggest that organisational policies should be designed in ways that recognise and respond to cultural norms surrounding family responsibility. For example, most parent participants described receiving caregiving support from elderly parents, which may reduce the perceived need for flexible working arrangements to manage childcare when compared with Western contexts. However, further research is needed to examine how culturally specific family structures and expectations shape employees’ preferences for, and responses to, different forms of workplace support.

## 7. Conclusions

In conclusion, this study openly explored and provided insight into the WFC phenomenon in contemporary Chinese society. The attitude towards WFC (i.e., just a lived experience) and the strong sense of family responsibility (i.e., family come first) seem to affect the experience of WFC and its related coping strategies. In addition, the experience of WFC also seems to be associated with ideas of filial piety, parental expectation, financial insecurity, whether one is the only child of the family, the health of family members, a friendly work environment (i.e., schedule flexibility and supervisor and co-worker support), family support (i.e., spouse, sibling, and elder parents support), income level, gender, work/family-related distress, emotion (i.e., guilt, anger), performance, and well-being.

## Figures and Tables

**Table 1:** Background Information of the Participants in the Present Study.

	Gender	Age	Occupation	Income (per Month)	Marital Status	Child	Living with
P1	Male	29	Pet shop owner; part-time Didi driver (similar to Uber driver)	Average 7000 RMB	Married	A 2-year-old daughter	wife and daughter
P2	Male	28	Owner of a business plan services company	4000 RMB	In a relationship	No child	Girlfriend and elderly parents
P3	Female	24	Work at an exhibition company	7–8000 RMB	Single	No child	Elderly mother
P4	Female	28	Work at a bank	8000–10,000 RMB	In a relationship	No child	Elderly parents
P5	Male	34	Insurance broker	No comment	Married	An 8-month-old boy	Wife, child, and elderly parents
P6	Male	35	Insurance broker	At least 8000 RMB	Married	No child	Wife and elderly parents
P7	Female	39	University English lecturer; owner of an English learning studio	10,000 RMB (university salary)	Married	A 12-year-old son	Husband and child
P8	Female	35	Financial planner	At least 20,000 RMB	Divorce	Two sons, one is 3 years old, and the other is 1 year old	The younger son and elderly parents
P9	Female	26	Primary school teacher	10,000–20,000 RMB	Single	No child	Elderly parents
P10	Female	38	Restaurant owner	No comment	Married	A 5-year-old daughter	Daughter, older sister, and elderly mother
P11	Male	26	IT company owner	50,000–100,000 RMB	Single	No child	Elderly parents
P12	Female	42	Nurse	33,000 RMB	Married	Two daughters, both are 6 years old	Husband, daughters, and nanny
P13	Male	26	News reporter	10,000–20,000 RMB	Married	No child	Wife
P14	Male	46	Government officer	Around 22,000 RMB	Married	A 14-year-old daughter and a 3-year-old son	Wife and children
P15	Male	53	Sales	7000 RMB	Married	A 22-year-old daughter and a 13-year-old son	Wife and children
P16	Male	41	Sales	No comment	Married	A 6-month-old daughter	Wife, child, and elderly parents

**Table 2:** Interview Schedule.

Background Information
Gender Age Marital status (single, married, years of marriage, divorced) Child (number of children, age of children) Living situation (live by him/herself or live with parents, spouse, or children) Do your other family member(s) work? If so, full-time or part-time? Brief job description
WIF
Do you encounter any difficulties at work? If yes, what difficulties do you have in your work? Can you describe the WIF you are experiencing? Does the WIF you mentioned earlier affect you the most? Or do you think another WIF is much worse? Can you give me an example? How often do you experience WIF? Can you describe how it affects you both mentally and physically? How do you cope with this type of WIF? Does or did anyone or anything help you improve these problems?
In the above questions, if the participants are unclear, I will offer more details, such as: “Please think of a time in your life when you are experiencing WIF; when you have a situation in mind, please describe it to me.” “Did the difficulty at work influence your family role, such as not having enough time for family members or fulfilling home responsibilities?”
FIW
Do you encounter any difficulties at work? If yes, what difficulties do you have in your work? Can you describe the FIW you are experiencing? Does the FIW you mentioned earlier affect you the most? Or do you think other FIW are much worse? Can you give me an example? How often do you experience the FIW? Can you describe how it affects you both mentally and physically? How do you cope with this type of FIW? Does or did anyone or anything help you improve these problems?
In the above questions, if the participants were unclear, I would offer more details, such as: “Please think of a time in your life when you are experiencing FIW; when you have a situation in mind, please describe it to me.” “Did the difficulty at work influence your family role, such as not having enough time for family members or fulfilling home responsibilities?”

Note. WIF = work-to-family conflict, FIW = family-to-work conflict.

**Table 3:** Examples of Significant Statements and their Formulated Meanings.

Significant Statements	Formulated Meanings
If I am not in this kind of occupation, if I am not a salesman, and my job requires me to work from 9 AM to 5 PM, nine-nine-six (9 AM to 9 PM, 6 working days per week), or my work time is fixed. I will have no control over dealing with things at home.	Flexible working hours at work help to decrease the family-to-work conflict.
I keep telling myself, don’t bring back the emotion at work to my family. I mean, like the bad emotion at work. But I am just a human, so I couldn’t just let it stop.	It is hard not to let the emotion at work affect the emotion at home.
If you are an only child, you probably need to take care of 8 elderly parents. Your parents, your grandparents, your spouse’s parents, and your spouse’s grandparents.	An only child has more eldercare responsibility.
It is impossible! I mean, she (my daughter) has to go to school in the morning at 8 AM, but I am already working at that time, then school is over at 4 PM, and I am still at work, so what can I do?	An incompatibility between work demands and family needs.

**Table 4:** Themes and Subthemes of this Study.

Theme	Sub-Theme
Normalised Conflict - A perception or attitude where the participant describes work–family conflict not as a major problem, but as a common, unavoidable, or “normalised” aspect of daily life; a “life experience” that must be endured (e.g., Life goes on. So, you cry about it, then just keep moving forward (P8).)	Turn a blind eye
Cultural Role Norms - Encompasses the cultural values, beliefs, and traditional expectations specific to Chinese society that shape an individual’s understanding of their work and family roles and responsibilities (e.g., The domestic chore should be done by the wife, that’s just part of our culture (P14).)	Work is for family United relationship Traditional gender role Filial piety
Family-Driven Interference - Specific family-domain demands, responsibilities, or events that interfere with or spill over into the work domain, consistent with FIW (e.g., If you are an only child, you probably need to take care of 8 elderly parents. Your parents, your grandparents, your spouse’s parents, and your spouse’s grandparents (P11).)	Health of family members Having children Parental expectation Being the only child
Work-Driven Interference - Specific work-domain demands, characteristics, or events that interfere with or spill over into the family domain, consistent with WIF (e.g., Money can solve problems, so you just have to endure the pressure from work (P6).)	Money is the cure Occupational difference
Affective Spillover - The transfer of emotions, moods, and stress from one domain (work or family) to the other, influencing psychological well-being (e.g., When I lose my temper at home, I feel guilty afterwards (P9).)	Work distress Display of anger Guilty feelings
Cyclical Strain - The perceived negative outcomes or consequences of WFC, which often create a self-perpetuating “vicious cycle” of strain that degrades performance and wellbeing in both domains (e.g., It becomes a vicious cycle. You have less energy because of the conflict, then you don’t handle either side well, and the conflict just keeps going (P13).)	A vicious cycle Denied opportunities Become lazy

Note. WFC = work–family conflict, FIW = family-to-work conflict, WIF = work-to-family conflict.

## Data Availability

The data presented in this study are available on request from the corresponding author due to privacy reasons.

## Supplementary Materials

The following supporting information can be downloaded at: https://www.mdpi.com/article/10.3390/bs16010063/s1, Figure S1: Theme tree; Table S1: Codebook; Table S2: Thematic chart; Table S3: Audit trail snapshot.

## References

[B1-behavsci-16-00063] Agars M. D., French K. A. (2011). What if work and family research actually considered workers and their families?. Industrial and Organisational Psychology.

[B2-behavsci-16-00063] Aldao A., Nolen-Hoeksema S., Schweizer S. (2010). Emotion-regulation strategies across psychopathology: A meta-analytic review. Clinical Psychology Review.

[B3-behavsci-16-00063] Allen T. D., French K. A., Dumani S., Shockley K. M. (2020). A cross-national meta-analytic examination of predictors and outcomes associated with work–family conflict. Journal of Applied Psychology.

[B4-behavsci-16-00063] Aycan Z., Korabik K., Lero D. S., Whitehead D. L. (2008). Cross-cultural approaches to work-family conflict. Handbook of work-family integration.

[B5-behavsci-16-00063] Bakker A. B., Demerouti E., Dollard M. F. (2008). How job demands affect partners’ experience of exhaustion: Integrating work-family conflict and crossover theory. Journal of Applied Psychology.

[B6-behavsci-16-00063] Balenzano C., Gemmano C. G., Manuti A. (2025). Child-rearing involvement and parental emotional exhaustion: The moderating role of supportive relationships for mothers and fathers in Italian dual-earner families. Child & Family Social Work.

[B7-behavsci-16-00063] Borgmann L. S., Rattay P., Lampert T. (2019). Health-related consequences of work-family conflict from a European perspective: Results of a scoping review. Frontiers in Public Health.

[B8-behavsci-16-00063] British Psychological Society (BPS) (2020). Ethics best practice guidance on conducting research with human participants during COVID-19.

[B9-behavsci-16-00063] Byron K. (2005). A meta-analytic review of work-family conflict and its antecedents. Journal of Vocational Behavior.

[B10-behavsci-16-00063] Casares R. J., White C. C. (2018). The phenomenological experience of parents who live with a boomerang child. The American Journal of Family Therapy.

[B11-behavsci-16-00063] Cerrato J., Cifre E. (2018). Gender inequality in household chores and work-family conflict. Frontiers in Psychology.

[B12-behavsci-16-00063] Chen S., Cheng M.-I. (2024). The experience of work–family conflict: Does being the only child matter?. Journal of Family Issues.

[B13-behavsci-16-00063] Chen S., Cheng M. I., Elqayam S., Mark S. (2023). Developing and testing an integrative model of work-family conflict in a Chinese context. Current Psychology.

[B14-behavsci-16-00063] Chen X. (2024). The gendered division of housework in China: Parenthood effects and heterogeneity across parenthood stages. Population Research and Policy Review.

[B15-behavsci-16-00063] Cheng C., Lo B. C. Y., Chio J. H. M., Bond M. H. (2010). The Tao (way) of Chinese coping. The Oxford handbook of Chinese psychology.

[B16-behavsci-16-00063] Choo J. L. M., Desa N. M., Asaari M. H. A. H. (2016). Flexible working arrangement toward organisational commitment and work-family conflict. Studies in Asian Social Science.

[B17-behavsci-16-00063] Ciabattari T. (2007). Single mothers, social capital, and work-family conflict. Journal of Family Issues.

[B18-behavsci-16-00063] Civil Aviation Administration of China (2020). Notice on further reducing international passenger flights during the epidemic prevention and control period.

[B19-behavsci-16-00063] Creswell J. W. (2013). Qualitative inquiry & research design: Choosing among five approaches.

[B20-behavsci-16-00063] Direnzo M. S., Greenhaus J. H., Weer C. H. (2011). Job level, demands, and resources as antecedents of work-family conflict. Journal of Vocational Behavior.

[B21-behavsci-16-00063] El-Kassem R. C. (2019). Antecedents and consequences of work-family conflict in Qatar. The Journal of Social Sciences Research.

[B22-behavsci-16-00063] Favez N., Max A., Bader M., Tissot H. (2023). When not teaming up puts parents at risk: Coparenting and parental burnout in dual-parent heterosexual families in Switzerland. Family Process.

[B23-behavsci-16-00063] Galovan A. M., Fackrell T., Buswell L., Jones B. L., Hill E. J., Carroll S. J. (2010). The work–family interface in the United States and Singapore: Conflict across cultures. Journal of Family Psychology.

[B24-behavsci-16-00063] Gemmano C. G., Manuti A., Girardi S., Balenzano C. (2023). From conflict to balance: Challenges for dual-earner families managing technostress and work exhaustion in the post-pandemic scenario. International Journal of Environmental Research and Public Health.

[B25-behavsci-16-00063] Giancaspro M. L., Gemmano C. G., Manuti A. (2023). The art of designing work: Work/Family interface as a mediator in the relationship between work design, burnout, and performance. Behavioral Sciences.

[B26-behavsci-16-00063] Giorgi A. (2006). Concerning variations in the application of the phenomenological method. The Humanistic Psychologist.

[B27-behavsci-16-00063] Greene J. O., Burleson B. R. (2008). Handbook of communication and social interaction skills.

[B28-behavsci-16-00063] Gui T., Koropeckyj-Cox T. (2016). I am the only child of my parents:“ Perspectives on future elder care for parents among Chinese only-children living overseas. Journal of Cross Culture Gerontology.

[B29-behavsci-16-00063] Hennink M. M., Liamputtong P. (2008). Language and communication in cross-cultural qualitative research. Doing cross-cultural research: Ethical and methodological perspectives.

[B30-behavsci-16-00063] Hennink M. M., Kaiser B. N., Marconi V. C. (2017). Code saturation versus meaning saturation: How many interviews are enough?. Qualitative Health Research.

[B31-behavsci-16-00063] Huffman A., Culbertson S. S., Henning J. B., Goh A. (2013). Work-family conflict across the lifespan. Journal of Managerial Psychology.

[B32-behavsci-16-00063] Innstrand S. T., Langballe E. M., Falkum E. (2010). Exploring occupational differences in work-family interaction: Who is at risk?. International Journal of Stress Management.

[B33-behavsci-16-00063] Ji L.-J., Yap S., Khei Z. A. M., Wang X., Chang B., Shang S. X., Cai H. (2022). Meaning in stressful experiences and coping across cultures. Journal of Cross-Cultural Psychology.

[B34-behavsci-16-00063] Kaufert J. M., Putsch R. W. (1997). Communication through interpreters in healthcare: Ethical dilemmas arising from differences in class, culture, language, and power. The Journal of Clinical Ethics.

[B35-behavsci-16-00063] Korabik K., Mills M. (2015). The intersection of gender and work-family guilt. Gender and the work-family experience.

[B36-behavsci-16-00063] Korabik K., Lero D. S., Whitehead D. L. (2008). Handbook of work-family integration: Research, theory, and best practices.

[B37-behavsci-16-00063] Kossek E. E., Baltes B. B., Matthews R. A. (2011). Innovative ideas on how work-family research can have more impact. Industrial and Organisational Psychology.

[B38-behavsci-16-00063] Lam W. W. Y., Jor T., Yu W., Fielding R. (2022). The demands and resources of working informal caregivers: A systematic review. Work & Stress.

[B39-behavsci-16-00063] Lawrence E. R., Hallbesleben J. R. B., Paustian-Underdahl S. C. (2013). The influence of workplace injuries on work-family conflict: Job and financial insecurity as mechanisms. Journal of Occupational Health Psychology.

[B40-behavsci-16-00063] Li P., Chen X. (2024). Gender dynamics in dual-earner couples: Spousal occupational status and working hours. Economic and Industrial Democracy.

[B41-behavsci-16-00063] Liamputtong P. (2010). Cross-cultural communication and language issues. Performing qualitative cross-cultural research.

[B42-behavsci-16-00063] Lin X., Lin Y., Hu Z., Alias H., Wong L. (2021). Practice of new normal lifestyles, economic and social disruption, and level of happiness among general public in China in the post-COVID-19 era. Risk Management and Healthcare Policy.

[B43-behavsci-16-00063] Lincoln Y. S., Guba E. G. (1985). Naturalistic inquiry.

[B44-behavsci-16-00063] Lu L., Cooper C. L. (2015). Handbook of research on work-life balance in Asia.

[B45-behavsci-16-00063] Lu L., Gilmour R., Kao S.-F., Huang M.-T. (2006). A cross-cultural study of work/family demands, work/family conflict and well-being: The Taiwanese vs. British. Career Development International.

[B46-behavsci-16-00063] Mansilla-Domínguez J. M., Recio-Vivas A. M., Lorenzo-Allegue L., Cachón-Pérez J. M., Esteban-Gonzalo L., Palacios-Ceña D. (2024). The role of duty, gender and intergenerational care in grandmothers’ parenting of grandchildren: A phenomenological qualitative study. BMC Nursing.

[B47-behavsci-16-00063] Mathieu M., Eschleman K. J., Cheng D. Q. (2019). Meta-analytic and multiwave comparison of emotional support and instrumental support in the workplace. Journal of Occupational Health Psychology.

[B48-behavsci-16-00063] McGinnity F., Russell H., Gallie D. (2013). Work-family conflict and economic change. Economic crisis, quality of work and social integration: The European experience.

[B49-behavsci-16-00063] McMullan A. D., Lapierre L. M., Li Y. (2018). A qualitative investigation of work-family-supportive coworker behaviors. Journal of Vocational Behavior.

[B50-behavsci-16-00063] Namdari S., Nasiri A., Nakhaee S., Taheri F. (2019). Exploring the effects of nurses’ family-work conflict on patient care quality: A qualitative study. Modern Care Journal.

[B51-behavsci-16-00063] Neubert L., König H.-H., Brettschneider C. (2021). Dementia caregiving and employment: A mixed-studies review on a presumed conflict. Ageing & Society.

[B52-behavsci-16-00063] Odle-Dusseau H. N., Matthews R. A., Wayne J. H. (2018). Employees’ financial insecurity and health: The underlying role of stress and work-family conflict appraisals. Journal of Occupational and Organizational Psychology.

[B53-behavsci-16-00063] Opdenakker R. (2006). Advantages and disadvantages of four interview techniques in qualitative research. Forum Qualitative Social Research Sozialforschung.

[B54-behavsci-16-00063] Penley J. A., Tomaka J., Wiebe J. S. (2002). The association of coping to physical and psychological health outcomes: A meta-analytic review. Journal of Behavioral Medicine.

[B55-behavsci-16-00063] Rehman S., Roomi M. (2012). Gender and work-life balance: A phenomenological study of women entrepreneurs in Pakistan. Journal of Small Business and Enterprise Development.

[B56-behavsci-16-00063] Reimann M., Schulz F., Huyer-May B. (2022). The family side of work–family conflict: A literature review of antecedents and consequences. Journal of Family Research.

[B57-behavsci-16-00063] Roskam I., Mikolajczak M. (2020). Gender differences in the nature, antecedents and consequences of parental burnout. Sex Roles.

[B58-behavsci-16-00063] Roth L., David E. M. (2009). Work-family conflicts and work performance. Psychological Reports.

[B59-behavsci-16-00063] Ryen A., Gubrium J. F., Holstein J. A. (2002). Cross-cultural interviewing. Handbook of interview research.

[B60-behavsci-16-00063] Shaffer M. A., Joplin J. R. W., Hsu Y.-S. (2011). Expanding the boundaries of work–family research: A review and agenda for future research. International Journal of Cross-Cultural Management.

[B61-behavsci-16-00063] Shavarini M. K. (2006). The role of higher education in the life of a young Iranian woman. Women’s Studies International Forum.

[B62-behavsci-16-00063] Shockley K. M., Douek J., Smith C. R., Yu P. P., Dumani S., French A. K. (2017). Cross-cultural work and family research: A review of the literature (2002–2012). Journal of Vocational Behavior.

[B63-behavsci-16-00063] Sirgy M. J., Lee D. J., Park S., Joshanloo M., Kim M. (2020). Work–family spillover and subjective well-being: The moderating role of coping strategies. Journal of Happiness Studies.

[B64-behavsci-16-00063] Smith J. A., Flowers P., Larkin M. (2021). Interpretative phenomenological analysis: Theory, method and research.

[B65-behavsci-16-00063] Stertz A. M., Wiese B. S. (2024). Dual-earner couples’ gender role attitudes and their parental leave decisions: A longitudinal study of partner influences. Sex Roles.

[B66-behavsci-16-00063] Sun A., Xia F., Zhang X. (2025). The motherhood penalty on health: Evidence from China. Journal of Economic Behavior & Organization.

[B67-behavsci-16-00063] Vitoria B. D. A., Ribeiro M. T., Carvalho V. S. (2022). The work-family interface and the COVID-19 pandemic: A systematic review. Frontiers in Psychology.

[B68-behavsci-16-00063] Wallace J. (2005). Job stress, depression and work-to-family conflict: A test of the strain and buffer hypotheses. Relations Industrielles/Industrial Relations.

[B69-behavsci-16-00063] Wang H. M., Ma A. L., Guo T. T. (2020). Gender concept, work pressure, and work-family conflict. American Journal of Men’s Health.

[B70-behavsci-16-00063] Xu Y., Farver J. A. M., Zhang Z., Zeng Q., Yu L., Cai B. (2005). Mainland Chinese parenting styles and parent-child interaction. International Journal of Behavioral Development.

[B71-behavsci-16-00063] Yucel D., Chung H. (2023). Working from home, work–family conflict, and the role of gender and gender role attitudes. Community, Work & Family.

[B72-behavsci-16-00063] Zhang J., Jin S., Li T., Wang H. (2021). Gender discrimination in China: Experimental evidence from the job market for college graduates. Journal of Comparative Economics.

[B73-behavsci-16-00063] Zhang M., Zhao K., Korabik K. (2019). Does work-to-family guilt mediate the relationship between work-to-family conflict and job satisfaction? Testing the moderating roles of segmentation preference and family collectivism orientation. Journal of Vocational Behavior.

[B74-behavsci-16-00063] Zhang X., Chen W. (2023). Does grandchild care intention, intergenerational support have an impact on the health of older adults in China? A quantitative study of CFPS data. Frontiers in Public Health.

