# Supplementary material for: “It Is Not Possible to Balance It Easily”: A Phenomenological Study Exploring the Experience of Work–Family Conflict in Contemporary Chinese Society
Source: Behav Sci (Basel). 2025 Dec 30;16(1):63. doi: 10.3390/bs16010063 (PMC12837297; doi:10.3390/bs16010063)
Supplement: Supplementary file 1 [file behavsci-16-00063-s001.zip › Figure S1 Theme tree.pdf]

Theme 1: That is life

1. Neglect
2. Self-regulation

Theme 2: Sense of family

1. Work is for family
2. Gender role
3. Filial piety
4. Child expectation

Theme 3: Work

1. Personal income
2. Work distress
3. Working hours
    - Long working hours
    - Flexible working hours
4. The nature of work
    - Supportive environment

Theme 4: Family

1. Health of the families
2. Children
    - Age of the child
    - Education of child
3. Single child
4. Financial status

Theme 5: work can wait

1. Career opportunities
2. Incomes
3. Work performance

Theme 6: Take off the mask

1. Abreaction
    - Anger
2. Family atmosphere
3. Housework

Theme 7: A vicious cycly

1. Anger
2. Depress
3. Guilty

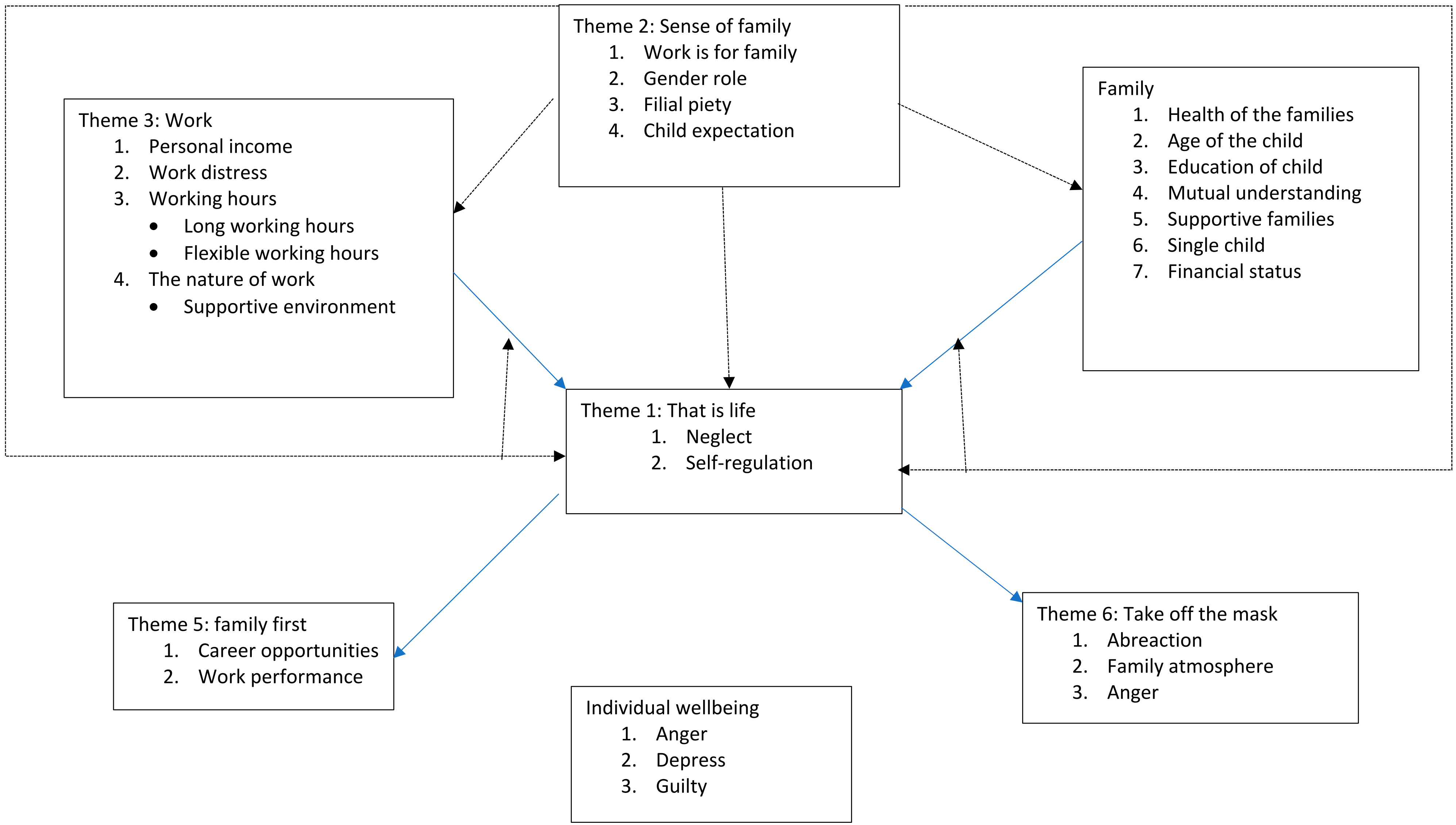

Theme 2: Sense of family
1. Work is for family
2. Gender role
3. Filial piety
4. Child expectation
Theme 3: Work
1. Personal income
2. Work distress
3. Working hours
• Long working hours
• Flexible working hours
4. The nature of work
• Supportive environment
Family
1. Health of the families
2. Age of the child
3. Education of child
4. Mutual understanding
5. Supportive families
6. Single child
7. Financial status
Theme 1: That is life
1. Neglect
2. Self-regulation
Theme 5: family first
1. Career opportunities
2. Work performance
Individual wellbeing
1. Anger
2. Depress
3. Guilty
Theme 6: Take off the mask
1. Abreaction
2. Family atmosphere
3. Anger
