# Supplementary material for: “It Is Not Possible to Balance It Easily”: A Phenomenological Study Exploring the Experience of Work–Family Conflict in Contemporary Chinese Society
Source: Behav Sci (Basel). 2025 Dec 30;16(1):63. doi: 10.3390/bs16010063 (PMC12837297; doi:10.3390/bs16010063)
Supplement: Supplementary file 1 [file behavsci-16-00063-s001.zip › Table S1 Codebook.pdf]

Table S1. Codebook	
**Theme (Level 1 Code) & Sub-theme (Level 2 Code)**	**Definition**
**1.0 Normalised conflict**	A perception or attitude where the participant describes work-family conflict not as a major problem, but as a common, unavoidable, or "normalised" aspect of daily life; a "life experience" that must be endured.
1.1 Turn a blind eye	An avoidant coping strategy in response to normalized conflict, where the individual consciously chooses to ignore the conflict ("Just don't think about it"), act as if it didn't happen, or wait for it to pass without direct action.
**2.0 Cultural role norms**	Encompasses the cultural values, beliefs, and traditional expectations specific to Chinese society that shape an individual's understanding of their work and family roles and responsibilities.
2.1 Work is for family	A deeply held belief that the primary purpose of work is instrumental. Work is described as a way to "provide a better life for their family" and fulfil obligations, rather than a source of personal achievement.
2.2 United relationship	Describes the supportive, collective, and intergenerational nature of the family, where members (especially elderly parents) provide tangible support (e.g., childcare, cooking, financial help) that can ease the experience of WFC.
2.3 Traditional gender role	The culturally ingrained belief that men and women have distinct responsibilities, described as "part of our culture." This typically involves women assuming the primary role of caregiver and housekeeper ("women should stay at home") while men are the main providers.
2.4 Filial piety	The cultural and moral obligation to care for, support, and show respect to one's elderly parents ("you, as the child, must take care of them"). This code captures both the act of caregiving and the psychological pressure or sense of future responsibility associated with this duty.

**3.0 Family-driven interference**	Specific family-domain demands, responsibilities, or events that interfere with or spill over into the work domain, consistent with Family-to-Work (FIW) conflict.
3.1 Health of family members	An acute or chronic health issue concerning a family member (child or parent) that requires the participant to immediately stop work ("I will definitely stop everything at work") or take time off to provide care.
3.2 Having children	The demands and responsibilities associated with childcare, which participants report lead to more stress as children age (This code is linked to 3.3).
3.3 Parental expectation	The pressure parents feel to ensure their children's educational success. This code captures the time ("take him to... after-school classes"), financial cost, and anxiety ("invisible pressure") related to homework supervision and aspirations for their children's future.
3.4 Being the only child	The participant's status as an only child. This status is perceived to "exacerbate the negative impact of filial piety" and creates panic or worry about their sole ability to provide eldercare in the future due to a "lack of sibling support".
**4.0 Work-driven Interference**	Specific work-domain demands, characteristics, or events that interfere with or spill over into the family domain, consistent with Work-to-Family (WIF) conflict.
4.1 Money is the cure	A perception that financial stress is the root cause of WFC ("the biggest problem in my family right now is the money") and that higher or more stable income would resolve most, if not all, of the conflict.
4.2 Occupational difference	How the specific characteristics of one's job impact the level of WIF. This includes factors such as "long working hours" (e.g., 996), inflexible schedules (e.g., shift work), "flexible working hours," and the presence (or absence) of a "supportive work environment".
**5.0 Affective spillover**	The transfer of emotions, moods, and stress from one domain (work or family) to the other, influencing psychological well-being.

5.1 Work distress	The negative psychological state ("emotion at work") or "physical exhaustion" ("I am so tired at work") originating from work demands that spills over into the home environment.
5.2 Display of anger	An externalizing coping mechanism where stress or exhaustion from WFC manifests as anger, yelling ("I yelled [at my children]"), or venting directed at family members ("I get angry for no reason [at home]").
5.3 Guilty feelings	An internal emotional response of guilt or shame. This is caused by either (a) failing to meet family obligations (e.g., "I feel guilty. I didn’t fulfil the obligation") due to work, or (b) inappropriate behaviour (like anger) towards family members ("I thought about it afterwards… it’s my fault").
**6.0 Cyclical strain**	The perceived negative outcomes or consequences of WFC, which often create a self-perpetuating "vicious cycle" of strain that degrades performance and well-being in both domains.
6.1 A vicious cycle	The perception that WFC is a reinforcing loop where stress from one domain leads to poor outcomes (e.g., bad mood, lack of sleep, poor health), which in turn diminishes resources ("you don’t have enough energy") and performance in the other domain.
6.2 Denied opportunities	A tangible, negative career outcome where a participant must refuse or lose a work-related opportunity (e.g., "lost a potential business opportunity," "lost the development opportunity") due to family responsibilities.
6.3 Become lazy	A behavioural outcome, described by participants as "laziness," where exhaustion from work results in an unwillingness or inability to perform tasks ("Don’t want to do housework") in the family domain, or vice-versa.
