# Supplementary material for: “It Is Not Possible to Balance It Easily”: A Phenomenological Study Exploring the Experience of Work–Family Conflict in Contemporary Chinese Society
Source: Behav Sci (Basel). 2025 Dec 30;16(1):63. doi: 10.3390/bs16010063 (PMC12837297; doi:10.3390/bs16010063)
Supplement: Supplementary file 1 [file behavsci-16-00063-s001.zip › Table S2 Thematic chart.pdf]

Table S2. Thematic chart

Interviewee 1
Male, 29 years old, self-employed, running a pet shop, small business owner, part-time uber driver, income – average 7000RMB per month; married 3 years, one daughter, 2 years old, living with wife and daughter, wife has no job at the moment, full-time housewife, his parents will take care his daughter and give him living expenses occasionally. (20 significant statements)

1. Income	Increase job distress, affect relationship with wife. Income are mitigating factor of WFC 我屋企铺头生意唔好，可能收入唔多，工作压力大，跟住太太又带小朋友有压力，2 个就有小小情绪，甘样咯，然后就有小小拗撬. 你话会唔会严重，哩个要取决于经济上咯，因为讲老实，我地屋企依家最大嘅问题系经济来源，所以如果稳到钱嘅，可以稳到钱嘅，可以先保障小朋友再保障屋企嘅，甘当然就会好好多啦	My business isn't doing well, so our income might not be much. Work pressure is high, and then my wife also feels stressed taking care of the kids. So the two of us have a bit of emotional strain, and then we end up having some small arguments.<br><br>As for whether it will become serious, that depends on the financial situation. To be honest, the biggest problem in our family right now is our source of income. So if we can earn money—if we can find a way to make money—so we can first make sure the kids are taken care of and then the household is taken care of, then of course things will be a lot better.
2. workload	比如我返到屋企，可能我又好累，又唔想做嘢，压	For example, when I get home, I might be really tired, and I

	力又好大…甘大家都累，有压力，跟住然后返到屋企，比如话佢又唔想做家务，唔想洗碗之类啦，跟住又，我又唔想搞，呃，嗜系，大家都有嘚面色咯，跟住可能讲一两句就可能话，佢就话你点点点啊，跟住我就唔爽又摔嘴啊之类甘样，跟住就开始嗌交了 Doesn’t want to do housework after work, leads to fight with wife.	don’t feel like doing anything. The pressure is also huge… so everyone is tired and stressed. Then when we get home, say she also doesn’t feel like doing housework—like washing the dishes and so on—and then I also don’t feel like dealing with it. So we both show it on our faces. Then maybe after saying one or two sentences, it turns into, she says “Why are you like this and that…”, and then I feel unhappy and talk back, and then it starts turning into an argument—then we start yelling at each other.
3. Neglect<br>3.1. The sole source of income for a family	WFC is not a problem at all, it is a daily thing that everyone will experience in their life. The interviewee’s job as the sole source of income for his family might increase the neglect of WFC/FWC 因为讲老实果句，工作系为左家庭，唔为大，都要为个细（小孩），甘哩嘚嘢冇话拿自己身体来教飞，甘紧系要充足睡眠，食饭，先有力稳钱，所以话其实发生左（冲突），都唔会去捻咯，都系做返自己嘢.	To be honest, work is for the family—if not for the whole family, then at least for the child. So for these things, there’s no way you should sacrifice your own health. You definitely need enough sleep and proper meals so you have the energy to earn money. So even if a conflict happens, I won’t really dwell on it—I just go back to doing what I need to do.

4. Marriage	Man should be the provider of the family, should take on more responsibilities, don't need to blow off steam, it is my duty to provide for the family, have to deal with the problem himself. (WFC)系啊，都系噶了，因为哩嘚冇咩好发泄啊，又唔系话以前(before marriage)，就话发泄下，依家唔会了...需要去发泄哩样野咯，但依家反而真系冇哩种需要了. 啱有嘚嘢要自己识的去承受啊你明唔明啊，哩个可能就系结左婚同未结婚噶情况吧，啱系有嘚情况有嘚时候就一定要自己去承受返果种问题，要自己捻办法去解决	Yeah, that's how it is. There isn't really anything to "vent" about. It's not like before—before marriage—when you could just blow off steam a bit. Now it's not like that anymore… it's not that I need to vent in that way. In fact, I really don't have that kind of need anymore.<br><br>Some things you have to know how to carry yourself, you know what I mean? That might be the difference between being married and not being married. I mean, in some situations, you just have to bear those problems yourself—you have to think of a way to solve them on your own.
5. Family of origin<br>5.1. Financial status	Not from a rich family. Have to work harder 因为本身家境又唔系好富裕，所以都要，都要稳（钱）咯<br>除非你地屋企，系本身就比较富裕，唔需要担心物质方面噶嘢, 甘你就可能暂时唔会出现甘噶问题	Because my family's financial situation isn't well-off to begin with, we still have to earn money.<br><br>Unless your family is already relatively well-off and you don't need to worry about material/financial things—then you

		might not run into this kind of problem, at least for the time being.
6. Interrelationship<br>6.1. Parents and spouse<br>6.2. Relationship with spouse	Affect work performance. The bad relationship between his parents and spouse, makes him feel that he has one more role - mediator 太太同奶奶可能有嘚拗撬咯…哩嘚婆媳关系，唔需要我多讲啊嘛（苦笑）…性格不合啦，一句概括晒，唔想太多讲哩个，好嚿啊…前期可能会(affect work performance)，但系后来慢慢都，自己都适应左都习惯左了已经.<br>最初期就系想离婚啦，但后来捻下捻下又，睇化左就唔理佢（感受）咯，唔去捻咯，冇咩捻法之后就，啲系正常就过 1-2 日就冇事噶啦<br>继而屋企人又同太太相处的好好，又帮手带小朋友，甘你就可能暂时唔会出现甘噶问题	My wife and my mother might have some quarrels… this kind of mother-in-law/daughter-in-law relationship doesn't need me to say much (wry smile)… it's just that their personalities don't match. That sums it up. I don't really want to talk too much about it—it's pretty exhausting…<br><br>At the beginning, it might affect work performance, but later on, little by little, I adapted and got used to it.<br><br>At the very start, I even wanted a divorce, but after thinking it over and over, I came to terms with it and just didn't bother about it (emotionally). I don't think about it—there's not much point. After that, normally in one or two days, it's fine again.

		And then if your family members actually get along really well with your wife and also help take care of the kids, then you might not run into this kind of problem for the time being.
7. Children<br>7.1. Age of child<br>7.2. Education	WFC grow with children's age. Education will affect WFC (FIW or WIF).<br>因为你要知道侬家小朋友系国内上幼儿园，一个月又要多洗千几蚊，又多一份压力噶了喔，所以又要付出多小小了，所以都可能会演变成一个更加之一个恶劣噶表现啦，或者系一个更加之恶劣噶激发点啦，哩个唔出奇噶<br>小朋友有嘢咩问题要你地帮手噶，甘既然噶话，其实都会出现有一个新噶矛盾点，啫系比如就系小朋友系学习学的唔好，老婆有老婆返工忙，你有你噶返工忙，甘突然间老师一个电话过来话你睇下你教到个仔点，你睇下你教到个女点？跟住话啊个仔你冇份来照顾噶（于太太双方间指责）？其实大家都忙，大家都冇时间	Because you have to understand that right now, if the child is in kindergarten in China, it can cost an extra thousand-plus yuan a month, which adds another layer of pressure. So you have to put in a bit more, and it could end up turning into an even worse situation, or a bigger trigger point—so that wouldn't be surprising.<br><br>If the child has issues that require you both to step in and help, then naturally a new point of conflict can appear. For example, if the child isn't doing well in learning, your wife is busy with her job, and you're busy with yours. Then suddenly the teacher calls and says, "Look at how you're teaching your son—look at how you're

		teaching your daughter!" And then it turns into, "You don't even take responsibility for looking after the kid!" (meaning blaming each other as a couple). But in reality, both of you are busy, and neither of you has time.
8. Health of the families<br>8.1. Children's health	Has to stop the job.<br>小朋友突然唔舒服啊，甘咪返去（家）咯	If the child suddenly isn't feeling well, then you'd go back home.
9. Year of marriage	At the beginning of marriage, WIF/FIW will have stronger negative effect, doesn't not related to the relationship with spouse, just because he gets used to the conflict.<br>（家庭影响工作心情）又唔，都系果句啦，以前会，岩结婚果一年会，但依家唔会了，依家捻开捻化左了	Whether family affects my mood at work… it's the same thing I said before: it used to, during the first year right after getting married, but now it doesn't anymore. Now I've thought it through and come to terms with it.
10. (Mutual) understanding<br>10.1. With partner	Wife knows he has to work very hard, more willing to do housework, support him.<br>太太肯定会理解要稳钱噶，甘讲老实，你仲捻住固定时间朝九晚五咩，有钱蚊就紧系宁可跑夜嘚做嘢嘚都要做噶啦…有时我返工夜，点几 2 点（凌晨），佢都整晒其他嘢了，甘佢都冇话留比我整，啱话其实大家都理解噶	My wife will definitely understand that we need to earn money. To be honest, are you still thinking about fixed working hours like nine to five? If there's money to be made, of course you'd rather work later into the night—you'd still do it. Sometimes I get home from work really late, like 1 or 2

		a.m., and she's already taken care of everything else. She doesn't leave it for me to do. So actually, we both understand each other.

Interviewee 2

Male, 28 years old, self-employed, one of the bosses in a business plan services company, fixed salary – around 4,000RMB per month, has annual bonus; living with parents and girlfriend, no child, all family members have full-time job, but mom is re-employ after retirement, doesn't have to pay rent and living expense to his parents. (8 significant statements)

1. The perceived of need<br>1.1. Change of the society	Has to work harder, to keep learning to catch up the change of society (time)<br>啱系专业唔对口，同埋哩个年代佢噶节奏啊，尤其是，我了解噶就系中国来计啦，变化的太快，跟住你要去不停甘吸收好多嘢，先至跟的到，跟的到个变化，所以你唔会比时代抛弃咯	It's mainly that your field doesn't match your major, and also the pace in this era—especially, from what I understand about China—the changes are happening too fast. You have to keep absorbing a lot of new things continuously in order to keep up with the changes, so you won't be left behind by the times.
2. Relationship with employees	Generation differences<br>公司老板比噶工作，就佢地果个理解就会有小小似老师比噶作业甘，甘你（员工）做唔好，或者 hea 做，甘或者佢自己点样都好，佢冇真正觉得系一份工作，系你做唔好系会阜人炒噶，而且，有嘚员工佢亦都唔介意，啱系好后生果嘚，甘炒咪炒咯，系甘噶了，我（员工）就系	The work the boss assigns—based on how they understand it—is a bit like homework a teacher gives. So if you (as an employee) don't do it well, or you do it half-heartedly, then no matter what, they don't truly feel it's a "job" in the sense that if you don't do it properly, you can get

	甘噶了，甘就会，出现哩嘢问题	fired. And some employees don’t even mind—especially the younger ones. If they get fired, then they get fired. That’s just how it is. And when employees think like that, these kinds of problems show up.
3. incompatible<br>3.1. Family needs and work demands	Girlfriend doesn’t want him to go on a business trip, affect work mood, depress at work, career opportunities.<br>我女朋友系，佢唔系好愿意自己一个系我屋企住，啱系我唔系度噶情况下，甘假设我又要出差，我就一定要当日来回，甘就系，依家噶，我之前噶出差咧，都系有条件做到当日来回噶，甘未来，有可能会有嘢冇办法当日来回出差甘，甘我到时会出现嘢问题咯… (无法出差)又唔可以话有嘢沮丧，而系有嘢，沮丧可惜？（long pause）啱系会有嘢烦躁咯会，会觉得自己未能够系去，去掌握到嘢，好多嘢啦	My girlfriend… she isn’t really willing to stay at my home by herself—meaning when I’m not there. So if I have to go on a business trip, I definitely have to make it a same-day round trip. That’s how it is right now. My previous business trips were all ones where it was possible to go and come back the same day. But in the future, there might be some trips where a same-day return isn’t possible, and then I’ll run into some problems…<br><br>It’s not exactly that I’d be “depressed,” but more like… a kind of disappointment or regret? (long pause) Like I’d get a bit irritated. I’d feel like I’m not able to… to have control over a lot of things.
4. Work is for self-worth		

5. Family of origin<br>5.1. Financial status<br>5.2. Living together	Has less family responsibility<br>我基本上唔承担家庭责任	I basically don't take on family responsibilities.
6. Conflict with family	比如话返工之前吵完架，甘你返去果朝都唔系好做到嘢	For example, if you argue before going to work, then when you go in that morning you won't really be able to get much done.

Interviewee 3
Female, 24 years old, work at an exhibition company, sales team; fixed salary – 6,500RMB per month, 7-8,000RMB per month after commissions; normally work from 9 AM to 6 PM, when has project, work hour change to 996 (from 9 AM to 9 PM, 6 working day per week); single, no child, living with mom (no job), doesn't have to pay rent and living expense to her mom, hired a housekeeper. (8 significant statements)

1. Organization environment<br>1.1. Work pressure	Increase job distress<br>末位淘汰制跟住会工作压力大…因为依家甘样(covid-19)，佢就有哩个末位淘汰，业绩最差嘅就会炒甘	The "bottom-ranking elimination" system makes work pressure really high… because of the current situation (COVID-19), they use this system where the person with the worst performance/results gets fired.
2. Perceived inequity<br>2.1. At work	Doesn't got pay enough<br>（冇加班费）会成日都好心理唔平衡咯，尤其我地 d 同事都会成日吵，啲话加班加到狗甘，但系又冇话有加班费甘，到最后努力落来又唔系话可以稳到多好多客户，甘 d 钱同埋我地嘅努力唔系成正比嘅咯	(With no overtime pay) you often feel really psychologically unbalanced, especially since my colleagues argue about it all the time. Like, we work overtime like crazy, but there's no overtime pay. In the end, even if you put in the effort, it's not like you can bring in

		that many more clients. So the money and the effort we put in aren't proportional.
3. Incompatible<br>3.1. Work demand and family needs (inflexible/long working hours)	Couldn't help her mother to take care her sick grandparents, doesn't have the time, decrease work motivation 啲好似我侬家公公，佢有病，要住院，甘我阿妈都好辛苦，佢想有个人帮下佢轮流凑下，但（我）都冇哩个时间可以去甘左	For example, my father-in-law is sick and needs to be hospitalized, so my mom is having a really hard time. She wants someone to help her and take turns looking after him, but I (personally) don't have the time to do that.
4. Work is for self	其实工作都系为左我自己	Actually, work is also for myself.
5. Understanding<br>5.1. With parents	屋企人都会理解到，但都会成日对我 shen 咯，啲话我妈脾气最近都唔会话特别好，啲成日都会觉得好无助咯	My family understands, but they still often vent their frustration at me. Like, my mom's temper recently hasn't been especially good—she often feels really helpless.
6. Family of origin<br>6.1. Financial status<br>6.2. Living together<br>6.3. Expectation	Less family responsibility 佢觉得我自己照顾到自己就好了…因为我都冇话真系用我工作嘅钱去辅助家庭啊嘛	She feels that as long as I can take care of myself, that's enough… because I haven't really used the money I earn from work to support the family, you know.
7. Health of families<br>7.1. grandfather	Has to stop working	For example, this time my grandfather was hospitalized and

	就好似今次公公住院要我去帮手，啱话开车去接佢地咯	they wanted me to help—like driving over to pick them up.

Interviewee 4

Female, 28 years old, work at a bank, 9PM to 5AM, 5 working day per week, occasionally work overtime on Saturday, income – 8,000 to 10,000RMB per month; in a relationship, no child, living with parents (still working), grandmother, and a housekeeper/carer, doesn't have to pay rent and living expenses to her parents.

1. Organization environment<br>1.1. unsupportive supervisor	跟住本身我地嘅组长就话会带下我嘅，啱话会话比我听有 d 嘢会教我咯，但系因为佢太多嘢做了，所以好多时候都系直接比嘢我做，跟住我要自己去摸索到底点样做啊，跟住就一直都会，因为好多野你未试过做就好惊做衰，跟住我系果种比较追求完美嘅人，跟住我就成日都会惊自己做唔好啊，有冇地方漏啊，跟住就会一直就会处于比较焦虑嘅状态咯	Originally, our team leader said they would guide me—like telling me things and teaching me. But because they have too much to do, a lot of the time they just hand the work to me directly, and I have to figure out for myself how to do it. So I end up constantly feeling that way—because when there are many things you've never done before, you're really afraid of messing them up. And I'm the kind of person who pursues perfection, so I'm always worried I won't do it well, or that I've missed something. So I'm often stuck in a fairly anxious state.
2. Emotion at work	佢（父母）开心主要系因为佢地见到我开心，因为佢知道我做嘢唔开心，系啊，啱佢地就会觉得我难得哩段时间开心返甘样咯	

3. Work is for self	我稳钱系真系为左稳钱，我系为左自己赚钱，自己通过自己努力去买自己想买嘅嘢	They (my parents) are happy mainly because they see me happy. Because they know I'm not happy at work, so they feel like it's rare that I've been happy again for this period of time.<br><br>I earn money really just to earn money. I'm making money for myself—so that through my own efforts, I can buy the things I want to buy.
4. Family of origin<br>4.1. Financial status<br>4.2. Living together<br>4.3. Expectation	但佢地就开玩笑话，我都唔差哩份工，屋企比你（I）嘅钱都够你洗嘅啦，你稳份轻松 d 嘅工就得啦<br>我依家都仲系爹地妈咪果度，所以好多事情，比如屋企，比如婆婆前排住院啊，跟住，唔舒服啊果 d，果 d 嘅话基本上都系爹地妈咪或者我姨妈系度处理咯，啲话基本上屋企嘅事情，哩种突发嘅事情，都系佢地处理嘅<br>佢地都话反正爹地妈咪唔洗你养，你就做好自己嘅嘢你稳钱自己洗就得嘅了	But they joke and say, "You don't really need this job. The money the family gives you is already enough for you to spend. Just find a more relaxed job."<br><br>I'm still living with my dad and mom right now, so a lot of things—like things at home, like my grandma being hospitalized recently, or being unwell and stuff like that—are basically handled by my dad and mom or my aunt. So essentially, when there are sudden emergencies at home, they're the ones who deal with them.

		They also say, "Anyway, your dad and mom don't need you to support them, so just focus on doing your own things. Earn money and spend it on yourself—that's enough."
5. Incompatible<br>5.1. Work demand and family needs (long working hours)<br>5.2. Work demand and family needs (Inflexible working hours)	(inflexible)如果就我来讲噶话就，我觉得系公司果边咯，因为佢（公司）d 时间太，就是可能有时候会有 d 突发噶事情啊之后叫你返去加工啊之类果 d 咯，如果我屋企冇事就可能冇咩问题噶，或者有时候有事或者已经约左食饭啊甘样噶话就，就会比较麻烦 d 咯，因为你食饭可以推话晒都屋企噶，但工作上面你推唔到噶嘛，甘但系你食饭你推的多噶话，屋企人又可能有意见噶嘛，所以我觉得系工作上面咯，但是又没有办法	If it's just from my point of view, I think it's mainly on the company's side. Because their timing is really… like sometimes there are sudden things that come up and then they ask you to go back and work overtime, that kind of thing. If nothing is happening at home, then it's probably not a big problem. But if something happens, or if you've already arranged to have dinner, then it becomes more troublesome. Because you can postpone dinner, sure—it's a family thing. But with work, you can't really postpone it. But if you postpone dinner too often, your family might have opinions about it. So I feel it's mainly work-related, but there's no way around it.

6. Perceived inequity<br>6.1. At work	但系我觉得基本上，系哩边想话嗜系你付出几多跟住就收获几多哩个系好唔现实噶咯，加班就是加班啊，就没有那么多回报的呀	But I basically feel that the idea of "you get as much as you put in" is very unrealistic. Overtime is just overtime—there isn't that much payoff in return.
7. Supportive parents		
8. (Mutual) understanding<br>8.1. With parents	跟住所以我自己去同佢地讲，佢地都会觉得差唔多就得啦，你就是身体比较重要嘛，跟住就后尾就，果阵时就会觉得，点解爹地妈咪唔理解我，嗜我就觉得爹地妈咪点解帮唔到我手，就觉得好委屈甘，自己系度捻就觉得情绪好唔好。<br>因为这个世界上还是有代沟这个东西的存在的，他们（父母）再支持你他们也不懂你这一代人的想法的，所以，唉，鸡同鸭讲。	So when I talk to them about it, they feel that "as long as it's roughly okay, that's enough"—that your health is more important. And then later on, at that time, I would feel: why don't my dad and mom understand me? I felt like, why can't they help me? I'd feel really wronged, and when I kept thinking about it by myself, my mood would get really bad.<br><br>Because in this world, there's still such a thing as a generation gap. Even if they support you, they still don't understand how people of your generation think. So… sigh, it's like talking past each other.
9. Abreaction	因为其实我发现好多人都系噶，嗜系点解 d 人，嗜系屋企脾气就好大，其实真系因为我地发脾气都会	Because I've noticed that a lot of people are like this. Like, why do some people have such a bad

	好习惯性对亲近嘅人发脾气嘅嘛，甘其实你系公司定单位受到嘅委屈啊，甘你堆积左一日嘅情绪，跟住你返到屋企就好自然甘，就是压抑不住就不小心就发泄出来了，因为白天已经是带着面具了	temper at home? It's actually because when we lose our temper, we tend to do it toward the people closest to us. So if you've suffered grievances at your company or workplace, you've built up a whole day of emotions, and then when you get home it just naturally happens—you can't hold it in, and you accidentally vent it out. Because during the day, you've already been wearing a mask.
10. Single child<br>Take care parents	因为我系独生，所以我就会觉得我老豆老母如果我老左之后，我一个人照唔照顾的来咧？之后我就会开始慌，开始焦虑，又会觉得如果突然之间有 d 咩需要用到钱嘅时候，我又拿唔出果笔钱喔，跟住我就又开始焦虑，跟住捻下捻下，算啦，我都系努力做嘢啦，我都系唔好辞职了，系啊，虽然我系觉得同我嘅付出唔系甘成正比嘅，但其实，就系其他朋友度听到就会觉得，要稳份甘样嘅工，都几难嘅啦，出边都有出边嘅辛苦，或者出边可能仲衰添，所以就挨一挨甘啦	Because I'm an only child, I worry: when my parents get older, can I take care of them all by myself? Then I start to panic and get anxious. I also think, if something suddenly happens where money is needed and I can't come up with that amount, then I get anxious again. And after thinking and thinking, I'm like, forget it—I'll just keep working hard, I won't quit. Yeah, even though I feel what I get isn't really proportional to what I put in, when I hear from other friends, I feel that getting a job like this is actually quite

		difficult. Outside has its own hardships too, and it might even be worse out there. So I'll just endure it for now.

Interviewee 5

Male, 34 years old, became an insurance broker recently (a year ago), no fixed income, income level depends on the commission, unwilling to provide income; second marriage, married 2 years, one boy, one and 8 months old, living with wife, child, his parents or his wife's parents (e.g. stay at his parent's house this week then move to his wife's parent's house next week), parents are all retired, wife has full time job, doesn't have to pay rent and live expense to his parents or his wife's parents. (16 significant statements)

1. Supportive parent	因为要父母去照顾小朋友，甘我地白天年轻噶需要去工作甘白天时间我地冇办法照顾小朋友噶嘛，甘我地最多都系周末去照顾.<br>家庭噶开支啊或者咩嘢甘肯定会将个压力转移到父母身上，会有甘噶情况	Because we need our parents to take care of the kids. We young people have to work during the day, so we don't have a way to look after the children in daytime hours. At most, we can take care of them on weekends.<br><br>And household expenses and things like that will definitely shift the pressure onto the parents—this kind of situation can happen.
2. Income	影响到家庭其实可能目前来讲收入未系算系一个好稳定噶时期.<br>我哩个家庭来讲，如果你话能够真真正正解决左大部分经济上噶嘢甘可能都解决到 7，8 成（问题）啦.	It affects the family because, at the moment, our income still isn't at a very stable stage.<br><br>For my family, if you could truly solve most of the financial issues, then you'd probably

		solve about 70–80% of the problems.
3. Interrelationship<br>3.1. With spouse	甘肯定都会有噶，甘哩方面其实我觉得更加系需要，个另一半去支持啦，然后父母噶理解啦甘，甘系要靠哩方面去承载返哩份压力咯.<br>我就可能会少关注左另一半噶感受咯，相对来讲会，可能我会，返来好多时间睇下手机啊玩下游戏啊忽略左佢噶感受咯.	Yes, definitely. In this area, I think it's even more important to have support from your partner, and understanding from your parents. You have to rely on those things to help carry that pressure.<br><br>I might pay less attention to my partner's feelings. Relatively speaking, when I come home I might spend a lot of time looking at my phone or playing games, and end up neglecting how she feels.
4. Children (focus on work become focus on family?)<br>4.1. Age of Child<br>4.2. Education	其实点讲咧，你话冇动力去做家务咧，其实反而，因为有小朋友出左世，就我个人来讲啦，就我自己来讲系有动力去做家务噶，甘但系你话会唔会系令到人好主动积极咧，亦都唔会，甘你睇到或者家人要求到噶甘你都要去做<br>因为系照顾方面其实主要都系 4 位老人家噶白天时间为主啦，甘晚上噶话就我地能够返来噶话都系我去照顾咯，因为始终依家来讲都系叫做每边都有 3 到 4 个人	How should I put it… if you're talking about not having the motivation to do housework, actually, on the contrary—because we have a child now—personally, for me, I do have the motivation to do housework. But will it make someone super proactive and enthusiastic? Not really. If you see it needs doing, or if your family asks you to, then you have to do it.

	去照顾一个小朋友，人数方面系够噶，人数方面我觉得系够噶，甘但系你话系其他方面嘅话，各方面嘅知识啊咩嘢来讲可能就会需要我地年轻一辈更加去更新更加多嘅知识咯，同埋唔可以用旧有嘅方法去教育咯，要 d 新一 d 嘅方式去教育，避免左话好似上一代甘一 d 好填鸭式啊，好管教似嘅方式去教育，但整体来讲咧，照顾嘅责任就互相去平分返咯，因为依家哩个阶段就还好啦，甘可能去到下一个阶段，你话幼儿园啊，或者读书嘅时候可能教嘅责任可能会落返去我地后生嘅身上咯	As for childcare, during the day it's mainly handled by the four grandparents. At night, if we're able to come back, then I'm the one who takes care of the child. Because as things are now, it's like each side has three to four people looking after one child—so in terms of manpower, it's enough. I think the number of people is enough.<br><br>But in other areas—like knowledge and so on—we, the younger generation, probably need to keep updating ourselves more. And we can't use the old methods to educate the child; we need newer ways, to avoid the older generation's kind of "cramming" and overly strict, controlling style of education. But overall, the caregiving responsibilities are shared and divided between us. At this stage it's still okay. But maybe at the next stage—like kindergarten, or when the child starts school—the responsibility for teaching might fall back onto us younger people.
5. Family of origin<br>5.1. Financial status<br>5.2. Living together	妈咪果边嘅退休嘅经济都算比较高…系生活上啊，琐碎嘅地方我妈咪	My mom's side has a relatively good retirement income. For everyday life and the

	会比咯，甘其实等于我地唔洗话专门交钱比佢地，甘佢地又买生活上嘅所需啦，衣食住行各方面，甘其实就相当于系补贴左我地啦	little day-to-day expenses, my mom will cover them. So it’s like we don’t have to specifically give them money—because they’ll buy the daily necessities for living, everything from food, clothing, housing, and transport. So in effect, they’re subsidizing us.
6. The perceived of need<br>6.1. Change of the society	因为依家社会发展的太快了，你需要积累嘅嘢好多，就算你依家嘅工作，即使唔系我依家嘅状态，就系，即使工作收入好稳定好高都好你都需要不断去学习不断去更新你嘅知识，甘所以你肯定系照顾家庭方面肯定嘅时间会小咯	Because society is developing too fast now, there’s a lot you need to build up. Even with your current job—even if you’re not in my situation—even if your income is stable and high, you still need to keep learning and constantly updating your knowledge. So definitely, the time you can spend taking care of the family will be less.
7. Take care parents	你话系好担心我又觉得唔至于，但你话完全放弃唔捻甘亦都唔会...你话系好担心我又觉得唔至于，但你话完全放弃唔捻甘亦都唔会	I wouldn’t say I’m *very* worried, but it’s not like I would completely give up and not think about it either… I wouldn’t say I’m *very* worried, but it’s not like I would completely let it go and not think about it either.
8. Flexible working hours	我觉得目前来讲系基本协调到啦，因为我基本上都会尽量将个时间，调整咯，尽可能以白头为主啦	I think for now it’s basically coordinated. Because I usually try my best to adjust my time, and as much as possible, prioritize the daytime.

Interviewee 6
Male, 35 years old, insurance broker, no fixed income, commission income – at least 10,0000RMB per year, flexible working hours; married 4 years, no child, living with parents and wife, doesn't have to pay rent, but will pay live expense, parents retired, wife has fulltime job. (19 significant statements)

1. Income	我老婆就唔系甘中意，但佢亦都冇话去叫我唔好做咯，只系唔中意我做哩个行业啫…（因为）收入唔够稳定	My wife doesn't really like it, but she also hasn't told me not to do it. She just doesn't like me working in this industry… because the income isn't stable enough.
2. Flexible working hours	冇甘固定嘅，基本上就系 1 到 5 系上午返公司啫系报个道啊，开个早会，跟住其他时间就系自由安排	It's not that fixed. Basically, Monday to Friday I go to the company in the morning just to check in and attend the morning meeting, and then the rest of the time is arranged freely.
3. Work is for family	肯定系为左增加收入咯，第二肯定系为左屋企人去奋斗咯，啫系对屋企人嘅一个责任心来嘅咯	Definitely to increase income. And secondly, definitely to work hard for the family—basically, it comes from a sense of responsibility toward the family.
4. Health of families<br>4.1. Parents	因为我父亲系大病过嘅，所以依家对佢嘅照顾会比较多 d…当时嘅心情肯定就系烦嘅啦，因为又要工作稳钱，屋企人又病嘅话，啫系又要考虑到忙完之后返去照顾佢地啊，都会系度捻	Because my father had a serious illness before, so now I have to take care of him more.<br><br>At that time, I was definitely feeling upset and stressed, because I had to work to earn

	佢地噶身体状况系可以几时康复甘样咯<br>应该系前段时间我父母都一齐病左噶时候，哩个时候我系完全放低晒手头噶工作去照顾佢地 2 个噶…呃，之前，甘 2 个病，都要照顾噶，甘都冇得工作噶了	money, and if a family member is sick, you also have to think about going back to take care of them after you're done with work. I would keep thinking about their health condition—like when they might recover.<br><br>It should have been some time ago when both my parents got sick at the same time. At that point I completely put aside all the work I had on hand to take care of the two of them… yeah, before—when both of them were sick and needed care, I couldn't work at all.
5. Take care parents	就系经济，因为随着佢身体如果系出现问题，甘果时一个系医疗，第二个系我要照顾，甘我工作时间就会减少，甘我工作时间减少收入就肯定会降低，甘哩个就系我噶经济压力啦	It's mainly financial. Because if his health has problems, then first there are medical expenses, and second I have to take care of him—so my working hours will decrease. And if my working hours decrease, my income will definitely drop. So that's my financial pressure.
6. Sense of family	我觉得，如果系家人病导致要放下工作去照顾噶，我觉得哩种唔系冲突咯，系对家人一个必须要做噶野咯，系一个责任来咯	I think if a family member is sick and it makes you put work aside to take care of them, I don't see that as a "conflict." It's something you must do

		for your family—it's a responsibility.
7. Work is for family	啲话工作上希望能够取得成功，哩个我觉得系人人都想嘅嘛，甘所以选择咩工作肯定系选择自己中意嘅工作咯，但同时亦可以啲哩个中意嘅工作可以创造收入而唔会话咩野嘅，啲造成因为中意而影响家庭嘅（收入）	In other words, you hope to succeed at work—everyone wants that, right? So when choosing what job to do, you'd definitely choose something you like. But at the same time, you'd want that job you like to be able to generate income too, and not end up in a situation where, because you like it, it affects the family's income.
8. Neglect	啲捻开 d 咯，好似就系甘样，我头先都讲嘅如果发生哩 d 事嘅时候，系每个人都会经历嘅事情来嘅了，唔系话就得我一个甘特殊嘅	Just think of it more openly—something like that. Like I said earlier, when these things happen, it's something everyone will go through. It's not like I'm the only one who's so "special."

Interviewee 7

Female, 39 years old, university English teacher and owner of an English learning studio; only works three days per week (university), income – 10,000RMB per month (university only); married 14 years, living with her husband and son, husband has job, son is 12 years old, hired an hourly housekeeper for cleaning and cooking. (24 significant statements)

1. Working hours	我们这个是不用做班的，就是我有课我就去，没有课我就在家，所以我们没有固定的上班时间，一周大概，一周会去 3 天学校，因为一周只有 3 天有课.<br>因为今年我自己做了一个工作室，就是我做了一个	We don't really have fixed office hours. If I have classes, I go in; if I don't have classes, I stay at home. So we don't have a set working schedule. I go to the school about three days a week, because there are only

	英语教学的工作室，所以相对来说就是要比平时再忙一点.	classes on three days each week.<br><br>Also, this year I set up my own studio—an English teaching studio—so comparatively speaking, I'm a bit busier than usual.
2. Child<br>2.1. Education<br>2.2. Number of children	刚好今年我们家闹闹，就是我们孩子，又是 6 年级，对他来说是小学里面最重要的一年嘛，所以呢，那可能我时间上的分配就会比较那个一点.<br>就是正常上补习班吧，因为现在都在上，特别是我们江苏，非常重视教育，所以你如果课外没有 3-4 个补习班，都不正常.<br>因为第一接送，要接送嘛，那接送的话就是会占掉我一部分的时间，可能会让我有一点焦虑，因为他上这个东西他要吸收对不对？所以我会督促他去做一些作业啊督促他去做一些这些方面的作业，检查，比如说我还要跟他讨论，他学完这些东西以后，还要跟老师去沟通，所以将这些都无形当中都是压力.<br>不会觉得压力很大，因为我就一个孩子嘛，所以我也不会的有很（大压力）.	This year things at home have been a bit hectic—our child is in 6th grade, and for him it's the most important year in primary school. So my time allocation will be a bit more… tight/affected.<br><br>It's basically the normal thing of attending extra tutoring classes, because he's taking them now. Especially in Jiangsu, education is taken very seriously, so if you don't have 3–4 extracurricular tutoring classes, it's considered "not normal."<br><br>First, there's the pick-up and drop-off—you have to take him and pick him up—so that takes up some of my time. It can make me a bit anxious, because he has to absorb what

		he's learning, right? So I push him to do homework in those areas, check his work, and for example I also have to discuss things with him. After he finishes learning those things, I also need to communicate with the teacher. All of this, invisibly, becomes pressure.<br><br>But I don't feel the pressure is very big, because I only have one child, so it's not *that* much pressure.
3. Sense of family<br>3.1. Gender<br>3.2. Caregiving	我比较忙，然后我爱人，就是我老公还是希望我多在家里面，辅导闹闹，但是我可能，要匀（拿）掉一部分的时间出去.<br>没。。。有，没有，因为我觉得这就是我的生活的一个部分了已经是，已经习惯了。。因为从小到大就是我来负责他（小孩）的生活起居.<br>父母对啊，就是个人自己的父母都是个人自己的责任嘛.<br>我。。我好像已经没有感受了（笑），我好像也已经觉得这就是我的责任了	I'm relatively busy, and my partner—my husband—still hopes I can be at home more to tutor Naonao, but I might have to set aside part of my time to go out.<br><br>No… not really. Because I feel this is already part of my life, and I'm used to it. Since he was little, I've been the one responsible for his daily life and routines.<br><br>Parents—yes, everyone's own parents are their own responsibility.

		I… I feel like I don't really have any particular feelings about it anymore (laughs). I've also come to feel that this is just my responsibility.
4. Job tenure	就是刚开始的时候会有一点影响，因为那个时候就是事情很多，比如说闹闹这个时候正要我辅导他做个作业，我要给他在安排一些其他的事情做的时候，这个时候呢我又有课，我要去工作室上课或者说我要去工作室开会，那不就冲突了嘛，这个时间，我就不能跟闹闹在一起	At the very beginning it did affect things a bit, because at that time there were a lot of things going on. For example, when Naonao needed me to tutor him with homework, or when I needed to arrange other tasks for him to do—if at that moment I also had a class, and I had to go to the studio to teach or go to the studio for a meeting, then it would be a conflict. During that time, I wouldn't be able to be with Naonao.
5. Abreaction (发泄在家人身上)	我会生气（笑），对，我会生气，我会跟闹闹生气，因为我会觉得他那么大了还不可以好好管理自己，就自己不可以自己管理好自己的时间，但是有一段时间我是很压抑的，就是觉得好烦躁啊，就是觉得很忙很忙，就都会有一些这样的情感	I would get angry (laughs). Yes, I'd get angry—I'd get angry with Naonao, because I'd feel that he's already so big and still can't manage himself properly, that he can't manage his time well on his own.<br><br>And for a period of time I was really repressed—feeling very irritated, feeling like I was so busy, so

		busy—so I did have emotions like that.
6. Health of families<br>6.1. Children<br>6.2. parent	比如说啊，如果他身体不舒服啊，那我肯定是把工作所有的事情都暂停下来带他去看病，呃，然后，其他的如果是因为照顾小孩，好像还没有特别那样的例子<br>因为他们现在都还身体健康，但将来年级大了以后肯定会比如这里那里都需要人照顾嘛.	For example, if he isn't feeling well, then I'll definitely pause all my work and take him to see a doctor. And other than that—cases where I had to stop work specifically because of taking care of the child—there haven't really been any especially notable examples yet.<br><br>Because they (the parents) are still healthy right now, but when they get older in the future, of course they'll probably need people to look after them here and there.
7. Parents support	比如说我要是买房子啊，买房子，还有我有的时候小孩子忙不过来了，如果我要出差啊或者是我老公要出差，我要上课，那忙不过来的时候我就会把我父母叫过来帮忙	For example, if I'm buying a house—buying a home—or sometimes when I can't manage things with the child: if I need to go on a business trip, or my husband needs to go on a business trip, and I have classes to teach, then when it's too much to handle, I'll call my parents over to help.

8. Single child	因为我是独生子女嘛，所以将来我父母肯定是要跟着我一起生活的.<br>因为这是一个责任嘛，那这个责任就是等你到了一个上有老下有小的这种状态时候的一种心理的压力吧.	Because I'm an only child, in the future my parents will definitely need to live with me.<br><br>Because this is a responsibility. And that responsibility is a kind of psychological pressure you feel when you reach that stage of "elderly parents above and young children below."
9. Work for family	因为他对我提供收入，所以这个也是为了家庭咯，就是他可以满足我家庭的需求，但是我工作不是单纯为乐给家人一份补贴，不是	Because it provides me with income, so this is also for the family—it can meet my family's needs. But I don't work simply just to give my family some extra financial support; it's not just that.
10. Family support	就我在英国嘛，对，因为我一个人照顾他嘛，我一个人照顾他后来我妈妈来了，那个是后半年的事情，我一开始我一直都没有觉得我自己很辛苦，因为我在国外不用工作，所以我本来觉得我是没那么苦的，但是我后来回来以后，再这么一对比，然后我发现还是，其实我当时其实是承受了很大的压力，因为我一个人带孩子，如果出现了任何的问题，其实是很那个的嘛，就是很焦头烂额的事情嘛，所以回来以后才发现我其实当时心理压力是很	When I was in the UK—yes—I was taking care of him on my own. Later my mom came, but that was in the second half of the year. At the beginning, I never felt that I was working very hard, because I didn't need to work abroad, so I thought it wasn't that tough. But after I came back and compared things, I realized that actually I was under a lot of pressure back then. Because when you're raising a child alone, if anything goes wrong,

	大的，但是当时我倒没觉得，等回来一对比，还是家里有安全感，很舒服	it can be really overwhelming—really stressful and chaotic. So it was only after coming back that I realized my psychological pressure at that time was actually very high. But back then I didn't really feel it; it was only after I came back and compared that I felt it. At home you have a sense of security—it's comfortable.
11. Nature of the job	因为我们这个工作相对比较单纯，也没有天天去，你上完课就走，你上课了你才在那，甚至我们都不用跟其他同事接触，比如说我上课就直接进教室了，我上完课我就直接回家了	Because our job is relatively straightforward, and we don't have to go in every day. You teach your class and then you leave—you're only there when you have classes. We don't even need to interact with other colleagues much. For example, when I have a class, I go straight into the classroom; when I finish, I go straight home.

Interviewee 8
Female, 35 years old, financial planner/team leader, flexible working hours, no fixed salary, only commission, income – at least 20,000RMB per month; single, 2 sons, 3 years old and 1 years and 8 months old, living with parents and her younger son, parents retired, will pay rent and live expense to her parents, hired an hourly housekeeper for cleaning. (22 significant statements)

1. Nature of the job	甘可能你话困难，可能最困难噶可能系团队噶流传咯…招返来噶人后来又流失	So if you're talking about difficulties, maybe the biggest

	左噶，甘其实就系维护团队噶一个稳定，哩一个系一个比较难噶嘢咯	difficulty is staff turnover within the team… people you recruit later leave again. So maintaining a stable team is something that's relatively hard.
2. Gender	当中其实依家回顾返最主要噶问题就系，可能我返公司返的少，我之前生小朋友休产假休左好长时间，所以哩样嘢都算系一种挑战咯，啱你话完全咧，对于女性来噶讲咧，你生小朋友对工作一 d 影响都冇系比较难噶，啱话，即使你系一 d 事业性噶单位啊，事业单位可能会好 d，但多多少少咧，你离开一段时间咧，肯定公司 d 人事啊，对你肯定就冇甘重用噶啦，甘亦都有可能你离开哩段时间，好多机会就可能就会比左人地，甘哩样嘢无可避免噶	Looking back now, the main issue is probably that I don't go to the office much. Also, when I had a child, I took maternity leave for a long time, so that counts as a kind of challenge too.<br><br>I mean, for women, it's quite hard to say that having a child has absolutely no impact on work at all. Even if you work in a public institution (which might be better), more or less, if you're away for a period of time, the company's HR/management will definitely stop relying on you as much. And it's also possible that while you're away, many opportunities will be given to other people. That's unavoidable.
3. Family relationship<br>3.1. With spouse	其实我前夫佢系好唔认同我噶工作噶，甘佢觉得我令到佢好 shame 噶哩个工作，但系之前咧，老师噶	Actually, my ex-husband really didn't agree with my job. He felt that my job made him feel ashamed. But before that, when I

	工作系会令到佢好 proud 噶.<br>佢地会觉得话你系咪应该要去做老师会好 d 咧？啫佢地都会有一个自我否定噶一个过程，甘包括我自己可能都会经历过有一 d 自我否定咯.<br>如果哩个丈夫佢对你佢系一个支持噶态度噶，同埋大家系达成左一个好好噶共识噶，甘其实佢会各方面去支持你咧，就唔会存在有因为哩个工作带来噶矛盾噶，因为任何困难咧都系自己捻出来噶，就只需要大家去达成个共识咧，其实所有困难都系可以一齐去面对.<br>亦都唔洗去顾虑好似以前前夫甘，佢会抛好多难题过来比我，要拆弹，成日要拆，我真系觉得好累.	worked as a teacher, it made him feel very proud.<br><br>They would think, “Shouldn’t you go back to being a teacher? Wouldn’t that be better?” There’s a process of self-doubt, and that includes me too—I probably went through some self-doubt as well.<br><br>If your husband has a supportive attitude toward you, and you’ve reached a good mutual understanding, then he’ll support you in all aspects, and there won’t be conflicts caused by the job. Because a lot of “difficulties” are things people imagine up in their heads—if you just reach a consensus, you can face everything together.
4. Health of family<br>4.1. parents	就比如话好似我妈之前佢唔舒服要做手术，甘其实我都行开左 2 个月噶了.<br>可能影响就影响在于我冇再去见新噶客户噶果段时间，同埋冇去，呃，去招募全新噶团队成员啊，冇时间，因为个 2 个月，主	For example, when my mom wasn’t feeling well and needed surgery, I was basically away for two months.<br><br>The impact was that during that period I

	要就，啲就系主要就维护返 d 旧客户啊甘样样咯	didn't go meet new clients, and I didn't have time to recruit brand-new team members either. During those two months, I mainly just maintained the existing clients.
5. Flexible working hours	啲比如话我今日，寻晚见客见到好累了唔想返，甘我今日就训晏 d 咯，甘你咪晏 d 返咯，虽然你冇，冇计你噶 attendance，但你返到去都会见到你 d 组员噶嘛，都会，起码大家会见到你咯	For example, if today—say I met clients until very late last night and I'm exhausted and don't want to go in—then today I'll sleep a bit later, and I'll go back to the office later. Even though they don't really count your attendance, when you go back you'll still see your team members—at least everyone gets to see you.
6. Children	因为好现实啦，我依家有 2 个仔，虽然另外一个仔依家未必系咪判到比我我唔知，但系，呃，但系我起码我自己要预留左一 d，比如佢地第日噶教育金啦，佢地噶创业金啦，婚嫁金啦，甘哩 d 其实我都要去为之而努力噶嘛，无论佢地未来会唔会去用到我哩笔钱，甘我肯定都要为佢地准备住噶嘛.	Because realistically speaking, I now have two sons. Although I don't know yet whether the other son will be awarded to me (in terms of custody) or not, but… at the very least, I need to set aside some things—like their future education fund, startup fund, marriage fund. These are things I need to work hard for. No matter whether they'll actually use this money in the future, I

		definitely need to prepare it for them.
7. Parents support	其实屋企系比左我好大帮助噶，甘例如话，依家小朋友依家仲好细啊嘛，甘佢就系，又未识讲野，所以我就唔系甘愿意带佢去幼儿园咯，因为我好惊佢唔知会唔会受到 d 咩对待，因为依家太多哩 d 新闻啦嘛，甘所以就好多得我父母去帮我带小朋友咯，啱系我唔系屋企噶哩段时间.	Actually, my family has helped me a lot. For example, the child is still very young right now and can't really talk yet, so I'm not that willing to send him to kindergarten. I'm really afraid I won't know whether he might be treated badly somehow, because there are too many news stories like that nowadays. So I really have to thank my parents for helping me look after the child when I'm not at home
8. Take care parents	其实点讲咧，呐，佢地依家都仲 ok，5，60 岁，甘咧，最担心咧就系当佢地年级再大 d 噶时候.<br>系啊系啊，肯定系责任来噶，甘个个都会，啱点讲咧？每个人可能，啱人咧，都有趋利避坏噶，甘但系有 d 事情咧，系唔轮到你去逃避噶嘛.	How should I put it… right now they're still okay—about fifty or sixty years old. What I worry about most is when they get older.<br><br>Yes, yes, it's definitely a responsibility. Everyone… how do I say it? People tend to seek benefits and avoid harm, but there are some things you simply can't run away from—it's not up to you to avoid them.

9. Family of origin<br>9.1. Financial statue	甘我觉得钱系好紧要咯，所以依家要努力赚钱，甘虽然我父母咧，佢地都唔会话比我好大噶压力，而且咧，佢地都有唔错噶收入，甘但系，我希望自己都可以做的更加好 d 咯<br>啱如果屋企唔系有个唔错噶一个经济支持噶话，我捻我都，好难会做到依家哩份工啦，啱因为，哩 d 开头，肯定都系唔会甘容易噶嘛	I think money is very important, so right now I need to work hard to earn money. Even though my parents don’t put a lot of pressure on me, and they also have a pretty good income, I still hope I can do better myself.<br><br>If my family didn’t have decent financial support, I think it would be hard for me to do the job I’m doing now. Because at the beginning, it definitely wouldn’t be that easy.
10. Single child	（一胎政策）系啊，甘同时都害死我咯（笑），啱如果有个人同我一齐分担下，系好唔一样，	(The one-child policy) Yeah—and it’s also “killing” me (laughs). If there were someone to share the burden with me, it would be completely different.
11. Income	其实，当然哩个系好重要啦，甘同埋仲有自己会有积蓄噶嘛，甘，所以就，(家庭责任导致无法工作)都唔会太担心噶，因为都知道系暂时噶嘛	Of course, that’s very important. And also, you’ll have your own savings, right? So because of that, if family responsibilities make it impossible to work, I wouldn’t be too worried—because I know it’s only temporary.
12. Neglect	其实冇（调节过来）噶，都系因为，嗯，生活仲系	Not really that I “adjusted”—it’s just because… life still has

	要过噶嘛，甘，呃，喊完之后咪就都要继续生活噶（苦笑），甘所以好就好在经济上我系冇咩压力咯，所以系甘样样噶大前提下一切都好说话我觉得，啱一切都系叫做无病呻吟	to go on, right? So after you've cried, you still have to keep living (wry smile). So the good thing is that I don't really have much financial pressure. Under that big premise, I feel everything is easier to talk about—otherwise it's basically just "complaining for no reason" (like whining when you're not actually facing serious hardship).
13. Work is for family	其实除左系为左自己啦，当然，同埋为左自己噶屋企啦都系，其实，如果系做左一 d 对社会有价值噶野咧，系会更加开心咯 ... 啱如果当哩 2 件事可以同时实验噶时候咧，甘就系最开心噶时候啦 ...（笑），甘当然系责任就行先啦	Actually, besides doing it for myself—and of course also for my family—if I can do something that has value to society, I'd be even happier. If those two things can happen at the same time, then that would be the happiest time (laughs). But of course, responsibility comes first.

Interviewee 9

Female, 26 years old, work at primary school, working hours from 7:30AM to 6PM, income – between 10,000 to 20,000RMB; single, no child, living with parents, parents still working, doesn't have to pay rent and live expense to her parents

1. Nature of the job	正常，（上级领导）没有什么（对我的）帮助，因为我们(我和我的上级领导)，呃，我们的工作性质比较不一样...就可能我对着下级的时间比我对上级的时间多很	Normally, my (superior/leader) doesn't really help me with anything, because the nature of our work is quite different… I probably spend much more time dealing with

	多啊，就是我，我的领导，日常是不太会管到我们的	my subordinates than with my superior. My leader doesn't really manage us much on a day-to-day basis.
2. Family of origin<br>2.1. Financial statue<br>2.2. Single child	是的（照顾父母是自己的责任）… 不会（担心）…因为他们自己也有能力啊…而且我们家有兄弟姐妹啊	Yes (taking care of parents is my own responsibility)… I'm not worried… because they're capable themselves… and also, in our family we have siblings.
3. Work is for self		

Interviewee 10

Female, 38 years old, restaurant owner, small business, only has two employees, long working hours, 8AM to 9PM, six and half days per week; married 5 years, has a 5 years old daughter, living with her mon, big sister and her daughter, all family members that living together have no job, sometimes (during working days) live with her employees in a rental apartment which close to her workplace (around 30mins round trip). (26 significant statements)

1. Nature of the job	餐饮业除左肉啊菜啊，你知道中餐嘅啦，煎炒焖焗炖咩嘢都有嘅嘛… 2 睇啦，其实点讲好咧，其实好大嘅影响系冇嘅，因为我本身嘅爱好系哩一方面，第二方面嘅话咧就系，呃，我哩个人咧比较中意，啱人地开心我就会开心果种人咯，所以人地食得开心嘅时候咧，我就会开心，甘就因为哩 2 个主要嘅原因咧，我唔会话好烦躁，甘你话有时候烦躁系咩咧？就系比如客户会有 d 无理嘅要求嘅时候，或者系客户嘅要求，已经系超过左我地可以承受嘅范围里面嘅时候，我就会觉得烦躁咯	In the food and beverage industry, besides meat and vegetables—you know Chinese food: stir-frying, pan-frying, braising, deep-frying, stewing, all kinds of cooking methods…<br><br>It depends. Honestly, how should I put it—there isn't a huge impact, because first, this is my hobby, and second, I'm the kind of person who feels happy when others are happy. So when customers eat happily, I feel happy too. Because of these

		two main reasons, I wouldn't say I get very irritated.<br><br>When do I get irritated? For example, when customers make unreasonable demands, or when their requests go beyond what we can reasonably handle—then I feel irritated.
2. Incompatible<br>2.1. Work demand and family needs	啱系家庭上面噶话咧，就会相对来讲比较困难，因为我唔可能兼顾的到啦，你时间就系甘样样啦	As for the family side, it's relatively more difficult, because it's impossible for me to take care of everything at the same time—my time is limited like that.
3. Income	甘冇办法噶喔，唔通我系天河买间屋啊？我冇钱啊（笑）<br>你明唔明白啊，对于冇钱噶人，系经济上面噶资助已经系最，最有力同埋最实在噶资助啦已经系，对于穷人，如果你系一个有钱人噶话你永远想象唔到穷系咩状况，明白吗？因为你唔需要担心钱，大家噶位置唔同<br>其实我好想有其他发泄方式噶，例如拿钱去买野啊，疯狂购物啊，但我觉得唔允许咯，算啦，我将哩个方式，ignore 左佢了已经	There's no way around it—what, am I supposed to buy a flat in Tianhe? I don't have money (laughs).<br><br>Do you understand? For people who don't have money, financial support is already the strongest and most practical kind of support. For poor people—if you're rich, you'll never be able to imagine what poverty is like, understand? Because you don't need to worry about money. We're in different positions.

		Actually, I really wish I had other ways to vent—like using money to buy things, going on a crazy shopping spree—but I feel I can't allow myself to do that. Forget it. I've already ignored that way of dealing with it.
4. Child<br>4.1. Age of child	最冲突嘅话，你知我有个 5 岁嘅女啦，甘我系，啱好难照顾到佢，我唔能够话接送佢上课啊同埋放学啊果 d.<br>唔可能啊，因为朝早，佢翻学，佢翻学系 8 点钟，然后 8 点钟我已经要做嘢了，甘下昼佢系 4 点钟翻学，然后 4 点钟翻学我仲系做紧野，甘我点样样咧？.<br>三岁佢比你带来压力嘅野可能会系佢嘅身体啊，会容易多 d 小打小闹嘅病痛啊甘，但系三岁打后啊，依家啊，或者将来啊，佢带比你压力会越来越多系咩野咧，佢嘅读书啊，比如补课啊，你自己都经历过哩 d 阶段啦系咪啊？啱佢会，我会担心佢哩一方面嘅野咯，甘其实唔会因为佢嘅年龄增长而少左嘅，只会系佢，接触嘅野越来越多，你要捻嘅野会越来越多.	The biggest conflict is that, you know I have a 5-year-old daughter. It's really hard for me to take care of her—I can't do things like taking her to school and picking her up.<br><br>It's impossible, because in the morning she goes to school at 8 o'clock, and by 8 I already have to be working. Then in the afternoon she finishes school at 4 o'clock, but at 4 I'm still working. So what can I do?<br><br>When a child is three, the pressure they bring might be more about their health—like they get small illnesses and minor issues more often. But after three, now or in the future, what kind of pressure will they bring? More and more it's about

		their schooling—like extra tutoring classes. You’ve been through that stage yourself, right? So I worry about that side of things. Actually, it won’t decrease as they get older; it will only be that they’re exposed to more things, and there will be more things you have to think about
5. Guilty<br>5.1. Child<br>5.2. parents	细路仔始终都系年级细嘛，我都想多 d 时间陪伴佢嘛系咪先，哩个好正常噶，但系我冇办法做到哩一点咯.<br>除左有个细路仔之外，甘我妈年纪都大了嘛，我妈都 80 岁了，甘系米我都希望可以多 d 留系佢身边咧？甘哩一点我都系同样道理，我系做唔到噶嘛系咪？<br>比方讲，我叫我家姐帮手去照顾啊，但你要明白到，呃，哩个责任，唔能够完全系推到去佢噶身上面噶嘛系咪啊？毕竟个女仲系我噶女，而阿妈就系大家都有份噶阿妈	Kids are still young after all, and of course I want to spend more time with her, right? That’s totally normal. But I have no way to do that.<br><br>Besides having a child, my mom is also getting old—she’s already 80. So of course I also hope I can stay by her side more. But it’s the same logic: I can’t do that either, right?<br><br>For example, I can ask my older sister to help take care of them, but you have to understand that this responsibility can’t be completely pushed onto her, right? After all, my daughter is still my daughter, and our mom is a mom that we all share responsibility for.

6. Single Child	我就只能够叫我家姐唔好工作咯…甘我家姐唔工作噶话咧，系啊，甘我家姐住埋一齐噶嘛，甘佢唔工作噶话就可以帮我照顾我阿妈啦，可以帮我照顾我阿妈啦，可以帮我照顾我细路啦，带佢上落课啦，哩个系最主要噶弥补噶方式，因为冇办法啦，如果佢都工作埋噶话，甘屋企冇办法噶，冇可能做的到噶事情	I can only tell my older sister not to work… If my sister doesn't work—yeah—since she lives together with us, then if she doesn't work she can help me take care of my mom, help take care of my mom, and help take care of my child—like taking her to and from classes. That's the main way to make up for it, because there's no other way. If she also works, then there's nothing we can do at home—those things would be impossible to manage.
7. Family relationship<br>7.1. With spouse	老公哩个名词我真系唔想用系佢身上，因为佢冇一个，哩个家庭上面噶责任感咯，呃，哩个令到我系好唔想提到噶一个话题，因为，点讲好咧？呃，老公哩个名词只系作用于我有哩个女啦，然之后咧哩个女读书，然之后系户口啊咩上面噶话咧需要到，同埋我唔想话太过伤害我老公，啲话唔想太过伤害我个女，啲话甘细个就冇左咩父爱唔父爱噶野，因为我觉得，我自己大人可以承受噶野就算啦，因为佢，佢有佢自己噶追求，我唔系讲噶女啊，啲个老公啊下，个 husband 啊下，佢有佢自己噶追求，佢要去自己噶所谓噶事业，但佢噶野系天方夜谭	I really don't want to use the term "husband" for him, because he doesn't have any sense of responsibility toward the family. This is a topic I really don't want to bring up, because… how should I put it?<br><br>The word "husband" only applies to him in the sense that I have this daughter, and then when she goes to school, for things like household registration and related matters, his status is needed. And I don't want to hurt my husband too much—meaning I don't want to hurt my daughter too much—so that when

	嘅，佢唔可以系为哩个家庭作出一个任何贡献嘅	she's so young she won't be missing things like a father's love, that kind of thing. Because I feel that if it's something I, as an adult, can bear, then fine.<br><br>He has his own pursuits—I'm not talking about the daughter, I mean the husband—he has his own pursuits, his so-called career. But what he's doing is basically a fantasy, and he can't make any contribution to this family at all.
8. Sense of family	唔系嘅，当你有左细路仔嘅时候咧，你会，你整个人嘅捻法系会唔同嘅，点解咧？啲话可能你依家听我将哩 d 嘢会系好废嘅，但以后，当你有自己嘅家庭，或者自己嘅小朋友，有你自己嘅责任嘅时候，你听返我讲嘅野就会觉得，哎呀，原来系甘嘅，真系甘嘅，点解咧？因为，小朋友系你嘅未来咯，系你心里面嘅未来，明唔明白啊？啲你嘅内心嘅深处，会有一点光，而哩一点光就系小朋友啦，甘当你遇到好，好 sad 嘅野啊或者系你觉得好难接受嘅时候咧，你果 d 光就会起作用嘅啦，佢就会系你心里面撩下撩下你，甘你就会觉得，其实冇问题	No, it's not like that. When you have a child, the way you think as a person will be different. Why? What I'm saying now might sound really pointless to you, but later—when you have your own family, or your own child, and you have your own responsibilities—when you think back to what I'm saying, you'll feel, "Oh, so that's how it is. It really is like that." Why? Because a child is your future—your future in your heart, do you understand? Deep down inside you, there's a little light, and that light is your child. So when you run into something really sad,

	噶哩 d 野，咩野都可以过去噶知唔知啊？<br>冇咩点捻啊，哩个系，必然噶啦(照顾小孩而停下手头工作)，我冇捻其他野，我只会捻哎呀你几时好返啊？点解会甘样噶？我只会捻点同医生沟通啊用咩治疗方法最快啊，冇野啦，仲要捻 d 咩啊	or something you find hard to accept, that light will start to work. It will keep nudging you in your heart, and you'll feel that actually these things aren't a problem—that everything can pass, you know?<br><br>I don't really think about anything else. It's inevitable (having to stop work to take care of a child). I don't think about other things. I only think, "When will you get better? Why did this happen?" I only think about how to communicate with the doctor, what treatment method will be the fastest. That's it—what else is there to think about?
9. Abreaction	系啊，哈哈，冇错，我怼，我边个近我就怼边个噶啦，话比你知啦，哈哈	Yeah, haha, that's right. I snap at people—whoever is closest to me, I snap at them. I'm telling you, haha.
10. Neglect	唔去捻冲突哩 d 问题？（苦笑），唔系话唔去捻，而系话我捻都冇用啊…我捻有咩用啊？边个同我解决啊？<br>边有甘多影响啊小朋友，依家咩野社会，依家社会	Not thinking about conflicts and those kinds of problems? (wry smile) It's not that I don't think about them—it's that thinking about them is useless. What's the point of thinking?

	系求生存嘅社会啊，唔到你捻甘多野嘅	Who's going to solve it for me?<br><br>And it's not like there's that much influence from the kids. What kind of society is it now? This is a society where you're just trying to survive. You don't even get the chance to think about so many things.
11. Emotion	其实哩种，你头先讲嘅冲突啊，对我最，造成最大嘅冲击会系心灵上面嘅冲击咯，啱我个心态，对我来讲会系好 sad 果一霎那	Actually, the kind of conflict you mentioned earlier—what hits me the hardest is the emotional impact, the impact on my mind/heart. In that moment, my mindset just feels really sad for a split second.
12. Mood<br>12.1. Families	甘有时候佢会有压（力），啱佢会有情绪啊，佢嘅情绪会向我去发泄，因为佢只能够向我发泄，甘当我接收到佢嘅情绪嘅时候咧，我就会好受佢嘅情绪嘅影响咯，你明唔明？哩个就系所谓嘅冲突对我做成最大嘅影响啦…我只能够用最快嘅速度，用最极速嘅速度，去稔出，比佢（家人）带来嘅哩种所谓嘅情绪嘅最关键嘅原因系边度，个点系边度，然之后我就好似解结甘样，去解返开佢，即刻我系要即刻，否则嘅话我成日嘅情绪都系会好低	Sometimes she's under pressure—she'll have emotions, and she'll vent those emotions at me, because I'm the only person she can vent to. And when I receive her emotions, I get really affected by them. Do you understand? That's the biggest impact that this so-called conflict has on me.<br><br>I can only, as quickly as possible—at the fastest speed—figure out the key reason

	落，同埋我会工作上面会受影响	behind the emotions she (my family) brings, what the critical point is. And then I have to "untie the knot," like solving a knot, and untangle it immediately. I have to do it right away; otherwise my mood will stay very low all the time, and it will affect my work.
13. Employee	我好幸运咯，我有，啱我依家只系有 2 个员工，因为哩个疫情后我冇办法再请更多嘅人啦，甘我只能有 2 个员工，而哩 2 个员工咧，都系跟左我好耐嘅，甘其中一个咧，我好幸运地系佢会将我哩个生意佢会当成系佢自己，佢自己嘅一个事情咯，啱完全系当左系自己嘅嘢甘，毫无保留甘系为我，企系我个角度甘帮我去做，而且系肩负系最困难嘅嘢，肩负起其他人唔会去做嘅野，甘哩样野系佢对我嘅帮助好大啦，工作上面	I'm very lucky. Right now I only have two employees, because after the pandemic I haven't been able to hire more people, so I can only keep two. And these two employees have been with me for a long time.<br><br>One of them in particular—I'm really lucky—treats my business as if it were their own, like it's their own matter. They completely treat it as their own, and help me without holding anything back, always standing in my shoes and helping me do things. They also take on the hardest tasks—the things other people wouldn't do. That helps me a lot, especially at work.

14. Work is for family	工作为家庭服务）甘绝对啦，唔通为我服务咩？为我自己服务嘅我唔做嘅啦我训嘅了	(Work serves the family) Absolutely. What, should it serve me? If it were just serving myself, I wouldn't do it—I'd just go to sleep.

Interviewee 11

Male, 26 years old, IT business owner, flexible working hours, income – 50,000 to 100,000 RMB per months; single, living with parents, parents still working fulltime, will pay live expense to his parents sometime. (28 significant statements)

1. Nature of the work	第一点我唔系学医嘅第二点我唔系学计算机嘅，所以我就，对哩方面其实好多野都要指意佢地（员工），啱老老实实讲一句好多野都要指意佢地…<br>啊，其实我经常会有哩d，哩d，呃，感觉嘅，因为就住就住佢地，跟住就，最困难果d基本都系我系度做啊嘛，包括同d甲方去交涉啊，合同啊，乱七八糟啊，其实，好多时我都有个感觉就系，我系打杂嘅，佢地先系老板（苦笑）<br>其实工作上面嘅压力，百分之90都系人际关系嘅压力…客户嘅关系其实仲算OK，主要系内部嘅问题，会严重d，啱我依间公司来讲，主要系内部嘅问题会严重d	First, I didn't study medicine. Second, I didn't study computer science. So for this area, there are a lot of things I have to rely on them (my employees) for. Honestly, there are so many things I have to rely on them for…<br>Actually, I often have this… this feeling, because I'm relying on them, and then the hardest parts are basically done by me—like negotiating with the client side, contracts, and all that messy stuff. A lot of the time I feel like I'm the one doing all the odd jobs, and they're the real bosses (wry smile).<br>Actually, 90% of the pressure at work comes from interpersonal relationships…

		relationships with clients are actually still okay. The main issues are internal ones, and those are more serious. In my company, the internal problems are the more serious ones.
2. 亚健康	就非常非常压抑咯，好似有 d 抑郁症果种感觉咯，抑郁啊，焦虑啊，训唔着啊，跟住就比较暴躁咯<br>身体上会虚好多，会虚弱好多...感觉自己虚弱左好多，好容易病...会有 d 话就会头晕啊，头痛啊，跟住成个人好似病濑濑甘啊，	It feels extremely, extremely repressed—kind of like having depression. Depressed, anxious, unable to sleep, and then more irritable.<br><br>Physically I'd feel much "weaker," much more run-down… like I've become a lot weaker, get sick easily… and things like dizziness, headaches, and my whole body feels like I'm unwell and drained.
3. Abreaction	非常影响，因为我地系出面都系要比较温和待人啊，笑口迎人系咪？返到屋企就会表现出最真实嘅自我，最真实嘅自我就，就会，会沮丧 d，沮丧 d，其实一个人，一个家庭当中，一个人低落嘅话其实成家都会跟住低落嘅	It affects things a lot, because when we're outside we have to treat people relatively gently, right? Always smiling and being friendly. When we get home, we show our most real self. And that most real self can be more depressed, more down.<br><br>Actually, in a family, if one person is low or down, the whole family

		will end up feeling down too.
4. Emotion at work	比如话我，或者我老豆，或者我老母，任何一方，生活上，如果工作上受到左委屈啊，唔开心啊，其实成家都会受到影响嘅，甚至会有 d 好细微嘅生活琐碎，会大吵一架都好正常嘅，都比较常见嘅	For example, whether it's me, or my dad, or my mom—any one of us—if in life or at work we've been wronged or treated unfairly, or we're unhappy, the whole family will be affected. Sometimes even tiny everyday trivial things can lead to a big argument—that's totally normal and quite common.
5. income	原先我有段时间创业啊嘛，甘压力大，训唔着，各方面果 d 比较压逼啊，各方面果 d 指标，追的我比较紧，压逼，跟住又有 d，点讲咧，就公司现金，现金流出现左问题，果段时间压力就最大嘅果段时间系，郁唔郁就发脾气，郁唔郁就发脾气，扔野啊果时候系	At one point I was starting a business, right? The pressure was huge—I couldn't sleep, everything felt really oppressive. All kinds of targets/metrics were pushing me hard, and then… how should I put it… the company's cash—cash flow—had problems. That period was the most stressful.<br><br>I would lose my temper at the slightest thing—at the slightest thing I'd blow up. I even threw things during that time.
6. guilty	其实我系好内疚，但系我系控制唔到，果段，如果人啊，陷入到果种情绪果种生活啊，其实系一种漩涡来嘅，系自己好难走到出来嘅，其实自己知道唔	Actually, I felt very guilty, but I couldn't control it. During that period, when someone falls into that kind of emotion and that kind of life, it's like a

	好，好内疚，但系就控制唔到自己，会，陷入一个，唔好嘅一个循环入边嘅	whirlpool—it's very hard to pull yourself out. You know it's not good, and you feel very guilty, but you still can't control yourself. You end up trapped in a bad cycle.
7. Self-regulation	最尾都系，唉冇办法，最尾都系，需要自己慢慢去调节，真系需要自己慢慢去调节...最主要最主要嘅都系要自己捻的通自己去拆掂佢自己去调节	In the end it's… sigh, there's no way around it. In the end you still have to slowly adjust yourself—you really have to slowly adjust yourself. The most important thing is that you have to think it through yourself, sort it out yourself, and regulate yourself.
8. Family of origin<br>8.1. Financial statue<br>8.2. Parents support	家庭生活中，物质上面就完全 0 压力，物质上系冇压力<br>同我会讲 d 比较，比较慰问性嘅野咯，就会，就算创业唔成功，屋企都唔会话太差啊之类果 d 咯<br>好似我老豆成日会讲一句话，哎呀，搞唔掂唔紧要嘅冇问题嘅<br>其实我系冇能力，因为父母工作还唔错，都算唔错，所以我暂时未有话太多能力可以话照顾到佢地咩野...因为佢地嘅能力啊各方面啊，资源啊各方面比我犀利好多，所以我都冇话有咩能力啊有咩资格啊可以话去照顾到佢地嘅	In family life, materially there's completely zero pressure—there's no financial/material pressure.<br><br>They'll say some more comforting things to me, like: even if the business venture doesn't succeed, the family won't be in too bad a situation, things like that.<br><br>My dad often says, "If you can't manage it, it's okay—no problem."

		Actually, I don't really have the ability, because my parents' jobs are pretty good—quite decent—so for now I don't really have much ability to say I can take care of them or anything. Because their capabilities, resources, and so on are much stronger than mine, so I don't feel I have any ability or qualification to say I can take care of them.
9. Flexible working hour	其实系帮助好大嘅，其实真系讲真果句真系帮助好大嘅，唔需要上班啊，落班打卡，唔需要打卡啊嘛因为，因为都系人地打卡比我所以我就唔需要话太咩嘅	It's actually a huge help—honestly, it really is a huge help. I don't need to go to work and clock in or clock out—I don't need to punch a time card, because someone else clocks in for me, so I don't really need to worry about those things.
10. Sense of family	当时啊？更多系紧张屋企咯，屋企人身体咯，其实工作果 d 唔多紧要嘅，对个屋企人来讲，其实工作唔值得一提嘅	At that time? I was more worried about home—about my family's health. Work wasn't that important. When it comes to family, work isn't really worth mentioning.
11. Work is for family	工作啊？为左更好嘅生活咯，其实老老实实系为左更好嘅生活嘅，就系讲，讲白 d 就系更多钱嘅嘛…以家庭为主，唔系以个人	Work? It's for a better life. Honestly, it's for a better life—plainly speaking, it's for more money. It's family-oriented, not centered

	噶生活噶，以家庭为主噶生活	on my personal life—family comes first.
12. Conflict with families	比如就话本身一 d 唔值得一提噶事大家就吵翻场，之后第二日上班大家就唔开心甘，甘上班出门之前或点样吵翻场，甘都会影响一日噶心情噶...就会比较烦躁 d 咯，感觉好似唔顺利甘，感觉日子好似淡而无味甘...啱觉得自己点解，呃，出来做野好似唔知为乜甘突然间	For example, over something that really isn't worth mentioning, everyone ends up having a big argument. Then the next day, everyone goes to work unhappy. If you have a big quarrel before heading out to work, it affects your mood for the whole day… you feel more irritated, like things aren't going smoothly, like life feels bland and tasteless… and you suddenly feel like, "Why am I even out here working? What am I doing it for?"
13. Single child	其实我到哩个年纪 26 岁系咪，话大唔大，话细无细，慢慢都要过度承担更多责任噶啦，(照顾父母) 哩个系必须噶...兼且系独生子女啊嘛，所以就，哩个系正常噶，哩个就会慢慢过渡咯	At my age—26, right—you're not that old, but you're not that young either. Gradually you have to transition into taking on more responsibilities. (Taking care of parents) That's necessary… and I'm an only child, so it's normal. You just gradually move into that stage.
14. Take care parents	其实系一种压力来噶，因为捻住话侬家好多野就唔系话做的太好啊，未有话完成到目标，呃，经济上面都唔会话特别宽松，甘老豆老母又一直变老系咪？其实更多噶系压力来	It's actually a kind of pressure, because you're thinking that right now many things aren't going that well—you haven't really achieved your goals yet, and financially things aren't especially

	嘅，唔系话动力，系压力来嘅…佢地系冇其他人可以依靠啊嘛，父母啊，长辈果 d	comfortable. And your dad and mom are getting older, right? So it's more pressure, not motivation—it's pressure… because they don't have anyone else they can rely on—your parents, the elders and so on.

Interviewee 12

Female, 42 years old, nurse (work in Macau, live in Zhuhai), three eight-hour shifts, rest 2 days per week, income – around 33,000 RMB per month; married 10 years, has two girls, both 6 years old, living with husband, children, and a nanny, husband work from home (stock investor)

1. Nature of the job	假如个病人，啲果日嘅病人，比较平稳嘅 d 病情，因为我依家主要系修神经内科同外科照顾哩 d 病人，甘，内科嘅咧，就老人家多，啲病人咧，容易会系跌亲啊或者系，呃，遇到话高血压啊，或者系中风嘅病多啦，啲生活护理比较多啦，另外一组咧就脑外科，脑外科嘅病咧就会脑出血啊，手术啊，亦都急须，缓急咧系非常之，呃，都会具备咯，啲话每一天面对嘅野都系唔同嘅挑战性系唔同咯，会影响自己嘅情绪，啲特别你好紧张果时，你系惊佢嘅生命，或者自己配合的唔好啊，或者系冇即使发现啊，自己嘅情绪都系会受影响甘咯	If the patient—the patients that day—are in a relatively stable condition… because right now I mainly rotate through neurology/internal medicine and surgery to take care of these patients.<br><br>For internal medicine, there are more elderly patients. Their issues are often things like falls, or conditions like high blood pressure, or strokes. So there's a lot of daily-life and personal care.<br><br>The other group is neurosurgery. In neurosurgery you see things like brain hemorrhages,

		surgeries, and the urgency can be very intense—sometimes it's extremely critical. So every day you face different things, and the level of challenge is different. It affects your emotions. Especially when you're very nervous—you're afraid for the patient's life, or you worry that you didn't cooperate well, or that you didn't detect something in time—your emotions will definitely be affected.
2. Flexible working hours	甘有时咧，小朋友佢学校会安排一 d 活动啊考试啊或者家长会啊，甚至系 d 校运会啊，需要系 d 亲子活动啊，因为我系三班噶原因，甘唔一定可以调到班去参与小朋友 d 学校噶应该参与噶野咯	Sometimes the school will arrange activities, exams, parent meetings, or even sports days—things that require parents to participate, like parent–child activities. But because I work shifts, I can't always swap shifts to take part in the school things I'm supposed to attend for my child.
3. Abreaction	甘有时返来咧，小朋友要教育啦，因为小朋友仲细啊嘛，甘佢地返来要教佢地做作业啦，特别系岩岩我地小朋友咪读小学噶，佢从一个环境适应一个新噶环境，甘有时返来教佢，明明好似觉得，我地觉得，大人觉得好简单噶一个问题，系佢身上小朋友唔一定觉得简单，甘有	Sometimes when I come back, there's the child's education. The kids are still young, so when they come home you have to teach them to do their homework—especially since my child has just started primary school. They're adapting from

	时就会动粗口，唔系粗口，啲系话大声咯，甘都唔识？我教左你好多次了喔！啲觉得自己好简单，甘个语气上可能会收唔到咯，明嘛？啲话我会好似讲，哩个明明系 2 加 2 系等于 4 噶，甘我用另外一个方式讲比佢听，但佢就唔明白，跟住唔明白你就会容易去，起钢咯	one environment to a new one.<br><br>Sometimes when I'm teaching them, something that adults feel is very simple might not feel simple to a child. Then I might end up raising my voice—not swearing, but speaking loudly—like, "You still don't understand? I've taught you so many times!" To us it feels so simple, so my tone might get out of control, you know?<br><br>Like, I might say, "This is obviously 2 + 2 = 4," and I try explaining it in another way, but the child still doesn't understand. And when they don't understand, you can easily get frustrated and lose your temper.
4. Child<br>4.1. Education<br>4.2. Age of child	一直落来 10 个钟头你先翻到屋企，啲其实你都需要休息噶，休息系包括脑放松同身体噶放松，甘你返到来连食饭时间都好匆忙，跟住连自己噶身心都未充分放松噶情况下你就要教育小孩，啲自己噶子女噶情况，你就觉得力不从心咯…啲唔耐烦咯，唔耐烦.	After being out for 10 hours, you only get home then. Actually, you also need rest—rest includes relaxing your mind and your body. But when you get home, even mealtime is rushed, and before you've fully relaxed physically and mentally, you already have to teach your

	因为你惊教佢唔好啊嘛，惊佢青春期嘅反叛啦，惊自己嘅能力教唔到佢啦，或者系唔识教佢啦，或者系，就系惊，系咯，就系惊，应该系惊哩 d 野多 d（笑）	child. With your own child, you end up feeling powerless… you become impatient—impatient.<br><br>Because you’re afraid you won’t teach them well. You’re afraid they’ll rebel in adolescence. You’re afraid you don’t have the ability to teach them, or that you don’t know how to teach them. Or… you’re just scared—yeah, just scared. I guess it’s mostly fear about these things (laughs).
5. 亚健康	就觉得，甘你首先累啊嘛，你累嘅话咪系会影响到你，呃，啱你果个休息嘅野你得唔到休息你嘅大脑就会好累咯，跟住你脾气一唔好嘅话你血压咪会升高咯，你长期 d 血压高，你情绪唔稳定，你咪会产生一 d，呃，压抑啊或者系觉得自己，顾唔掂啊，顾唔过来啊，甘长期嘅话就会影响自己个内分泌平衡果 d 嘅嘛…会有头痛咯，同埋咧，呃，训得唔好咯…啱容易早醒咯，啱你训到有时候 3，4 点啊或者系因为自己单位要考试啊，或者系检查啊，甘你果日就会好紧张啊，你就训得唔好啊，你就要提前去起身去准备啊，d 书未背嘅或者你有时甚至	You feel that, first of all, you’re tired. And when you’re tired, it affects you—if you don’t get the rest you need, your brain gets really exhausted. Then if your temper is bad, your blood pressure can rise. If your blood pressure stays high for a long time and your emotions are unstable, you might develop things like feeling repressed, or feeling like you can’t handle it, like you can’t keep up. Over time, it can affect your endocrine/hormonal balance and so on. You might get headaches, and also sleep

	3，4 点起身都要背书啊甘样咯	poorly—like waking up early easily.<br><br>Sometimes you sleep until 3 or 4 a.m., or because your workplace has exams or inspections/checks, you feel very nervous that day and you don't sleep well. Then you have to get up early to prepare—if you haven't memorized the material yet, sometimes you even have to get up at 3 or 4 a.m. to study.
6. Guilty	甘紧系唔好啦，因为觉得佢本身，呃，好似佢依家系岩进入，佢从一个幼儿园噶小朋友变左一个学生，佢唔识亦都好正常，加上佢好多唔识都系正常噶，但当你发脾气同闹情绪果阵时你冇捻到哩样野，你就觉得佢点解唔识？你就会反问佢你点解会唔识，唔会话，啱一下子可能唔会话系佢个角度果度捻，你就会觉得啱系甘简单你都唔识，但系甘会觉得点解个小朋友做咩会甘蠢啊，或者系，或者慢慢捻下捻下，啱事后之后都会觉得系自己唔岩噶，但系就当时系控制唔到咯，事后会觉得会反思返，唉，都唔岩噶，应该都要心平气和噶，就算几辛苦都好，知道要先自己	Of course it's not good. Because you know that she's just entered a new stage—she's gone from being a kindergarten kid to being a primary school student. It's totally normal that she doesn't know things. And it's normal that there are many things she doesn't know. But when you lose your temper and get emotional, you don't think about that. You just think, "Why don't you know this?" You even question her: "Why don't you know?" You won't immediately think from her perspective. You feel like, "This is so simple and you still don't know it," and you might even think,

	调节好自己嘅情绪先再去教小朋友甘咯，	“Why is this child so stupid?” Or… later, after thinking it over, you’ll realize you were wrong. But in the moment you can’t control it.<br><br>Afterwards you reflect: “Sigh, that wasn’t right. I should have stayed calm.” No matter how hard it is, you know you have to first regulate your own emotions before teaching the child.
7. Family support	觉得自己先生不作为咯，啱另外一半，帮你分担嘅野唔得咯，分担唔到咯，所以你会，几种野会加埋一齐咯	You feel that your husband isn’t doing his part—your other half can’t share the load with you, can’t help you shoulder things. So several issues pile up all together.
8. Family needs	会嘅，啱话你，一翻到来咧，你咪会，呃，要陪个小朋友陪到好似 8，8 点钟先翻到来，我自己食完饭都半个钟啦，都好赶咯，每一日都觉得自己嘅时间好赶，陪完之后咧，你，要拿返成一个钟头时间去教佢地，检查功课，教完之后咧你有时，本身计划咧，系 10 点钟前要训觉嘅嘛小朋友，但通常往往都要拖到 11 点先可以真正系训系张床度咯，甘但系你，呃，因为 11 点进入张床之后咧你唔系	Yes. For example, when you get back, you have to accompany your child until about 8 p.m. By the time I finish eating, it’s already half an hour, and everything feels rushed. Every day I feel like my time is so tight.<br><br>After spending time with them, you still have to set aside about an hour to teach them, check their homework,

	即刻可以训到噶嘛，甘你仲要可能会处理下自己手机啊或者放松下，有时会听下音乐啊，或者听下 d 新闻啊，甘你一般都系 12 点先训，甘但系你训完之后如果你睡眠质量唔好，甘你训 4 个钟啊 5 个钟啊甘你咪唔够咯 …有时会，休息得唔好咧，可能自己工作上噶操作咧，就唔系做到甘完美咯，啱有 d 系侵入性噶好似你就话我系护士打针甘啦，啱唔一定系一次会成功啊甚至有时咧，有时对野果时啊，或者都会走神甘样咯，啱工作上有 d 核查工作时会走神，都要睇多几次啊，平时可能一次两次你觉得 OK 你要睇几次先可以敢确定到甘咯	and so on. After that, you might have planned for the child to sleep before 10 p.m., but often it gets dragged out until 11 before they’re actually in bed. And even when you get into bed at 11, you don’t fall asleep immediately—you might still handle things on your phone or relax a bit, sometimes listen to music or the news. Usually you only fall asleep around 12.<br><br>But if your sleep quality isn’t good, and you only sleep four or five hours, then it’s not enough. Sometimes if you don’t rest well, your work performance won’t be as “perfect.” Like, for example, if I’m a nurse giving injections, it’s not guaranteed to succeed on the first try. Sometimes when you’re dealing with something, your mind might wander. Even when doing checks and verifications at work, you might be distracted and have to look several times—normally once or twice feels okay, but then you need to check multiple times before you dare to confirm it.

9. Health of family<br>9.1. Parents<br>9.2. Child	(父母的病对工作)影响唔大，因为佢病情，啲佢地嘅病系慢性病，唔系话果d急性病啊或者甚至需要住院果d啊，假如啊，如若佢发生左d身体比较严重嘅损害啊或者系更大嘅病情咧，甘可能如果佢地系药住院啊，甚至系更加差嘅话咧，甘一定系会有影响咯，影响d咩野咧，就觉得会影响，啲因为照顾唔到，甘你又要花时间去照顾佢甘就可能要请假啊，要同领导批，要请示啊哩d方面调假，可能哩d会有影响咯.<br>啲你顾住有时系打电话去理解情况啊，或者有时佢唔识啊系度喊果时啊，系电话度喊（笑）果时啊，你会觉得影响你当时嘅情绪，你可能要将当时手头上嘅工作放低，先处理家庭嘅情况，跟住处理好之后再返工，所以你系一边上班挂住屋企一边就要，系要处理工作上嘅压力果d.	(How my parents' illness affects work) It doesn't have a big impact, because their illness is a chronic condition—it's not an acute illness, and it's not something that even requires hospitalization.<br><br>But if they were to suffer more serious physical damage or a more severe condition, then it would definitely have an impact. What kind of impact? For example, because you can't take care of them, you'd have to spend time caring for them, so you might need to take leave, get approval from your supervisor, request time off—those things could be affected.<br><br>Also, sometimes you're calling to understand the situation, or when they don't know what to do and they're crying—crying on the phone (laughs)—it affects your emotions in that moment. You might have to put down the work you're doing to handle the family situation first, then after it's dealt with, go back to work. So you're at work while

		worrying about home, and at the same time you still have to handle work pressure.
10. Family of origin<br>10.1. Financial statue	会噶，啱，呃，因为都之前有听过其他，呃，家庭咧，啱自己 d 同事甘，成日都话，唉父母因为冇完善噶社补啦，冇医疗啦，冇退休金啦，啱佢地，当佢地父母一发生 d 咩事佢地就会好焦虑咯，但我暂时系冇甘噶焦虑，因为佢地噶身体状况同埋佢噶收入情况系比较安稳安定噶	Yes. Because I've heard other families—like some of my colleagues—often say, "Sigh, because their parents don't have 完善的社会保障 (complete social security), no medical coverage, no retirement pension," so when something happens to their parents, they get very anxious. But for now I don't have that kind of anxiety, because my parents' health condition and their income situation are relatively stable and secure.
11. Take care of parents	都会（担心以后要照顾父母）噶，因为，呃，啱系，其实，主要系时间上个付出咯，啱我觉得因为佢，头先咪讲左话佢社保啊，各方面都 OK 噶？但佢老人家身体都会有变化噶嘛，你自己身为子女噶，都要去照顾佢噶嘛，唔系话钱方面，钱方面系另外一方面，但感情上噶付出同埋你关怀，关照咧系更加需要，因为年纪大左其实好多时候系需要子女系身边咯，包括，对佢，精神上噶安抚啦，同	Yes, I do worry about having to take care of my parents in the future, because… actually, it's mainly the time you have to put in. Like I said earlier, their social security and everything is okay, but an elderly person's health will still change over time. As their child, you still have to take care of them. It's not just about money—money is another matter. But the emotional investment, and your care and attention, are

	身体上嘅照顾咯，甘，就算系，佢，就算系住医院都好啦，佢都唔会系长期住医院嘅嘛，佢翻到屋企嘅话就需要咯.<br>(将来要照顾父母系)压力嘅，都有 d 压力嘅，啲话惊时间嘅陪伴啦，因为你依家仲系工作嘅拼搏期啊嘛，如果可能啲话惊照顾父母咧，惊影响到工作甘你咪冇收入咯，甘有时甚至可能因为照顾唔到，自己子女方面又照顾唔到甘你可能就会考虑停薪留职啊，你咪会影响到你嘅收入，甘你成个家庭嘅运作你咪会受影响咯.	even more necessary. Because when people get older, a lot of the time they need their children to be by their side—like giving them emotional comfort, and taking care of them physically. Even if they're hospitalized, they won't stay in the hospital forever; once they go home, they'll need support.<br><br>Having to care for parents in the future is pressure—there is some pressure. Like worrying about having enough time to be with them. Because you're still in the stage of fighting for your career and working hard. If taking care of your parents affects your work, then you won't have income. Sometimes you might even think about taking unpaid leave, and that would affect your income. Then the whole operation of your family would be affected too.
12. Single child	真系好庆幸，因为依家独生子女个压力好大嘅，因为你依家一个独生子女咧，你可能要照顾 8 个老人家…自己嘅父母，同埋，父母嘅父母，老公方面嘅父母，或者系老公方	I'm really very fortunate, because the pressure on only children nowadays is huge. As an only child, you might have to take care of eight elderly people—your own parents, your parents'

	面噶父母噶父母，所以系每人要要照顾 4 个老人家，你 2 公婆，啲 2 个人要照顾 8 个人，但依家，因为有 d 人长寿左啦嘛，系嘛？甘有 d，甘我系特殊情况，因为我先生果方面父母就，呃，因为先生年纪比较大，所以父母都走左啦，所以我系，轻松就轻松系哩一个位置，就完全冇哩方面噶压力咯，加上自己父母各方面都仲可以	parents, your husband's parents, and your husband's parents' parents. So each person may need to take care of four elderly people. As a couple, two people have to take care of eight people. And nowadays, because people are living longer, right?<br><br>But my situation is special, because on my husband's side… since my husband is relatively older, his parents have already passed away. So in that sense, I'm "luckier" there—I have no pressure on that side at all. Plus my own parents are still okay in many ways.
13. Cannot meet the child	疫情果阵时咧，系有个特殊噶情况噶，我当时有曾经，呃，有捻住要停薪留职，系因为疫情果时咧，我地，因为，个，封关啊，系封左成半年咧冇翻来见过自己 d 仔女啊，甘就会担心佢地，呃，噶情况咯，完全系，主要系，电话书信上噶联系啊，但系咧，系完全系见唔到，所以就觉得自己好压抑啊，甘果段时间系咯	During the pandemic there was a special situation. At that time I had considered taking unpaid leave, because during the pandemic—because of the border closures—the border was closed for half a year and I couldn't come back to see my children. So I worried about how they were doing. We could only keep in touch mainly through phone calls and messages/letters, but we couldn't see each other at all, so I

		felt very repressed during that period.
14. Family support	非常之庆幸就系，就系屋企仲有人比我可靠咯，啱话你系前线拼搏，后面仲有个支援啊（笑），啱有时唔系话做到完美，但系都可以，啱话缓解下急嘅情况啦，只不过系因为自己嘅担心，令到自己有甘嘅焦虑，甘嘅啱	I'm really very fortunate, because there's still someone at home I can rely on. Like, you're out there fighting on the front line, and there's still support behind you (laughs). It might not be done perfectly, but at least it can help ease urgent situations. It's just that my own worries make me feel that kind of anxiety—that's all.
15. Work is for family	甘因为我地嘅工作，你可以带来高嘅收入啊嘛，所以，哩份工，系压力大嘅，可能比普通果种工种咧系更加辛苦嘅，而且加埋 2 地跑嘅，但我都会愿意去付出，因为就算佢嘅收入，系令到我，呃，嘅计划啊，同埋小朋友嘅照顾啊，同埋未来嘅生活咧，就系得到比较高嘅回报啦，就可以令到以后嘅生活会安稳 d 咯	Because our job can bring in a high income, this job is very stressful. It might be harder than ordinary jobs, and on top of that you have to travel back and forth between two places. But I'm still willing to put in the effort, because the income allows my plans, the child's care, and our future life to get a relatively high return, and it can make life more stable in the future.

Interviewee 13

Male, 26 years old, reporter, flexible working hours, income – 10,000 to 20,000 RMB per month; married 1 years, no child, living with wife, wife work fulltime (16 significant statements)

1. Support at work	像部门的一些领导啊，对我们还是非常关心的，就是帮扶啊，就是进来的时候他有一套完整的，就是，呃，等于说像那种研究生导师，博士生导师一样，他有种那种导师，然后会一把手一把手的帮你带起来，然后，会有轮岗啊，然后每个部门你都，呃，就是集团内部的话都挺熟悉的，整个架构啊，就是进来这么久的话还是对集团这边，架构啊，包括一些方向啊，就是比较熟，比较熟悉了，然后工作上面的话，因为有一些部门领导的关照嘛，所以就还是比较得心应手.基本上都是，正面的，但是，在这个正面的基础上领导对你过分的关心你也可能会有一种，呃，心理上的一种压力，这个压力怎么表现就是说，你在工作里面就可能渴望得到领导的一种认可，然后你就，有这样一种动力，就是想要把工作做的更好，然后写出来的文字更漂亮一点，然后就得到领导的一些肯定	Some of the department leaders are very caring toward us. They provide support and guidance. When you first come in, there's a complete system—kind of like how a master's or PhD supervisor mentors you. There are "mentors" like that who guide you step by step and help bring you up.<br><br>There's also job rotation, so you get to go through different departments, and within the group you become quite familiar with each one. After being here for a while, you become pretty familiar with the group's structure, the overall framework, and some key directions. In terms of work, because certain department leaders look after you, things are still relatively manageable and you can handle them fairly smoothly.<br><br>Overall it's positive. But even on that positive basis, if leaders care *too* much about you, it can also create a kind of psychological pressure. How does that show up? It's that at work you may start craving recognition from your leaders. Then you get this motivation

		to do the work even better, write your documents more nicely, and in return get some affirmation from your leaders.
2. Life habits with spouse	因为大家都不爱做家务	Because nobody likes doing housework.
3. Nature of the job	因为我都是在家嘛，一般有什么事情的话才会出去	Because I'm usually at home—generally I only go out if there's something I need to do.
4. Freedom at work	像工作这一块的话反而就，因为这种时间比较自由的工作它反而会给我家庭里面的一些相处啊就是带来更多自由的时间	As for work, because this kind of job has relatively flexible time, it actually gives me more free time for spending time and getting along with my family.
5. Sense of family<br>5.1. Parents	这个肯定是不好的嘛，因为，呃，和父母的话肯定，我也，我自己也在寻求就是在这个其中找一个平衡点，这个平衡点就是拉近和他们的，这种时空距离吧，然后，呃，空间距离的话我是，我是这么想的，就是以后可能会在工作上面啊进行一些调整啊，然后尽量把自己的工作分配到长沙那边去，然后这样可能会更近点…还有一种可能性就是在这边然后把他们接过来，这也是另外的一种途径	This is definitely not a good thing. Because with my parents, I'm also trying to find a balance in all of this. That balance is about bringing us closer—reducing the "distance" in time and space between us.<br><br>As for the physical distance, what I'm thinking is: in the future I might make some adjustments to my work, and try to arrange more of my work to be in Changsha, so I can be closer to them…

		Another possibility is that I stay here and bring them over to live with me—this is another way too.
6. Communication	2 个人之间啊，我和老婆之间，就是日常交流交谈的一些话题的存在，然后这样反而会，就是让我们有更多的话题可聊，然后也不会就是，把它作为一个客观的，一个社会事件去讨论而已，而不是，就是说我看到这个负面的消息，呃，报道这个负面的新闻啊，然后我个人也变的消极	Between two people—between my wife and me—there are topics that come up in our daily communication and conversations. This actually gives us more things to talk about. And we wouldn't just treat it as an objective, social event to discuss; it's not like I see negative information—negative reports or news—and then I personally become more negative.
7. Emotion at work	就是有一些负面的新闻，遇到这种的话，就在报道之外，我也会有一些个人的情绪，这种情绪会带到家庭里面	When there's some negative news, if I come across that kind of thing, then beyond just reading the report, I'll also have some personal emotions. Those emotions will get brought into the family.
8. Child<br>8.1. Education	作为父母的话你肯定是想他受更好的教育嘛，那他出生，在他出生之前你肯定要给他把这些给安排好，你不能就临时的去做这些准备啊，然后就像学区房啊这些，还有，以后上小学啊上高中啊，你肯定愿意他就上更好的，就得到更好的教育嘛，然后	As a parent, you definitely want your child to receive a better education. So before they're born, you'll definitely want to arrange these things—you can't just prepare for them at the last minute. Like school-district housing, and then later primary school, high school—

	这方面的话还是有一定的压力	you'd certainly prefer for them to attend better schools and get a better education. So in that aspect, there is still a certain amount of pressure.
9. Take care parents	不担心(以后要照顾父母)，因为，现在，现在国内的话就是，养老这方面还是比较完善，他有一个，第一个是社会上面的商业，商业方面他有一些专业的养老院啊，就那种比较专业的那种，而不是那种政府，不是政府辅导的那种养老院，是商业的那种自己交钱，那种，然后另外一种，像社区里面有那种党支部啊，就作为一种社区关怀啊，然后社区里面的一种居家养老，然后还是比较放心吧对以后那种养老关系	I'm not worried (about having to care for my parents in the future), because right now in China, elder care is relatively well developed. First, there are commercial services in society—professional nursing homes of that kind. Not government-run or government-supported ones, but commercial ones where you pay for them yourself.<br><br>And another option is in the community—there are Party branch committees and similar structures that provide community care, including "aging at home" services within the community. So I feel relatively reassured about elder care arrangements in the future.
10.	对于那种，那种，比较普遍的那种，家庭来说的话，夫妻来说的话，可能会是一个问题，但是我觉得这个问题，就对他们的影响的话，应	For more typical families—typical couples—it might be an issue. But I think its impact on them probably wouldn't be especially big; it

	该也不会，不是特别大吧，不会上升到那种夫妻矛盾的那种情况	wouldn't escalate to the point of causing marital conflict.
11. Work is for self	金钱肯定是，比较占主，比较主要的那个影响因素，然后，呃，排在第二的话应该也是，关于一种，社会责任感吧，我觉得，因为，因为目前来说的话，就是在，在这种社会环境下面，你参与到一种社会事件里面，然后参与到那种包括政府啊，然后包括社会的一些进步啊，你参与到这种过程里面的比较，就是比较直接，比较普遍，比较快捷的方式就是做一个记者，或者是做一个，像那种比较公共事业性的那种职业吧，像老师啊，医生啊，还有律师啊这方面的职业，这样你就会有一些，工作里面会有一些个人的成就感吧，这个应该，应该也算是，就是做这份工作的第二点那个，那个叫什么，你提到的那个自我价值	Money is definitely the main and most important influencing factor. And second, I think it's also about a sense of social responsibility. Because in the current social environment, if you want to be involved in social events and in the process of government and social progress, one of the most direct, common, and quickest ways is to be a journalist, or to do a more public-service-oriented job—like being a teacher, doctor, or lawyer. Then, in your work, you can gain a sense of personal achievement. That should count as the second point—the "self-worth" you mentioned.
12. Flexible working hours	因为我们这个工作的话还是比较有时效性的，因为新闻的那个临时性嘛，就是有时候发生的话还是，你不能，就是有其他事情影响你不能就把手头的工作给放下，你还是得把手里面工作给彻底的做完然后	Because our work is quite time-sensitive—because news is urgent and can be very last-minute—so when something happens, you can't let other things affect you and just put down what you're working on. You still have to finish the work in your hands

	交稿，然后忙其他家里面的事情	completely and submit the story, and only then deal with other things at home.

Interviewee 14

Male, 46 years old, work for government (IT department), working hours from 9AM to 5PM, income – around 260,000 RMB per years; married 15 years, living with wife and 2 children, one 14 years old girl, one 3 years old son, wife work fulltime, hired an hourly housekeeper for cooking, (19)

1. Perceived inequity<br>1.1. At work<br>1.2. Gender	因为你在一个岗位工作到一定的时间，本来你认为你应该，要升迁的，但是又轮不上你，很多条件比你差的，甚至不如你的，比你懒的人，他都升迁了，你没升，你会产生一种心理上的很不舒服，就很，就很委屈咯，工作积极性不高，情绪很沮丧咯.<br>家务活他，也没多少家务活，很少的，没什么家务，衣服又洗衣机在洗，拖地扫地又有空就扫一下，没空就不扫，煮饭呢，我们请了一个保姆来帮我们煮饭，煮完饭他就走了，压力不大，没有多少家务.	When you've been in a position for a certain amount of time, you originally think you should be promoted, but it still doesn't happen for you. People with many conditions worse than yours—people who are even less capable than you, or lazier than you—get promoted, but you don't. That can make you feel psychologically very uncomfortable—really wronged. Your motivation at work becomes low, and your mood becomes very down and depressed.<br><br>As for housework, there isn't much. There's very little housework—nothing much. The clothes go into the washing machine, and as for mopping and sweeping, if there's time we do it, if there isn't, we don't. For

		cooking, we hired a nanny to help us cook; after cooking, she leaves. So the pressure isn't big—there isn't much housework.
2. Sense of family	一个家庭里面，男人都是顶梁柱，男人就是事业上要能够有所成就呢，那家庭成员就会很高兴嘛，但是如果说你这个男人事业上你工作老是原地踏步甚至退步了，那家庭里面大家也面子，脸上无光啊，就感觉到没这么舒服咯就是.<br>男人在这个家庭里他顶梁柱，他事业上有成就，那这个家庭，它会快乐会更多，地位会更高，它跟其他人比啊，跟亲戚朋友比啊，它地位会更好，如果说你这个事业上一落千丈，那你自己，也感觉的脸上无光，头也抬不起了，会有这种感觉的.<br>你一个家庭，你像自己，家务活，一般情况下我们这边的传统就是一般老婆做的，他做的好那我不用操心，有帮助（笑）...你时间剩好多啊，你不用为买菜煮饭头痛啊，你会节省很多时间做工作啊，那你的工作表现会更出色，如果你一天到晚都是买菜做饭，那你工作还哪有时间啊？	In a family, men are the main pillar. A man is expected to achieve something in his career, and then the family members will be happy. But if this man's career is always standing still, or even going backwards, then everyone in the family loses face—it doesn't feel good.<br><br>If the man, as the pillar of the family, has achievements in his career, the family will be happier and have higher status. Compared with other people—relatives and friends—the family's standing will be better. But if your career takes a nosedive, then you yourself will also feel ashamed, like you can't hold your head up.<br><br>In a family, regarding housework: traditionally where we are, it's generally the wife who does it. If she does it well, then I don't have to worry—it

		helps (laughs)… You save a lot of time. You don't have to stress about buying groceries and cooking. You can save a lot of time to do your work, and your work performance will be better. If you spend all day buying groceries and cooking, then how would you have time for work?
3. Emotion at work	对个人心情有影响，自己个人心情的影响肯定也会影响到家人的心情，有些没那么，没那么愉快	It affects your personal mood, and your own mood will definitely affect your family's mood too—so things become less pleasant.
4. Sleep quality (亚健康)	有时会想这个事情，是会对身体有影响，因为你事业混的不好，你心情，你那么沮丧，你的睡眠质量没这么高，有时会，夜里会醒来啊，半夜都会醒来，半夜会想起自己工作上不顺心的东西就会，不会经常，偶尔，偶尔会醒来，就偶尔，偶尔想到这些事情，想起工作上的事情	Sometimes I think this can affect the body. Because if your career isn't going well, your mood is so low and depressed, your sleep quality won't be that good. Sometimes you'll wake up at night—wake up in the middle of the night—and you'll think about the things that aren't going well at work. Not often, but occasionally. Occasionally you wake up and think about these things—work-related things.
5. Self-regulation	这个要靠自己，因为你，这些心理上这些，这种不舒服，也不是很大的事情，也不是癌症，你自己	This depends on yourself. Because these psychological discomforts aren't something huge—

	要克服它，其实这些东西靠自己内心靠自己去解决的，不是靠别人，别人是帮不了你的，你睡不了觉别人怎么帮你？你自己内心烦躁，焦躁不安，别人也帮不了你的，要靠你自己去解通，自己想办法去解决，而不是去寻求别人的解决...呃，可能没有了解，因为你再亲的人，他不知道你大脑里在想什么，不是很了解这个，你再亲密的人他也不知道你的想法，你是，脑袋中想的东西，心中焦虑的东西是会变的嘛，你今天想这个明天想那个，他也不知道你在想什么	they're not cancer. You have to overcome them yourself. These things rely on your own inner strength, on you solving them yourself, not on other people. Other people can't really help you. If you can't sleep, how can others help you? If you're feeling irritable and anxious inside, others can't help you either. You have to work it out yourself, find your own way to solve it, rather than seeking solutions from other people…<br><br>And people might not understand, because even the people closest to you don't know what you're thinking in your head. Even someone very close to you doesn't know your thoughts. What you're thinking about, what you're anxious about, can change—you think about this today and something else tomorrow—and they don't know what you're thinking.
6. Freedom at work	我们工作还比较自由的，因为国家单位上班啊，相对说没有比较那么严谨，比如说有点事抽空走开一下也问题不大...比较宽松嘛，他不像那个车间那样，站在那不准动，他不	Our work is relatively flexible, because we work in a government/public institution. Compared to other places, it's not that strict. For example, if something

	是这样的，他是可以中间你有点事出去办点事，偶尔出去办点事，完全可以的	comes up, it's not a big problem to step out for a bit. It's relatively relaxed—it's not like a factory workshop where you have to stand there and can't move. It's not like that. If you have something to handle, you can go out in the middle of the day to deal with it—occasionally going out to take care of something is totally fine.
7. Parent support	(父母会)帮我们煮饭，他会帮我们做饭，他会帮我们带小孩，他不但不用我们照顾而且他们还会帮我们的忙…有什么想法我，这个没什么想法，就这样挺好的，我们中国人的家庭，基本上都是这样，老人都是在一直为你付出，或者，呃，这个是普遍的现象	(My parents) help us cook—they cook for us, and they help us take care of the child. Not only do they not need us to look after them, they also help us… As for what I think about it, I don't really have any special thoughts—it's just fine like this. In Chinese families, it's basically like this: older people are always giving to you. This is a common phenomenon.
8. Child<br>8.1. Education	期望他能考到好的大学，期望他能够学习成绩要好，要有出息，成为对这个社会有用的人才，不能学坏了，这个是肯定有的… 会有压力，就我希望他成绩好点，他考不好我就有压力，考的很差我就有压力咯，这就是压力咯，希望他考个好学校，	I hope he can get into a good university. I hope he can do well academically, have a promising future, and become someone useful to society—he mustn't go down the wrong path. That's for sure.

	他考的学校不行，这个压力就很大（笑）	Yes, there's pressure. If I hope his grades are better and he doesn't do well, then I feel pressure. If he does very poorly, I feel pressure. That's the pressure. I hope he gets into a good school; if the school he gets into isn't good, then the pressure is huge (laughs).
9. Work is for family	(工作是为了)养家糊口，最主要的意义就是养家糊口…也不一定是为了家庭，也会为自己，你自己也要吃啊，也要穿啊，为了家庭也是为了自己，你就有工作你才有收入啊，有了收入你才可以自己生存下去啊，家庭的亲人才可以生存下去啊	(Work is for) supporting the family and making a living—the main meaning is to support the family and make a living… It's not necessarily only for the family; it's also for yourself. You also need to eat and wear clothes. Doing it for the family is also doing it for yourself. Only if you have a job do you have income; only with income can you survive, and your family and loved ones can survive too.

Interviewee 15

Male, 53 years old, work at a sales company, working hours from 9AM to 5PM in general, income – 7,000RMB per month; married 22 years, 2 children, one 22 years old girl and one 13 years old boy, living with wife and both child, wife retired and 2 children still in school (20).

1. Nature of the job	(工作)比较繁琐…工作啊嘛，就，都，你要烦你都要去做噶啦系嘛？要解决到为止噶啦，就哩个情况	(Work) is relatively tedious… work is like that—whether it annoys you or not, you still have to do it, right? You have to

		keep at it until it's solved—that's how it is.<br><br>Maybe it's related to age and experience… It's not exactly that I've "seen through it," more like: as you get older and gain more experience, naturally you start to separate these things—work is work, family is family. So it becomes natural: once you're home, family matters are family matters, and it doesn't affect things that much anymore.
2. Age	可能，可能年纪方面，阅历各方面嘅关系咯…都唔系话睇开左，好似，自然，可能果 d 随左年龄嘅增长同阅历嘅增长，自然自然就会分开哩方面嘅野嘅，工作归工作，家庭归家庭，就会，自然就返左去就，就家庭嘅事就家庭嘅事，就唔会好多影响了	Maybe it's related to age and experience… It's not exactly that I've "seen through it." It's more like: as you get older and your experience grows, you naturally start separating these things—work is work, family is family. So it becomes natural: when you're home, family matters are family matters, and it won't affect things that much anymore.
3. Workload	就我地有时，呃，上班时间比较忙，加班或者系咧，有时出去对外果 d 有 d 联系，就开车时间长，d 开车，有时返去就唔想做野，食完饭就净系想休	Sometimes we're quite busy during work hours, working overtime, or we have to go out and handle external contacts. The driving time can be

	息，就坐系度，躺系度，哩种情况有	long. Sometimes when I get home, I don't feel like doing anything. After dinner I just want to rest—sit there or lie down. This kind of situation happens.
4. Family support	佢地，佢一般咧，都睇到我唔想郁就会问下，系咪好累啊？	They usually see that I don't want to move, and they'll ask, "Are you very tired?"
5. Understanding	都理解咯，依家，出去做野，做份野出面，比较辛苦咯，有时咯，都，哩种情况你系，长期就返去唔做嘢就唔得，你明显，果d收衫，以前来讲，你日日食左饭会收衫会叠下，果日咧你唔叠就会唔同，佢就会感觉到咯，都会互相体谅下咯	They understand. Working outside is pretty tough. But if this goes on for a long time—coming home and not doing anything—that won't work. It's obvious: like, taking in the laundry—before, you'd normally bring in the clothes after dinner every day and fold them. If one day you don't, it's different, and they'll notice. So we try to be understanding of each other.
6. Work achievement	因为除左可能年龄噶增长啊，后生果时都有，都有比如讲年轻岩出来果时好想进一步，几个咩去做d咩嘢，然后好想做d工作之外d嘢比如讲有冇其他d嘢，能够赚d钱噶方法，以前都有，想办法，或者，提高，就会提高啦，或者去d待遇好d噶工作啦都有，可能除左年龄增长哩方面可能淡了	Maybe it's because, besides getting older, when you're young—especially when you've just started working—you really want to improve, you want to do more things. And besides your job, you also want to find other ways to earn money. I used to think like that too—trying to find

		ways, to improve, to move up, or to look for jobs with better pay and benefits. But maybe as I’ve gotten older, that desire has faded a bit.
7. Sense of family	啲女噶咧，有份工作唔好太忙，能够顾到家，甘就最好噶啦，完全系屋企做家庭主妇又唔系几好咯，就会同社会脱节咯，啲唔好要一份太忙碌噶工作，比如讲，啲好多服务行业啊，要做到，上夜班啊甘噶情况，甘就会影响到家庭咯，甘噶情况咯	For women, it’s best to have a job that isn’t too busy and that still lets you take care of the home—that would be ideal. But being a full-time housewife at home isn’t that great either, because you can become disconnected from society.<br><br>So it’s better not to have a job that’s too demanding—for example, many service-industry jobs require working night shifts, and that would affect the family. That’s the situation.
8. Guilty	但系自己仲有一种感觉，感觉到好惭愧咯，就系冇尽到尽仔女噶一种责任咯，对佢（父母），呃，就，(每次回乡下)比小小（钱）佢地又唔系话好多，有哩种自己有感觉惭愧果 d 咯	But I still have this feeling—feeling very ashamed—like I haven’t fulfilled my responsibility as a child toward them (my parents). Like, every time I go back to my hometown, I give them a little bit of money, but it’s not a lot. I still feel that kind of guilt and shame.

9. Child<br>9.1. Age of child	就系有，你睇佢，佢以后读书，工作，我就话假如佢唔生性，佢嘅系能力比较差，佢自己就，可能养活自己都比较难，佢以后，你特别系城市，佢自己买房果 d 佢都有困难噶话，甘啊，甘咪要帮助佢咯，但系。。。。要帮助果 d，但系，你冇果 d 能力，或者果 d，你（就只能）尽自己所能噶啦哩 d 野，系嘛？	Yes—like, you look at your child: in the future, with school and work, if they don’t work hard / don’t turn out well, and their ability is relatively weak—maybe even making a living for themselves is difficult—then later on, especially in a city, if they have trouble buying a home and so on, then you’d have to help them. But… you’d want to help, yet if you don’t have that ability, then you can only do your best within your means, right?
10. Single child	哩方面我就最感谢就系我屋企噶大姐咯，帮我照顾我老豆，7 年咯，冇咩，好，好多，好多抱怨咯，就系主要系哩个咯	In this regard, the person I’m most grateful to is my eldest sister at home. She has helped me take care of my dad for seven years, with hardly any—many—complaints. That’s mainly it.
11. Work is for family	工作系为左生活...为左生活就系为左家庭啦，家庭噶生活啦，就唔会系为左自己噶生活啦，为左生活就系为左家庭噶生活啦	Work is for living… and living is for the family. It’s for the family’s life, not just for my own life. Living is for the family’s life.

Interviewee 16

Male, 41 years old, work at IT company, salesman, flexible working hours, no fixed income, only commission; married 12 years, 1 girl, 6 months (has congenital heart disease), living

with wife, daughter, retired father-in-law and mother-in-law moved to their house when the child was born, wife has fulltime job, still in maternity leave (20)

1. Health of family<br>1.1. Child	就拿今年生小孩的事情来说，其实，像我，可能每个家庭不一样，像我现在今年这个小孩本来就是生出来也是遇到了些问题，所以很多时候重心可能就会摆在家庭这一边，因为毕竟我是年纪比较大生小孩，就是好不容易，好不容易现在小孩出来了，现在又遇到一些问题，所以就会，就是，侧重点就会可能会偏向家庭这边.<br>一方面是因为小孩子事情(先心病)焦虑，一方面又，想着自己的工作上的事情，所以，2 边会夹在一起的时候，有时候会感觉到很焦虑	Taking having a child this year as an example—every family is different, but for me, this baby was born and ran into some problems. So a lot of the time my focus ends up being on the family side. Because I had a child at an older age—it wasn't easy, and now the child is finally here, but then there are some issues, so the emphasis naturally shifts more toward the family.<br><br>On one hand, I'm anxious because of the child's situation (congenital heart disease). On the other hand, I'm also thinking about my work. When both sides press in together, sometimes I feel very anxious.
2. Emotion at work	一定会跟自己说不要把这种情绪，叫什么啊，工作上的一些不顺心的事情带到家里来啊，但是人，人心是肉长的嘛，有时候也会多多少少会有影响的<br>那就最简单最直白的，比如说自己很，就花了很多心思，很多时间去跟进的这个项目，就最后到，刚刚到了招标这	I will definitely tell myself not to bring this kind of emotion—what do you call it—work frustrations and things that don't go smoothly into the home. But people are human, right? Sometimes it will still affect things to some extent.

	个环节，但是因为，也可能是因为团队也可能是因为自己的一些原因没有做好或者是疏忽了，最后把这个项目给丢了，那么，那么可能回到家里来你的，就是回到家里来可能就会，就一个时间段啊，可能说是 1 天或者 1-2 天就会觉得很低落，那就做什么事情，容易有脾气有情绪啊，那么就，大概就是这个	To put it simply and directly: if there's a project you've put a lot of effort and time into following up on, and it finally reaches the bidding stage—but because of something the team did, or for some personal reasons, you didn't do well enough or you overlooked something, and in the end you lose the project—then when you go home, for a period of time, maybe one day or one or two days, you'll feel very down. And then whatever you do, you're more likely to be short-tempered and emotional. That's basically it.
3. Tired/lazy	有时候比如说你太累了，家里，也不想做饭了，尤其是 2 个人一起生活的时候，那不如叫外卖或者去外面吃啊怎么样，就不想去做，就这样	Sometimes, for example, if you're too tired, you don't feel like cooking at home—especially when two people live together—then you might as well order delivery or eat out. You just don't want to cook, that's it.
4. Communication	其实，其实我跟我太太还是很多话题的，就是大家都会包括他遇到些事情啊，他工作上的事情，包括我遇到的工作的事情，我们都会，会大家拿出来沟通，就等于说是大家去谈，看看有没有遇到什么样的问题，他有没有遇到什么	Actually, my wife and I still have a lot to talk about. We talk about things she runs into, things from her work, and the things I run into at work too. We'll bring them up and communicate—basically talk together and see whether there are any problems, what

	样的问题，因为当局者迷嘛，旁观者清嘛，但是比如这件事情，他可能自己在其中走不出来，但是他旁边可能，看到比较清楚，可能给你一些建议啊或者说一些什么话疏导，这是挺好的，我觉得我这一点我在家里还挺好的，我太太也是，近几年，就这份工作包括遇到的一些事情，大家都会相互的去倾诉，或者是交流这个事情	problems she's encountered, what problems I've encountered. Because when you're in the middle of it, it's easy to feel stuck and not see clearly, but an outsider can see more clearly. So for example, with something like this, she might not be able to get herself out of it, but someone beside her might see it more clearly and give her some advice or say something to help guide her through it. That's a good thing. I feel I'm pretty good in this aspect at home, and my wife is too. In recent years, with this job and the things we've run into, we've both been willing to confide in each other and communicate about it.
5. Single child	这个很现实的，你一种压力，自己去抗或者有兄弟姐妹一起去抗的话，你的那种，从内心来讲，可能会兄弟姐妹多的可能会轻松一点，就是压力没这么大，有什么事情可以商量	This is very realistic: if you have a kind of pressure and you have to bear it alone versus bearing it together with siblings, then deep down you'll probably feel a bit more relieved if you have more brothers and sisters. The pressure won't be as big, and if something happens, there are people you can discuss it with.

6. Work opportunities	客户对你的认可，他可能是从一件事情，从头到尾做过来他对你的评价，但是是因为某些事情，你没有去做好，然后某个环节或者某个时间段没有做好，那么必然他会对你的一个认可打折扣，那从单一的客户来说那影响倒是不是很大，但是从长远来说，因为这个是，现在这个做客户是讲服务嘛，他会一传十十传百，那可能就会有一种蝴蝶效应，可能你在业界的一个形象或者是一个定位	A client's recognition of you might come from how you handled something from start to finish—how they evaluate you. But if, because of certain issues, you didn't do something well, or a particular step or period of time wasn't handled properly, then naturally their recognition of you will be discounted.<br><br>For a single client, the impact might not be that big. But in the long run, because dealing with clients now is all about service, one person will tell ten, ten will tell a hundred. That can create a "butterfly effect," affecting your image or positioning in the industry.
7. Work for family	其实第一方面(工作)就为了一个家庭	Actually, the first aspect of (working) is for the family.
8. essence	他也是个问题，但不是很大的问题，叫什么，就拿个简单例子来说，你，其实你家庭跟工作其实也是一个天平一样，你没有办法容易做到就是平衡的，那么就，看一个事情的包括你工作的事情，包括你家里的事情的缓冲级别，你就是，只能是灵活的去面对，去处理，	It's also an issue, but not a very big one. How to put it—here's a simple example: family and work are like a balance scale. You can't easily keep them perfectly balanced. So it depends on the "buffer level" of different things—both work matters and family matters. You can only respond and handle

	这个我觉得没有一个很标准的答案，这要看，分事情或者是分什么这样子咯	them flexibly. I don’t think there’s a standard answer. It depends on the situation, on what the matter is.
9. Flexible working hours	近半年是对我的帮助非常非常大，如果说我不是从事这种职业，销售这种职业，我是从事这种朝九晚五或者是 996 或者是上班很固定时间的话，那么我今年可能，我得处理家里这个事情的话，可能我就会很被动很被动	In the past six months it has helped me a lot—really, really a lot. If I weren’t in this kind of job—sales—and instead I had a nine-to-five job, or a 996 schedule, or any job with very fixed hours, then this year, when I had to deal with things at home, I might have been in a very, very passive position.
