# Supplementary material for: “It Is Not Possible to Balance It Easily”: A Phenomenological Study Exploring the Experience of Work–Family Conflict in Contemporary Chinese Society
Source: Behav Sci (Basel). 2025 Dec 30;16(1):63. doi: 10.3390/bs16010063 (PMC12837297; doi:10.3390/bs16010063)
Supplement: Supplementary file 1 [file behavsci-16-00063-s001.zip › Table S3 Audit trail snapshot .pdf]

Table S3. Audit-trail snapshot of the analytic process

Stage of analysis	Description of activities and documentation
**Data collection**	Sixteen semi-structured interviews were conducted in Cantonese or Mandarin by Chen between August and October 2020. Field notes taken after each interview recorded contextual observations and initial impressions.
**Transcription and data preparation**	Verbatim transcripts were produced in the source language and checked by both first authors for accuracy and anonymity.
**Initial engagement with data**	Reflexive journals kept by both first authors to document assumptions, emotional reactions, and analytic decisions. These memos informed later discussions and were revisited during the theme refinement process.
**Coding and horizontalisation**	Significant statements identified and overlapping items removed (Creswell, 2013). Initial codes generated by Chen and reviewed by Cheng for clarity and consistency.
**Code–recode check**	Transcripts were re-coded after a two-week interval to examine the stability of code application; minor discrepancies were discussed and resolved through consensus.
**Theme development**	Codes were grouped into meaning clusters and themes through iterative discussions with the author group. Inclusion–exclusion boundaries defined for each theme (see Table S1).
**Peer debriefing**	Regular author-group meetings were held to review analytic decisions, challenge interpretations, and confirm alignment between data and developing themes.
**Verification and synthesis**	Negative and deviant cases were examined to refine thematic boundaries. Theme summaries were validated with two participants to confirm accuracy and resonance.
**Final documentation**	All analytic versions, code lists, and meeting notes are logged in dated folders forming a complete audit trail. The final theme tree (Figure S1) summarises the structure of meanings identified.
